# Alzheimer's disease: Estimating its prevalence rate in a French geographical unit using the National Alzheimer Data Bank and national health insurance information systems

**DOI:** 10.1371/journal.pone.0216221

**Published:** 2019-05-06

**Authors:** Laurent Bailly, Renaud David, Roland Chevrier, Jean Grebet, Mario Moncada, Alain Fuch, Vincent Sciortino, Philippe Robert, Christian Pradier

**Affiliations:** 1 Université Côte d’Azur, CHU de Nice, Département de Santé Publique, Centre Hospitalier Universitaire de Nice, Nice, France; 2 Université Côte d’Azur, CHU de Nice, Centre Mémoire de Ressources et de Recherche, EA CoBTeK, Nice, France; 3 Direction du Service Médical Régional du Régime Social des Indépendants (RSI) Côte d’Azur, Nice, France; 4 Direction Régionale du Service Médical de la Caisse Nationale d’Assurance Maladie des Travailleurs Salariés (CNAMTS) PACA-Corse, Marseille, France; Universidad Rey Juan Carlos, SPAIN

## Abstract

**Background:**

Reliable epidemiological data on Alzheimer's disease are scarce. However, these are necessary to adapt healthcare policy in terms of prevention, care and social needs related to this condition. To estimate the prevalence rate in the Alpes-Maritimes on the French Riviera, with a population of one million, we present a capture-recapture procedure applied to cases of Alzheimer’s disease, based on two epidemiological surveillance systems.

**Methods:**

To estimate the total number of patients affected by Alzheimer's disease, a capture-recapture study included a cohort of patients with Alzheimer's disease or receiving medications only eligible for use for this condition, recorded by a specific health insurance information system (Health Insurance Cohort, HIC), and those registered in the French National Alzheimer’s Data Bank (“Banque Nationale Alzheimer”, BNA) in 2010 and 2011. We applied Bayesian estimation of the M_t_ ecological model, taking into account age and gender as covariates, i.e. factors of inhomogeneous catchability.

**Results:**

Overall, 5,562 patients with Alzheimer's disease were recorded, of whom only 856 were common to both information systems. Mean age and F/M sex ratio differed between BNA and HIC surveillance systems, 81 vs 84 years and 2.7 vs 3.2, respectively. A Bayesian estimation, with age and gender as covariates, yields an estimate of 15,060 cases of Alzheimer's disease [*95%HPDI*: 14,490–15,630] in the Alpes-Maritimes. The completeness of the HIC and BNA databases were respectively of 25.4% and 17.2%. The estimated prevalence rate among the population over 65 years old was 6.3% in 2010–2011.

**Conclusions:**

This study demonstrates that it is possible to determine the number of subjects affected by Alzheimer's disease in a geographical unit, using available data from two existing surveillance systems in France, i.e. 15,060 cases in the Alpes-Maritimes. This is the first stage of a population-based approach in view of adapting available resources to the population’s needs.

## Background

The prevalence rate of dementia increases exponentially with age [1]. In Southeastern France, this is of particular concern since a 57% increase in the 60 to 80 year-old age group is expected by 2040 [2]. The prevalence and incidence rates of a disease are among the most fundamental measures in epidemiology. Estimates of the number of people affected with a condition are required to convince policy makers and funders of the reality and magnitude of the public health burden, and to help with decisions on how resources should be allocated for better program planning and management. However, obtaining reliable epidemiological data on the general population, such as the number of patients with Alzheimer’s disease or related conditions, is difficult, due to the specific characteristics of these diseases [3].

Capture-recapture models are an indirect method of estimating population size derived from techniques developed for studies of animal abundance [4]. These methods, applied in epidemiology, estimate the true population size by evaluating the degree of overlap among incomplete lists of cases from existing data sources. In the Alpes-Maritimes, an area with a population of one million inhabitants, two sources of information are available regarding patients with Alzheimer’s disease or related conditions: the French National Alzheimer database (BNA), a central information system for a network of memory clinics that has been coordinating the management of demented patients according to national guidelines since 2010 [5]; the PACA-Alz cohort, established in 2008 by the national health insurance information scheme (HIC) on patients exempted from copayment due to dementia on one hand, and those receiving medications only eligible for use in Alzheimer’s or related conditions on the other hand [6]. Applying this method with only two lists has some major limitations. Indeed, a bias in the estimate of the total population under consideration can occur in the presence of positive or negative dependence between sources or when individuals do not have the same probability of being “captured” by the two surveillance systems.

Our study presents a capture-recapture procedure applied to cases of Alzheimer’s and related conditions for which inhomogeneous catchability is suspected. We therefore applied the Lincoln-Petersen estimator and also considered a Bayesian estimation using the M_t_ ecological model [7–9], allowing for factors of inhomogeneous catchability to be taken into account. The objective of our study is to estimate the number of subjects affected by Alzheimer's disease in the Alpes-Maritimes department, using the capture-recapture method.

## Materials and methods

### Medical and administrative sources

The study population consists in all the Alpes-Maritimes residents who visited a specialized physician in a memory clinic in 2010 and 2011, who were included in the health insurance surveillance system because of copayment exemption due to Alzheimer’s disease and related conditions, or who were receiving medications only eligible for use in Alzheimer’s disease during that period.

#### French National Alzheimer database

The French National Alzheimer database is a national network that collects data on Alzheimer’s disease and related conditions [10] to promote descriptive and clinical epidemiological studies. These data are generated from patient visits related to memory disorders. When a patient attends a memory clinic, the physician completes a standard patient file [11], including diagnosis. The French National Alzheimer database is supported by the French Ministry of Health, independently of the national health care system detailed below [12]. There is no exchange of information with any other database and specifically with any health insurance information system. Records have been created under the conditions of the Commission Nationale de l’Informatique et des Libertes (CNIL), responsible in France for data protection, validated in 2009 and use with respect to the human identity and human rights.

#### Health insurance copayment exemption and medications only eligible for use in Alzheimer’s disease

The French health care system is based on universal coverage managed by a national health care insurance system named Social Security. The health insurance system covers most medical expenses including the use of drugs. For the reimbursement of drugs, patients use a smartcard that transmits patient’s identification and drug information to the health insurance automated pharmacy claims database [13]. For all medical expenses there remains a copayment payable by the patient, but for some specific conditions such Alzheimer’s disease insured patients may ask the Social Security for copayment exemption, and if the diagnosis is confirmed all health dispensing is totally covered [14].

The PACA-Alz (Provence-Alpes-Côte d’Azur Alzheimer) cohort is a specific cohort that includes all those insured with the national health insurance scheme who were granted copayment exemption due to Alzheimer’s or related conditions or who were receiving medications only eligible for use in Alzheimer’s disease for those conditions [15]. The copayment exemption and the automated pharmacy claims database sources operate through distinct information channels. Medications only eligible for use in Alzheimer’s disease include EBIXA (memantine), ARICEPT (donepezil), REMINYL (galantamine), and EXELON (rivastigmine). For those medications, French Health Authority recommendations were to conduct a multidisciplinary evaluation one year after their prescription [16].

Our study complied with the terms of service for both databases used, the PACA-Alz cohort and the BNA.

#### Case linkage procedure

The study protocol was submitted for review to the ethics committee of Nice Sophia-Antipolis University. The ethics committee approved the protocol of the study in 2012 and they declared that formal judgment was not required (protocol number: n°2012–0745). No informed consent was required.

The linkage procedure for common cases was performed with the FRIL application [17] (Fine-grained Record Integration and Linkage tool) on family name identifiers. FRIL is a free open-source application developed by the Centers for Disease Control in Atlanta and Emory University, which uses probabilistic links to create links between databases. These links can be tuned to determine their accuracy and performance. The identifiers used for case linkage included surname, first name, married name, gender, date of birth and zip code.

### Estimate of the total number of patients by capture -recapture

Application conditions of capture-recapture in epidemiology require that all identified cases meet the same criteria and are therefore true cases, over a common study period and within the same geographical area. The study population should be a closed one. Only matching, properly identified cases, with a common case definition must be taken into account. Sources must be independent, i.e., the likelihood of a case being identified by one source must not be linked to its identification by another and, lastly, catchability must be homogeneous within sources, i.e., it must not depend on case characteristics. For example, in our capture-recapture model age could be considered as a factor of inhomogeneous catchability because Alzheimer’s disease progresses over several years, consultation in a memory clinic, copayment exemption or prescription of drugs may thus occur at different ages during the disease’s progression.

As only two sources were available, we applied the Lincoln Petersen estimator [18] and the almost unbiased Chapman estimator [4], as detailed in the S1 Table. All relevant data can be found within the paper and its Supporting Information files.

#### Bayesian estimation with the M_t_ ecological model

Ecological models, developed in ecology to estimate the size of animal populations, are based on assumptions concerning the capture of individuals. The M_t_ ecological model considers that each time (t) of capture has its own influence on the capture. Ecological models were subsequently applied to epidemiology, considering that sources influence capture. In a time-dependent, closed capture-recapture model, each individual must have the potential to be caught on multiple sampling occasions. In our capture-recapture model, each individual can be captured and recaptured during any consultation in a memory clinic, or each time a medication only eligible for use in Alzheimer’s disease has been prescribed or a copayment exemption has been granted.

As Alzheimer’s disease progresses over several years, age was suspected to differ between the French National Alzheimer database and the health insurance cohort, as consultation in a memory clinic, copayment exemption and specific prescription may occur at a different age during disease’s progression. Indeed, age and gender differed at baseline between the French National Alzheimer database and the health insurance cohort. To avoid inhomogeneous catchability between individuals in our capture-recapture model, we therefore included age (< 84 years, 85–89 years,> 90 years) and gender as covariates in our model. However, each individual had the potential to be caught on multiple sampling occasions in each of these classes, as he/she could be captured or recaptured during any consultation in a memory clinic, or each time a specific prescription was issued. The advantage of those models resides in the fact that it is easy to include covariates that are suspected to result in inhomogeneous catchability between individuals. However, because of a limited number of subjects, the Frequentist’s asymptotic assumption could not be verified for certain classes. Consequently, we decided to apply a Bayesian approach The M_t_ ecological model and the Bayesian application, as well as the WinBUGS code [19], are detailed in the appendix. Bayes’ theorem is used to estimate the joint posterior distribution of all the parameters included in the model. Ultimately, the posterior marginal distribution of the parameters of interest is estimated, requiring integration of the posterior joint distribution. A Monte Carlo Markov Chain algorithm is applied to obtain a random sample of the posterior distribution. Monte Carlo integration is a simulation technique which provides an estimate of a given integral. This technique is based upon drawing samples from the distribution of the variable of interest and then calculating the sample mean. To obtain a potentially large number of samples from the posterior distribution we use a Markov chain, which is a stochastic sequence of numbers where each value in the sequence depends only on the previous one. The Metropolis-Hastings algorithm was used to perform Markov chains with Monte Carlo integration to generate observations and to construct a sequence of values whose distribution converges to a stationary distribution, providing the chain is aperiodic and irreducible. Once the chain has converged we can use a sequence of values to obtain estimates of any posterior summaries of interest (Monte Carlo). To make sure that the Markov chain has reached the stationary distribution before providing Monte Carlo estimates, we discard samples prior to convergence from the initial part of the chain, which is called the burn-in. All relevant data are within the paper and its Supporting Information files.

### Calculation of prevalence rate

To estimate the prevalence rate of Alzheimer’s disease in the Alpes-Maritimes region, a district in Southern France home to a population of one million population, we used the estimate of the total number of patients with Alzheimer’s disease as numerator and the total population count published by the French national institute of economic and statistical information (INSEE) [20] in 2010 as denominator (N = 1,078,729 in 2010). As our estimate included patients of all ages and most patients were older than 65 years, a subgroup analysis by age group and a prevalence rate estimate for the population above 65 years (N = 234,459) were carried out.

## Results

**Fig 1:**
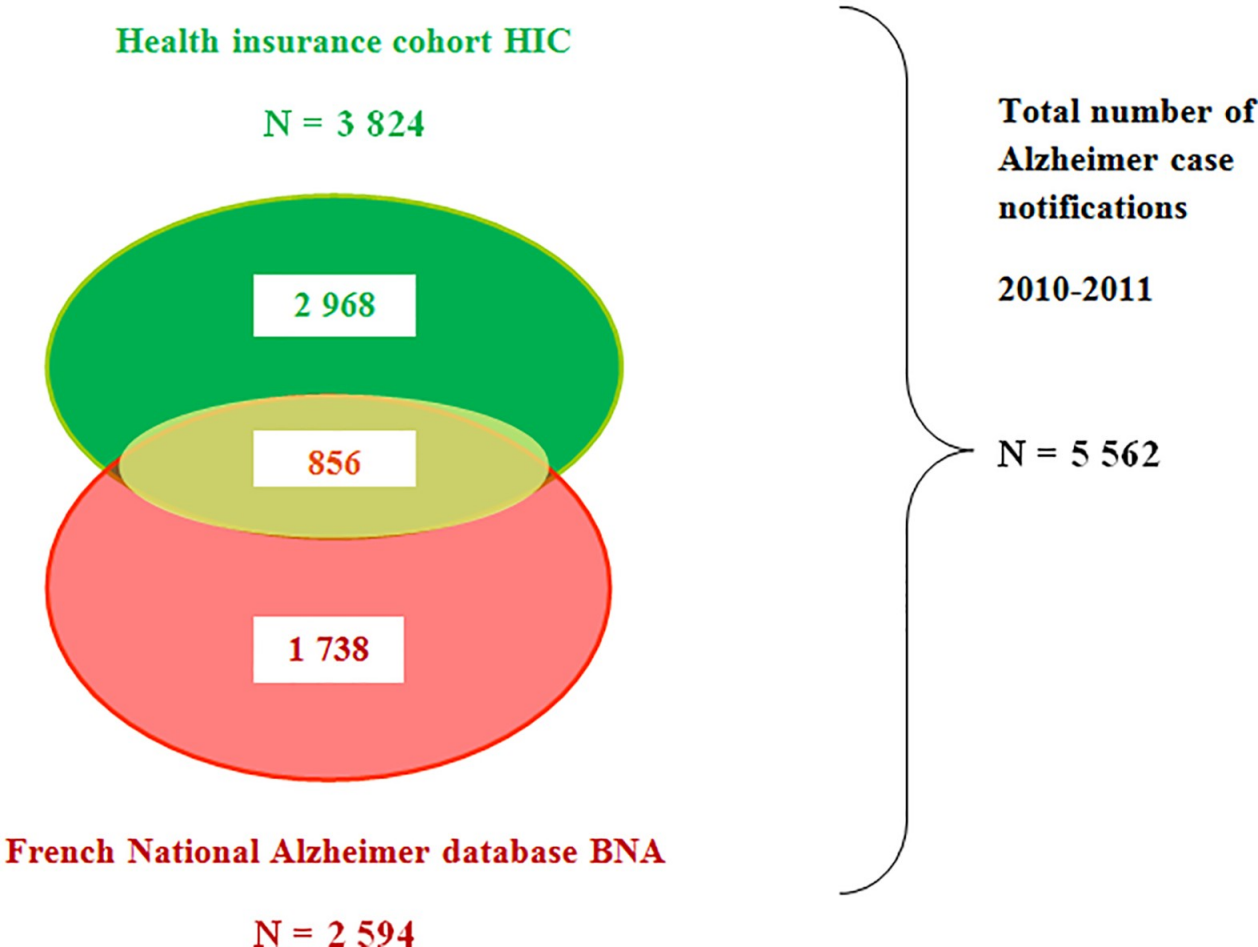
Total occurrence of Alzheimer’s disease cases collected by each surveillance system, Alpes-Maritimes, 2010–2011.

The total number of patients with Alzheimer’s disease or related conditions, obtained from the two databases, was 5,562 cases in 2010–2011. The health insurance cohort (HIC) collected 3,824 cases in this territory and the the French National Alzheimer database (BNA) 2,594 cases, 856 cases were common to the two databases, as presented in Fig 1. In the BNA, the mean age of patients with Alzheimer’s or related conditions was 82.2 years (standard deviation ± 7.4), and 71% were female, i.e. a F/M gender ratio of 2.41. These results are in line with those observed for the total copayment exemptions due to Alzheimer’s or related conditions notified in France in 2010 [21] (median age 82 years, sex-ratio F/M 2.7). In the health insurance cohort (HIC), the mean age of patients exempted from copayment due to Alzheimer’s disease was significantly older than in the BNA database (p<0.001), i.e. 86.1 years (standard deviation ± 4.5) and the F/M sex ratio was 3.2. Memory clinics (BNA) were visited by patients who were younger than those who were exempted from co-payment (HIC). Thus, as the age of subjects was not homogeneous between sources, taking age into account appeared particularly appropriate.

### Estimating the total number of patients with the Lincoln Petersen and Chapman estimator

As mentioned above there were 5,562 patients with the Alzheimer's disease detected by the health care system in the Alpes-Maritimes for the 2010–2011 period, of which 856 cases were common to the BNA and the Health Insurance databases, i.e. 15.4%. The distribution in a contingency table of all the reported cases, common or not to the two information systems, is presented in Table 1. It provides an initial estimate of the total number of cases by applying the Lincoln Petersen estimator. Under the assumption of independence of the information systems, highly probable given the absence of information transfer between the BNA and the health insurance information system, the Lincoln Petersen estimator provides an estimate of 11,588 individuals with Alzheimer's disease 95%CI[11,028–12,148] for the 2010–2011 period in the Alpes-Maritimes area. An estimate of the total number of cases, stratified by age and sex, was calculated to account for age-related heterogeneity of capture: this yielded 11,945 patients [95%CI: 9,562–14,328].

**Table 1:** Cases of Alzheimer’s disease collected by each independent information system.

		Health insurance cohort HIC		
		Yes	No	
**French National Alzheimer database**	Yes	856	1 738	2 594
	No	2 968	*6 026*	
		3 824		**11 588**

#### Lincoln Petersen estimator

Total number of cases N = (3824×2594)/856 = 11588 95*%CI* [11 028–12 148]

Standard deviation: 1.96×((3824×2594×2968×1738)/(856^3))^1/2 = 560

#### Unbiased Chapman estimator

Total number of cases *N* = $\frac{\left(3824+1)\times (2594+1\right)}{856}-1$ = 11 595 *95%CI* [11 036–12 153]

Standard deviation *SD* = $\sqrt{\frac{(3824+1)\times (2594+1)*2968*1738}{{\left(856+1\right)}^{2}(856+2)}}$ = 559

In 2010–2011, the estimate of the total number of Alzheimer’s disease cases, was of 11 588 and 11 595, in the Alpes-Maritimes following Lincoln Petersen and unbiased Chapman estimator respectively, leading to an estimated prevalence rate for the total population of 1.1%. Excluding patients younger than 65 years old, the estimate of the number of Alzheimer’s disease cases was 11 414 leading to an estimated prevalence rate of 4.9% for the population above 65 years old. Based on those values, the completeness of the health insurance cohort was 33.0%, and the completeness of the French National Alzheimer database was 22.4%, in 2010–2011.

### M_t_ ecological model

The M_t_ capture-recapture model was applied to the two sources with age (< 84 years, 85–89 years,> 90 years) and gender considered as covariates, i.e. male or female, aged less than 84 years old “captured or recaptured” by the French National Alzheimer database BNA and the Health Insurance Cohort database HIC, male or female aged 85 to 89 years old “captured or recaptured” by the BNA and the HIC databases, and so on. The complete model is presented in the appendix. Two Markov chains were used to obtain samples from the posterior distribution of the variable of interest. The average estimate was obtained following 25 000 iterations after discarding the initial 5 000 iterations. The Markov chains rapidly converged towards a stationary distribution, after 1 000 iterations. Finally, the estimate for an M_t_ model including age and gender as covariates yields 15,060 cases [median: 15,070; 95%HPDI: 14,490–15,630], leading to an estimated prevalence rate of 1.4% for the total population in 2010–2011. For the population above 65 years old, the estimate of the number of Alzheimer’s disease cases was 14,720, leading to an estimated prevalence rate of 6.3%. Based on those values, the completeness of the health insurance cohort was 25.4%, and the completeness of the French National Alzheimer database was 17.2%, in 2010–2011.

## Discussion

In this study, we were able to estimate the number of patients with Alzheimer's disease and related disorders in the Alpes-Maritimes. The application of the two-source Lincoln Petersen estimator provides an estimate of 11,588 patients, while the Bayesian estimation of the M_t_ ecological model yields an estimate of 15,060 patients. However, there is still a degree of uncertainty in the estimate due to the limitations of capture-recapture methods. As recommended [22], we confronted our results with available external data. Applying the prevalence rate per age group observed in the French PAQUID cohort [1] to the population of the Alpes-Maritimes area leads to an estimate of 13,686 patients with Alzheimer’s disease or related conditions, as detailed in the S2 Table. Thus, these estimations are in line with those obtained in our study but as there are certain limitations in each method, the actual disease rate lies within a range than in only one specific value. From our point of view, the progression of Alzheimer’s disease over several years is a key point in the catchability for each individual, as consultation in a memory clinic, copayment agreement and prescription of medications only eligible for Alzheimer’s disease, do not occur at the same time during the course of the disease. Therefore, a capture-recapture model that would not take time into consideration and specifically variables associated with time would undoubtedly yield a biased result. We believe that age and gender make homogeneous catchability for all patients of all ages unlikely through medical and administrative sources. Their integration as covariables yielded an estimate of 15,060 cases within a realistic interval. With this method, the estimated prevalence rate was 6.3% for the population above 65 years old in 2010–2011. As a comparison, during the same year in the United States, the estimated prevalence rate of Alzheimer’s disease was 10% among the U.S population above 65 years old, following the CHAP population-based longitudinal study [23]. There are some limitations in our prevalence rate estimate using the capture-recapture method. For example, the U.S national prevalence rate estimate was based on a protracted follow-up with projections taking incidence rate, risk factors, mortality and education level into account, whereas our regional capture-recapture study is only based on two databases and two years of follow-up. However, the estimate obtained with the M_t_ model using age and gender as covariates, gets closer to the U.S estimates in the population above 65 years than the estimate obtained with the Lincoln Petersen or the unbiased Chapman estimators, i.e. an estimated prevalence rate of 4.9%. Diagnostic criteria could also be another reason why our prevalence estimate may be lower than the one observed in the U.S population. A discrepancy due to differences in diagnostic criteria has been observed between the ADAMS [24] and the CHAP studies, two U.S population-based longitudinal studies estimating the prevalence rate of Alzheimer’s disease. The main reason was that vascular dementia and Alzheimer’s disease could not be considered at the same time for the same patient in the ADAMS study contrary to the CHAP study. In our case, vascular dementia was another diagnosis in memory clinics and had another code for copayment exemption. This difference in diagnostic criteria could be an explanation for our lower estimated prevalence rate.

The data from two epidemiological surveillance systems available in the Alpes-Maritimes territory were cross-referenced over a two-year period. In 2010–2011, their completeness was low as the health insurance cohort and the French National Alzheimer database accounted respectively for 25.4%, and 17.2% of all patients with Alzheimer’s disease in the Alpes-Maritimes. It appears that 5,562 patients with Alzheimer's disease were diagnosed and identified by the health system, i.e. a third of those affected by the disease in the Alpes-Maritimes department, according to our estimate. For the remainder, it is likely that they are either patients with loss of autonomy hosted in nursing homes, or with another serious condition such that Alzheimer's disease recedes in the background, or those with yet undiagnosed cognitive disorders.

We consider that determining the number of subjects affected by Alzheimer's disease provides essential information for the regional public health authority to implement a territorial and population-based approach. Such information is absolutely necessary to allocate available resources and adapt care to patient’s needs. Available data in France go back to the French population-based longitudinal dementia study PAQUID [1] in 2003, with patients aged over 75 years and living in a rural area. From our point of view, the usual projections based on the PAQUID French cohort rate by the regional public health authority cannot be considered sufficient to implement an efficient population-based health policy.

Our study only considered patients of all ages for whom the diagnosis of Alzheimer's disease was confirmed by a clinician. The next step will be to homogenize diagnostic strategies so as to achieve a more accurate description of the epidemiology of dementia and Alzheimer's disease in France. In order for a patient to be recorded by the health care system and to be exempt of copayments, he/she must be symptomatic, complain about the symptoms, and eventually get to see a doctor. Even after having consulted, dementia may not be suspected (lack of informant, inconclusive statements by the patient). The diagnosis is eventually made, and the case finally reported to the health insurance. Those reported cases may therefore legitimately be considered as mainly related to patients with advanced dementia with a significant impact on their daily life. Conversely, subjects whose diagnosis is made during memory clinic consultations are more likely to be diagnosed earlier in the course of the disease since they have taken the step of consulting a physician specializing in memory disorders. Several consultations were conducted to ensure the reality of diagnosis from clinical examinations, neuropsychological tests, such as MMSE or paraclinical examinations. A qualitative analysis of patients’ pathways collected by each of the two information systems can explain the relatively limited number of common subjects between the two databases. What has been observed in this study confirms that crossing available data on Alzheimer's disease is essential to achieve a more thorough description of this condition. Moreover, the ultimately limited number of common cases between the French National Alzheimer database and the health insurance cohort calls for careful analysis of each of the medico-administrative bases.

## Conclusion

In conclusion, the two information systems on Alzheimer's disease available for the Alpes-Maritimes territory were complementary. We consider this practical epidemiological example as particularly relevant as it illustrates the required approach for a capture-recapture procedure. From a public health perspective, the information collected from these different, independent sources points to the need for more concerted health policies, i.e. a shared vision of the health status of the population and the adoption of common goals. A deeper understanding of this condition thus appears as a prerequisite for adapting prevention, screening, clinical and medico-social care to patients’ needs. Determining the number of subjects affected by Alzheimer's disease is therefore the first step in a territorial and population-based approach to adequately allocate available resources. Our study shows that the number of subjects affected by Alzheimer's disease can be determined with reasonable accuracy within a French geographical unit, using available data from existing surveillance systems.

## Supporting information

S1 Table Estimating the total number of cases using the Lincoln-Petersen and the Unbiased Chapman estimators. (DOCX)

S2 Table Expected cases of Alzheimer’s disease in the Alpes-Maritimes population in 2010. (DOCX)
