# Supplementary material for: Alzheimer's disease: Estimating its prevalence rate in a French geographical unit using the National Alzheimer Data Bank and national health insurance information systems
Source: PLoS One. 2019 May 6;14(5):e0216221. doi: 10.1371/journal.pone.0216221 (PMC6502320; doi:10.1371/journal.pone.0216221)
Supplement: S1 Table — (DOCX) [file pone.0216221.s001.docx]

# Supporting information

## Estimating the total number of cases using the Lincoln-Petersen estimator.

By performing record-linkage between two databases A and B, cases are distributed in a 2-by-2 contingency table, N_A_ being the total number of cases in database A and N_B_ the total number of cases in database B.

**S1 Table. Estimating the total number of cases using the Lincoln-Petersen and the Unbiased Chapman estimators.**

|  | Present in database A | Absent from database A |  |
| --- | --- | --- | --- |
| Present in database B | Shared cases X_AB_ | Present in database B only X_B_ | Total cases from datasource B: N_B_ |
| Absent from database B | Present in database A only X_A_ | Absent from both databases X_X_ |  |
|  | Total cases from datasource A: N_A_ |  | Total number of cases N |

The number of shared cases is designated X_AB_, the number of cases present in database A only is X_A_ that in database B only is X_B_

The estimated number of cases absent from both databases and that of the total number of cases (N), assuming independence between the two reporting systems, can be calculated as follows:

Lincoln Petersen estimator:

$Var(N) = \frac{\left( N_{A}+1 \right)\times\left( N_{B}+1 \right)X_{A}X_{B}}{\left( X_{AB}+1 \right)^{2}(X_{AB}+2)}$

95 %CI(N)= N ± 1,96Var(N)

Unbiased Chapman estimator:

$N = \frac{\left( N_{A}+1 \right) \times\left( N_{B}+1 \right)}{X_{AB}}-1$ $Var(N) = \frac{\left( N_{A}+1 \right)\times\left( N_{B}+1 \right)X_{A}X_{B}}{\left( X_{AB}+1 \right)^{2}(X_{AB}+2)}$

These estimators allow calculating the completeness of each data source. Limitations of this capture-recapture method, which relies on only two data sources, rest in the lack of a possibility to assess the dependence between the two information sources.

## Capture-recapture ecological model

We applied M_t_ model and Bayesian population estimation to our sample of patients with Alzheimer’s disease, i.e. model implying that capture probability changes only with time of capture (M_t_).

Let p_iτ_ denote the capture probability for individual i = 1, 2, 3 … N at time τ = 1, 2, 3... T.

F(i) represents the first time that individual i is observed. Therefore p_iτ_ = P which is the initial capture probability of i for times τ =1,…, F(i), and the recapture probabilities for τ = F(i) + 1, …T assuming F(i) < T. Thus individual i is captured at time τ =1, not captured at time τ = F(i) – 1 and captured at time τ = F(i), and can be recaptured between times τ = F(i) + 1 and τ, with total capture times = T.

The saturated M_t_ model integrating time (t) has capture probabilities of the form:

Logit (p_iτ_) = µ + α_τ_

In this model the estimated parameters are µ (mean capture rate expressed as logit), α_τ_ (time effect for capture). It is assumed that capture probabilities are independent, given the parameter values.

Let θ = {µ, α, N} with α = {α_1_,… ,α_T_}. The vector x, which describes the capture history of all individuals, is given by:

$f(x\left| \theta)\propto\frac{N!}{\left( N-n \right)!}\prod_{t=1}^{T} .\prod_{i=1}^{N} .p_{it}^{x_{it}}{(1- p_{it})}^{(1- x_{it})} \right.$

where n (n<N) denotes the total number of individuals captured in the study, i.e. n unique individuals captured initially. The vector x_it_ = 0 if individual i is not captured at time t and x_it_ = 1 if the individual is captured at time t, i.e. x=(x_11_, …, x_1T_ , … x_21_, …, x_2T_ , x_N1_, …, x_NT_) where x_it_ = 0 or x_it_ =1.

### WinBUGS code

model{

phi ~ dunif(0,1)

mu ~ dnorm( 0.0,100)

sigma2alpha ~ dgamma(3,4)

for (k in 1:12) {

alpha[k]~ dnorm( 0.0,sigma2alpha)

}

for(i in 1:M){

for (k in 1:12) {

z[i,k]~dbin(phi,1)

logit(p[i,k]) <- mu + alpha[k]

zp[i,k]<-p[i,k]*z[i,k]

x[i,k] ~ dbin(zp[i,k],T)

}

}

N<-sum(z[,])

}

list(M=1400,T=12,x=structure(.Data=c(

#H HIC -84

1, 1, 1, 1, 1, 1, 1, 1, 1, 1, 1, 1, 1, 1, 1, 1, 1, 1, 1, 1,

1, 1, 1, 1, 1, 1, 1, 1, 1, 1, 1, 1, 1, 1, 1, 1, 1, 1, 1, 1,

1, 1, 1, 1, 1, 1, 1, 1, 1, 1, 1, 1, 1, 1, 1, 1, 1, 1, 1, 1,

1, 1, 1, 1, 1, 1, 1, 1, 1, 1, 1, 1, 1, 1, 1, 1, 1, 1, 1, 1,

1, 1, 1, 1, 1, 1, 1, 1, 1, 1, 1, 1, 1, 1, 1, 1, 1, 1, 1, 1,

1, 1, 1, 1, 1, 1, 1, 1, 1, 1, 1, 1, 1, 1, 1, 1, 1, 1, 1, 1,

1, 1, 1, 1, 1, 1, 1, 1, 1, 1, 1, 1, 1, 1, 1, 1, 1, 1, 1, 1,

1, 1, 1, 1, 1, 1, 1, 1, 1, 1, 1, 1, 1, 1, 1, 1, 1, 1, 1, 1,

1, 1, 1, 1, 1, 1, 1, 1, 1, 1, 1, 1, 1, 1, 1, 1, 1, 1, 1, 1,

1, 1, 1, 1, 1, 1, 1, 1, 1, 1, 1, 1, 1, 1, 1, 1, 1, 1, 1, 1,

1, 1, 1, 1, 1, 1, 1, 1, 1, 1, 1, 1, 1, 1, 1, 1, 1, 1, 1, 1,

1, 1, 1, 1, 1, 1, 1, 1, 1, 1, 1, 1, 1, 1, 1, 1, 1, 1, 1, 1,

1, 1, 1, 1, 1, 1, 1, 1, 1, 1, 1, 1, 1, 1, 1, 1, 1, 1, 1, 1,

1, 1, 1, 1, 1, 1, 1, 1, 1, 1, 1, 1, 1, 1, 1, 1, 1, 1, 1, 1,

1, 1, 1, 1, 1, 1, 1, 1, 1, 1, 1, 1, 1, 1, 1, 1, 1, 1, 1, 1,

1, 1, 1, 1, 1, 1, 1, 1, 1, 1, 1, 1, 1, 1, 1, 1, 1, 1, 1, 1,

1, 1, 1, 1, 1, 1, 1, 1, 1, 1, 1, 1, 1, 1, 1, 1, 1, 1, 1, 1,

1, 1, 1, 1, 1, 1, 1, 1, 1, 1, 1, 1, 1, 1, 1, 1, 1, 1, 1, 1,

1, 1, 1, 1, 1, 1, 1, 1, 1, 1, 1, 1, 1, 1, 1, 1, 1, 1, 1, 1,

1, 1, 1, 1, 1, 1, 1, 1, 1, 1, 1, 1, 1, 1, 1, 1, 1, 1, 1, 1,

1, 1, 1, 1, 1, 1, 1, 1, 1, 1, 1, 1, 1, 1, 1, 1, 1, 1, 1, 1,

1, 1, 1, 1, 1, 1, 1, 1, 1, 1, 1, 1, 1, 1, 1, 1, 1, 2, 2, 2,

2, 2, 2, 2, 2, 2, 2, 2, 2, 2, 2, 2, 2, 2, 2, 2, 2, 2, 2, 2,

2, 2, 2, 2, 2, 2, 2, 2, 2, 2, 2, 2, 2, 2, 2, 2, 2, 2, 2, 2,

2, 2, 2, 2, 2, 2, 2, 2, 2, 2, 2, 2, 2, 2, 2, 2, 2, 2, 2, 2,

2, 2, 2, 2, 2, 2, 2, 2, 2, 2, 2, 2, 2, 2, 2, 2, 2, 2, 2, 2,

2, 2, 2, 2, 2, 2, 2, 2, 2, 2, 2, 2, 2, 2, 2, 2, 2, 2, 2, 2,

2, 2, 2, 2, 2, 2, 2, 2, 2, 2, 2, 2, 2, 2, 2, 2, 2, 2, 2, 2,

2, 2, 2, 2, 2, 2, 2, 2, 2, 2, 2, 2, 2, 2, 2, 2, 2, 2, 2, 2,

2, 2, 2, 2, 2, 2, 2, 2, 2, 2, 2, 2, 2, 2, 2, 2, 2, 2, 2, 2,

2, 2, 2, 2, 2, 2, 2, 2, 2, 2, 2, 2, 2, 2, 2, 2, 2, 2, 2, 2,

2, 2, 2, 2, 2, 0, 0, 0, 0, 0, 0, 0, 0, 0, 0, 0, 0, 0, 0, 0,

0, 0, 0, 0, 0, 0, 0, 0, 0, 0, 0, 0, 0, 0, 0, 0, 0, 0, 0, 0,

0, 0, 0, 0, 0, 0, 0, 0, 0, 0, 0, 0, 0, 0, 0, 0, 0, 0, 0, 0,

0, 0, 0, 0, 0, 0, 0, 0, 0, 0, 0, 0, 0, 0, 0, 0, 0, 0, 0, 0,

0, 0, 0, 0, 0, 0, 0, 0, 0, 0, 0, 0, 0, 0, 0, 0, 0, 0, 0, 0,

0, 0, 0, 0, 0, 0, 0, 0, 0, 0, 0, 0, 0, 0, 0, 0, 0, 0, 0, 0,

0, 0, 0, 0, 0, 0, 0, 0, 0, 0, 0, 0, 0, 0, 0, 0, 0, 0, 0, 0,

0, 0, 0, 0, 0, 0, 0, 0, 0, 0, 0, 0, 0, 0, 0, 0, 0, 0, 0, 0,

0, 0, 0, 0, 0, 0, 0, 0, 0, 0, 0, 0, 0, 0, 0, 0, 0, 0, 0, 0,

0, 0, 0, 0, 0, 0, 0, 0, 0, 0, 0, 0, 0, 0, 0, 0, 0, 0, 0, 0,

0, 0, 0, 0, 0, 0, 0, 0, 0, 0, 0, 0, 0, 0, 0, 0, 0, 0, 0, 0,

0, 0, 0, 0, 0, 0, 0, 0, 0, 0, 0, 0, 0, 0, 0, 0, 0, 0, 0, 0,

0, 0, 0, 0, 0, 0, 0, 0, 0, 0, 0, 0, 0, 0, 0, 0, 0, 0, 0, 0,

0, 0, 0, 0, 0, 0, 0, 0, 0, 0, 0, 0, 0, 0, 0, 0, 0, 0, 0, 0,

0, 0, 0, 0, 0, 0, 0, 0, 0, 0, 0, 0, 0, 0, 0, 0, 0, 0, 0, 0,

0, 0, 0, 0, 0, 0, 0, 0, 0, 0, 0, 0, 0, 0, 0, 0, 0, 0, 0, 0,

0, 0, 0, 0, 0, 0, 0, 0, 0, 0, 0, 0, 0, 0, 0, 0, 0, 0, 0, 0,

0, 0, 0, 0, 0, 0, 0, 0, 0, 0, 0, 0, 0, 0, 0, 0, 0, 0, 0, 0,

0, 0, 0, 0, 0, 0, 0, 0, 0, 0, 0, 0, 0, 0, 0, 0, 0, 0, 0, 0,

0, 0, 0, 0, 0, 0, 0, 0, 0, 0, 0, 0, 0, 0, 0, 0, 0, 0, 0, 0,

0, 0, 0, 0, 0, 0, 0, 0, 0, 0, 0, 0, 0, 0, 0, 0, 0, 0, 0, 0,

0, 0, 0, 0, 0, 0, 0, 0, 0, 0, 0, 0, 0, 0, 0, 0, 0, 0, 0, 0,

0, 0, 0, 0, 0, 0, 0, 0, 0, 0, 0, 0, 0, 0, 0, 0, 0, 0, 0, 0,

0, 0, 0, 0, 0, 0, 0, 0, 0, 0, 0, 0, 0, 0, 0, 0, 0, 0, 0, 0,

0, 0, 0, 0, 0, 0, 0, 0, 0, 0, 0, 0, 0, 0, 0, 0, 0, 0, 0, 0,

0, 0, 0, 0, 0, 0, 0, 0, 0, 0, 0, 0, 0, 0, 0, 0, 0, 0, 0, 0,

0, 0, 0, 0, 0, 0, 0, 0, 0, 0, 0, 0, 0, 0, 0, 0, 0, 0, 0, 0,

0, 0, 0, 0, 0, 0, 0, 0, 0, 0, 0, 0, 0, 0, 0, 0, 0, 0, 0, 0,

0, 0, 0, 0, 0, 0, 0, 0, 0, 0, 0, 0, 0, 0, 0, 0, 0, 0, 0, 0,

0, 0, 0, 0, 0, 0, 0, 0, 0, 0, 0, 0, 0, 0, 0, 0, 0, 0, 0, 0,

0, 0, 0, 0, 0, 0, 0, 0, 0, 0, 0, 0, 0, 0, 0, 0, 0, 0, 0, 0,

0, 0, 0, 0, 0, 0, 0, 0, 0, 0, 0, 0, 0, 0, 0, 0, 0, 0, 0, 0,

0, 0, 0, 0, 0, 0, 0, 0, 0, 0, 0, 0, 0, 0, 0, 0, 0, 0, 0, 0,

0, 0, 0, 0, 0, 0, 0, 0, 0, 0, 0, 0, 0, 0, 0, 0, 0, 0, 0, 0,

0, 0, 0, 0, 0, 0, 0, 0, 0, 0, 0, 0, 0, 0, 0, 0, 0, 0, 0, 0,

0, 0, 0, 0, 0, 0, 0, 0, 0, 0, 0, 0, 0, 0, 0, 0, 0, 0, 0, 0,

0, 0, 0, 0, 0, 0, 0, 0, 0, 0, 0, 0, 0, 0, 0, 0, 0, 0, 0, 0,

0, 0, 0, 0, 0, 0, 0, 0, 0, 0, 0, 0, 0, 0, 0, 0, 0, 0, 0, 0,

0, 0, 0, 0, 0, 0, 0, 0, 0, 0, 0, 0, 0, 0, 0, 0, 0, 0, 0, 0,

#H BNA84-

1, 1, 1, 1, 1, 1, 1, 1, 1, 1, 1, 1, 1, 1, 1, 1, 1, 1, 1, 1,

1, 1, 1, 1, 1, 1, 1, 1, 1, 1, 1, 1, 1, 1, 1, 1, 1, 1, 1, 1,

1, 1, 1, 1, 1, 1, 1, 1, 1, 1, 1, 1, 1, 1, 1, 1, 1, 1, 1, 1,

1, 1, 1, 1, 1, 1, 1, 1, 1, 1, 1, 1, 1, 1, 1, 1, 1, 1, 1, 1,

1, 1, 1, 1, 1, 1, 1, 1, 1, 1, 1, 1, 1, 1, 1, 1, 1, 1, 1, 1,

1, 1, 1, 1, 1, 1, 1, 1, 1, 1, 1, 1, 1, 1, 1, 1, 1, 1, 1, 1,

1, 1, 1, 1, 1, 1, 1, 1, 1, 1, 1, 1, 1, 1, 1, 1, 1, 1, 1, 1,

1, 1, 1, 1, 1, 1, 1, 1, 1, 1, 1, 1, 1, 1, 1, 1, 1, 1, 1, 1,

1, 1, 1, 1, 1, 1, 1, 1, 1, 1, 1, 1, 1, 1, 1, 1, 1, 1, 1, 1,

1, 1, 1, 1, 1, 1, 1, 1, 1, 1, 1, 1, 1, 1, 1, 1, 1, 1, 1, 1,

1, 1, 1, 1, 1, 1, 1, 1, 1, 1, 1, 1, 1, 1, 1, 1, 1, 1, 1, 1,

1, 1, 1, 1, 1, 1, 1, 1, 1, 1, 1, 1, 1, 1, 1, 1, 1, 1, 1, 1,

1, 1, 1, 1, 1, 1, 1, 1, 1, 1, 1, 1, 1, 1, 1, 1, 1, 1, 1, 1,

1, 1, 1, 1, 1, 1, 1, 1, 1, 1, 1, 1, 1, 1, 1, 1, 1, 1, 1, 1,

1, 1, 1, 1, 1, 1, 1, 1, 1, 1, 1, 1, 1, 1, 1, 1, 1, 1, 1, 1,

1, 1, 1, 1, 1, 1, 1, 1, 2, 2, 2, 2, 2, 2, 2, 2, 2, 2, 2, 2,

2, 2, 2, 2, 2, 2, 2, 2, 2, 2, 2, 2, 2, 2, 2, 2, 2, 2, 2, 2,

2, 2, 2, 2, 2, 2, 2, 2, 2, 2, 2, 2, 2, 2, 2, 2, 2, 2, 2, 2,

2, 2, 2, 2, 2, 2, 2, 2, 2, 2, 2, 2, 2, 2, 2, 2, 2, 2, 2, 2,

2, 2, 2, 2, 2, 2, 2, 2, 2, 2, 2, 2, 2, 2, 2, 2, 2, 2, 2, 2,

2, 2, 2, 2, 2, 2, 2, 2, 2, 2, 2, 2, 2, 2, 2, 2, 2, 2, 2, 2,

2, 2, 2, 2, 2, 2, 2, 2, 2, 2, 2, 2, 2, 2, 2, 2, 2, 2, 2, 2,

2, 2, 2, 2, 2, 2, 2, 2, 2, 2, 2, 2, 2, 2, 2, 2, 2, 2, 2, 2,

2, 2, 2, 2, 2, 2, 2, 2, 2, 2, 2, 2, 2, 2, 2, 2, 2, 2, 2, 2,

2, 2, 2, 2, 2, 2, 2, 2, 2, 2, 2, 2, 2, 2, 2, 2, 0, 0, 0, 0,

0, 0, 0, 0, 0, 0, 0, 0, 0, 0, 0, 0, 0, 0, 0, 0, 0, 0, 0, 0,

0, 0, 0, 0, 0, 0, 0, 0, 0, 0, 0, 0, 0, 0, 0, 0, 0, 0, 0, 0,

0, 0, 0, 0, 0, 0, 0, 0, 0, 0, 0, 0, 0, 0, 0, 0, 0, 0, 0, 0,

0, 0, 0, 0, 0, 0, 0, 0, 0, 0, 0, 0, 0, 0, 0, 0, 0, 0, 0, 0,

0, 0, 0, 0, 0, 0, 0, 0, 0, 0, 0, 0, 0, 0, 0, 0, 0, 0, 0, 0,

0, 0, 0, 0, 0, 0, 0, 0, 0, 0, 0, 0, 0, 0, 0, 0, 0, 0, 0, 0,

0, 0, 0, 0, 0, 0, 0, 0, 0, 0, 0, 0, 0, 0, 0, 0, 0, 0, 0, 0,

0, 0, 0, 0, 0, 0, 0, 0, 0, 0, 0, 0, 0, 0, 0, 0, 0, 0, 0, 0,

0, 0, 0, 0, 0, 0, 0, 0, 0, 0, 0, 0, 0, 0, 0, 0, 0, 0, 0, 0,

0, 0, 0, 0, 0, 0, 0, 0, 0, 0, 0, 0, 0, 0, 0, 0, 0, 0, 0, 0,

0, 0, 0, 0, 0, 0, 0, 0, 0, 0, 0, 0, 0, 0, 0, 0, 0, 0, 0, 0,

0, 0, 0, 0, 0, 0, 0, 0, 0, 0, 0, 0, 0, 0, 0, 0, 0, 0, 0, 0,

0, 0, 0, 0, 0, 0, 0, 0, 0, 0, 0, 0, 0, 0, 0, 0, 0, 0, 0, 0,

0, 0, 0, 0, 0, 0, 0, 0, 0, 0, 0, 0, 0, 0, 0, 0, 0, 0, 0, 0,

0, 0, 0, 0, 0, 0, 0, 0, 0, 0, 0, 0, 0, 0, 0, 0, 0, 0, 0, 0,

0, 0, 0, 0, 0, 0, 0, 0, 0, 0, 0, 0, 0, 0, 0, 0, 0, 0, 0, 0,

0, 0, 0, 0, 0, 0, 0, 0, 0, 0, 0, 0, 0, 0, 0, 0, 0, 0, 0, 0,

0, 0, 0, 0, 0, 0, 0, 0, 0, 0, 0, 0, 0, 0, 0, 0, 0, 0, 0, 0,

0, 0, 0, 0, 0, 0, 0, 0, 0, 0, 0, 0, 0, 0, 0, 0, 0, 0, 0, 0,

0, 0, 0, 0, 0, 0, 0, 0, 0, 0, 0, 0, 0, 0, 0, 0, 0, 0, 0, 0,

0, 0, 0, 0, 0, 0, 0, 0, 0, 0, 0, 0, 0, 0, 0, 0, 0, 0, 0, 0,

0, 0, 0, 0, 0, 0, 0, 0, 0, 0, 0, 0, 0, 0, 0, 0, 0, 0, 0, 0,

0, 0, 0, 0, 0, 0, 0, 0, 0, 0, 0, 0, 0, 0, 0, 0, 0, 0, 0, 0,

0, 0, 0, 0, 0, 0, 0, 0, 0, 0, 0, 0, 0, 0, 0, 0, 0, 0, 0, 0,

0, 0, 0, 0, 0, 0, 0, 0, 0, 0, 0, 0, 0, 0, 0, 0, 0, 0, 0, 0,

0, 0, 0, 0, 0, 0, 0, 0, 0, 0, 0, 0, 0, 0, 0, 0, 0, 0, 0, 0,

0, 0, 0, 0, 0, 0, 0, 0, 0, 0, 0, 0, 0, 0, 0, 0, 0, 0, 0, 0,

0, 0, 0, 0, 0, 0, 0, 0, 0, 0, 0, 0, 0, 0, 0, 0, 0, 0, 0, 0,

0, 0, 0, 0, 0, 0, 0, 0, 0, 0, 0, 0, 0, 0, 0, 0, 0, 0, 0, 0,

0, 0, 0, 0, 0, 0, 0, 0, 0, 0, 0, 0, 0, 0, 0, 0, 0, 0, 0, 0,

0, 0, 0, 0, 0, 0, 0, 0, 0, 0, 0, 0, 0, 0, 0, 0, 0, 0, 0, 0,

0, 0, 0, 0, 0, 0, 0, 0, 0, 0, 0, 0, 0, 0, 0, 0, 0, 0, 0, 0,

0, 0, 0, 0, 0, 0, 0, 0, 0, 0, 0, 0, 0, 0, 0, 0, 0, 0, 0, 0,

0, 0, 0, 0, 0, 0, 0, 0, 0, 0, 0, 0, 0, 0, 0, 0, 0, 0, 0, 0,

0, 0, 0, 0, 0, 0, 0, 0, 0, 0, 0, 0, 0, 0, 0, 0, 0, 0, 0, 0,

0, 0, 0, 0, 0, 0, 0, 0, 0, 0, 0, 0, 0, 0, 0, 0, 0, 0, 0, 0,

0, 0, 0, 0, 0, 0, 0, 0, 0, 0, 0, 0, 0, 0, 0, 0, 0, 0, 0, 0,

0, 0, 0, 0, 0, 0, 0, 0, 0, 0, 0, 0, 0, 0, 0, 0, 0, 0, 0, 0,

0, 0, 0, 0, 0, 0, 0, 0, 0, 0, 0, 0, 0, 0, 0, 0, 0, 0, 0, 0,

0, 0, 0, 0, 0, 0, 0, 0, 0, 0, 0, 0, 0, 0, 0, 0, 0, 0, 0, 0,

0, 0, 0, 0, 0, 0, 0, 0, 0, 0, 0, 0, 0, 0, 0, 0, 0, 0, 0, 0,

0, 0, 0, 0, 0, 0, 0, 0, 0, 0, 0, 0, 0, 0, 0, 0, 0, 0, 0, 0,

0, 0, 0, 0, 0, 0, 0, 0, 0, 0, 0, 0, 0, 0, 0, 0, 0, 0, 0, 0,

0, 0, 0, 0, 0, 0, 0, 0, 0, 0, 0, 0, 0, 0, 0, 0, 0, 0, 0, 0,

0, 0, 0, 0, 0, 0, 0, 0, 0, 0, 0, 0, 0, 0, 0, 0, 0, 0, 0, 0,

#H HIC85-89

1, 1, 1, 1, 1, 1, 1, 1, 1, 1, 1, 1, 1, 1, 1, 1, 1, 1, 1, 1,

1, 1, 1, 1, 1, 1, 1, 1, 1, 1, 1, 1, 1, 1, 1, 1, 1, 1, 1, 1,

1, 1, 1, 1, 1, 1, 1, 1, 1, 1, 1, 1, 1, 1, 1, 1, 1, 1, 1, 1,

1, 1, 1, 1, 1, 1, 1, 1, 1, 1, 1, 1, 1, 1, 1, 1, 1, 1, 1, 1,

1, 1, 1, 1, 1, 1, 1, 1, 1, 1, 1, 1, 1, 1, 1, 1, 1, 1, 1, 1,

1, 1, 1, 1, 1, 1, 1, 1, 1, 1, 1, 1, 1, 1, 1, 1, 1, 1, 1, 1,

1, 1, 1, 1, 1, 1, 1, 1, 1, 1, 1, 1, 1, 1, 1, 1, 1, 1, 1, 1,

1, 1, 1, 1, 1, 1, 1, 1, 1, 1, 1, 1, 1, 1, 1, 1, 1, 1, 1, 1,

1, 1, 1, 1, 1, 1, 1, 1, 1, 1, 1, 1, 1, 1, 1, 1, 1, 2, 2, 2,

2, 2, 2, 2, 2, 2, 2, 2, 2, 2, 2, 2, 2, 2, 2, 2, 2, 2, 2, 2,

2, 2, 2, 2, 2, 2, 2, 2, 2, 2, 2, 2, 2, 2, 2, 2, 2, 2, 2, 2,

2, 2, 2, 2, 2, 2, 2, 2, 2, 0, 0, 0, 0, 0, 0, 0, 0, 0, 0, 0,

0, 0, 0, 0, 0, 0, 0, 0, 0, 0, 0, 0, 0, 0, 0, 0, 0, 0, 0, 0,

0, 0, 0, 0, 0, 0, 0, 0, 0, 0, 0, 0, 0, 0, 0, 0, 0, 0, 0, 0,

0, 0, 0, 0, 0, 0, 0, 0, 0, 0, 0, 0, 0, 0, 0, 0, 0, 0, 0, 0,

0, 0, 0, 0, 0, 0, 0, 0, 0, 0, 0, 0, 0, 0, 0, 0, 0, 0, 0, 0,

0, 0, 0, 0, 0, 0, 0, 0, 0, 0, 0, 0, 0, 0, 0, 0, 0, 0, 0, 0,

0, 0, 0, 0, 0, 0, 0, 0, 0, 0, 0, 0, 0, 0, 0, 0, 0, 0, 0, 0,

0, 0, 0, 0, 0, 0, 0, 0, 0, 0, 0, 0, 0, 0, 0, 0, 0, 0, 0, 0,

0, 0, 0, 0, 0, 0, 0, 0, 0, 0, 0, 0, 0, 0, 0, 0, 0, 0, 0, 0,

0, 0, 0, 0, 0, 0, 0, 0, 0, 0, 0, 0, 0, 0, 0, 0, 0, 0, 0, 0,

0, 0, 0, 0, 0, 0, 0, 0, 0, 0, 0, 0, 0, 0, 0, 0, 0, 0, 0, 0,

0, 0, 0, 0, 0, 0, 0, 0, 0, 0, 0, 0, 0, 0, 0, 0, 0, 0, 0, 0,

0, 0, 0, 0, 0, 0, 0, 0, 0, 0, 0, 0, 0, 0, 0, 0, 0, 0, 0, 0,

0, 0, 0, 0, 0, 0, 0, 0, 0, 0, 0, 0, 0, 0, 0, 0, 0, 0, 0, 0,

0, 0, 0, 0, 0, 0, 0, 0, 0, 0, 0, 0, 0, 0, 0, 0, 0, 0, 0, 0,

0, 0, 0, 0, 0, 0, 0, 0, 0, 0, 0, 0, 0, 0, 0, 0, 0, 0, 0, 0,

0, 0, 0, 0, 0, 0, 0, 0, 0, 0, 0, 0, 0, 0, 0, 0, 0, 0, 0, 0,

0, 0, 0, 0, 0, 0, 0, 0, 0, 0, 0, 0, 0, 0, 0, 0, 0, 0, 0, 0,

0, 0, 0, 0, 0, 0, 0, 0, 0, 0, 0, 0, 0, 0, 0, 0, 0, 0, 0, 0,

0, 0, 0, 0, 0, 0, 0, 0, 0, 0, 0, 0, 0, 0, 0, 0, 0, 0, 0, 0,

0, 0, 0, 0, 0, 0, 0, 0, 0, 0, 0, 0, 0, 0, 0, 0, 0, 0, 0, 0,

0, 0, 0, 0, 0, 0, 0, 0, 0, 0, 0, 0, 0, 0, 0, 0, 0, 0, 0, 0,

0, 0, 0, 0, 0, 0, 0, 0, 0, 0, 0, 0, 0, 0, 0, 0, 0, 0, 0, 0,

0, 0, 0, 0, 0, 0, 0, 0, 0, 0, 0, 0, 0, 0, 0, 0, 0, 0, 0, 0,

0, 0, 0, 0, 0, 0, 0, 0, 0, 0, 0, 0, 0, 0, 0, 0, 0, 0, 0, 0,

0, 0, 0, 0, 0, 0, 0, 0, 0, 0, 0, 0, 0, 0, 0, 0, 0, 0, 0, 0,

0, 0, 0, 0, 0, 0, 0, 0, 0, 0, 0, 0, 0, 0, 0, 0, 0, 0, 0, 0,

0, 0, 0, 0, 0, 0, 0, 0, 0, 0, 0, 0, 0, 0, 0, 0, 0, 0, 0, 0,

0, 0, 0, 0, 0, 0, 0, 0, 0, 0, 0, 0, 0, 0, 0, 0, 0, 0, 0, 0,

0, 0, 0, 0, 0, 0, 0, 0, 0, 0, 0, 0, 0, 0, 0, 0, 0, 0, 0, 0,

0, 0, 0, 0, 0, 0, 0, 0, 0, 0, 0, 0, 0, 0, 0, 0, 0, 0, 0, 0,

0, 0, 0, 0, 0, 0, 0, 0, 0, 0, 0, 0, 0, 0, 0, 0, 0, 0, 0, 0,

0, 0, 0, 0, 0, 0, 0, 0, 0, 0, 0, 0, 0, 0, 0, 0, 0, 0, 0, 0,

0, 0, 0, 0, 0, 0, 0, 0, 0, 0, 0, 0, 0, 0, 0, 0, 0, 0, 0, 0,

0, 0, 0, 0, 0, 0, 0, 0, 0, 0, 0, 0, 0, 0, 0, 0, 0, 0, 0, 0,

0, 0, 0, 0, 0, 0, 0, 0, 0, 0, 0, 0, 0, 0, 0, 0, 0, 0, 0, 0,

0, 0, 0, 0, 0, 0, 0, 0, 0, 0, 0, 0, 0, 0, 0, 0, 0, 0, 0, 0,

0, 0, 0, 0, 0, 0, 0, 0, 0, 0, 0, 0, 0, 0, 0, 0, 0, 0, 0, 0,

0, 0, 0, 0, 0, 0, 0, 0, 0, 0, 0, 0, 0, 0, 0, 0, 0, 0, 0, 0,

0, 0, 0, 0, 0, 0, 0, 0, 0, 0, 0, 0, 0, 0, 0, 0, 0, 0, 0, 0,

0, 0, 0, 0, 0, 0, 0, 0, 0, 0, 0, 0, 0, 0, 0, 0, 0, 0, 0, 0,

0, 0, 0, 0, 0, 0, 0, 0, 0, 0, 0, 0, 0, 0, 0, 0, 0, 0, 0, 0,

0, 0, 0, 0, 0, 0, 0, 0, 0, 0, 0, 0, 0, 0, 0, 0, 0, 0, 0, 0,

0, 0, 0, 0, 0, 0, 0, 0, 0, 0, 0, 0, 0, 0, 0, 0, 0, 0, 0, 0,

0, 0, 0, 0, 0, 0, 0, 0, 0, 0, 0, 0, 0, 0, 0, 0, 0, 0, 0, 0,

0, 0, 0, 0, 0, 0, 0, 0, 0, 0, 0, 0, 0, 0, 0, 0, 0, 0, 0, 0,

0, 0, 0, 0, 0, 0, 0, 0, 0, 0, 0, 0, 0, 0, 0, 0, 0, 0, 0, 0,

0, 0, 0, 0, 0, 0, 0, 0, 0, 0, 0, 0, 0, 0, 0, 0, 0, 0, 0, 0,

0, 0, 0, 0, 0, 0, 0, 0, 0, 0, 0, 0, 0, 0, 0, 0, 0, 0, 0, 0,

0, 0, 0, 0, 0, 0, 0, 0, 0, 0, 0, 0, 0, 0, 0, 0, 0, 0, 0, 0,

0, 0, 0, 0, 0, 0, 0, 0, 0, 0, 0, 0, 0, 0, 0, 0, 0, 0, 0, 0,

0, 0, 0, 0, 0, 0, 0, 0, 0, 0, 0, 0, 0, 0, 0, 0, 0, 0, 0, 0,

0, 0, 0, 0, 0, 0, 0, 0, 0, 0, 0, 0, 0, 0, 0, 0, 0, 0, 0, 0,

0, 0, 0, 0, 0, 0, 0, 0, 0, 0, 0, 0, 0, 0, 0, 0, 0, 0, 0, 0,

0, 0, 0, 0, 0, 0, 0, 0, 0, 0, 0, 0, 0, 0, 0, 0, 0, 0, 0, 0,

0, 0, 0, 0, 0, 0, 0, 0, 0, 0, 0, 0, 0, 0, 0, 0, 0, 0, 0, 0,

0, 0, 0, 0, 0, 0, 0, 0, 0, 0, 0, 0, 0, 0, 0, 0, 0, 0, 0, 0,

0, 0, 0, 0, 0, 0, 0, 0, 0, 0, 0, 0, 0, 0, 0, 0, 0, 0, 0, 0,

0, 0, 0, 0, 0, 0, 0, 0, 0, 0, 0, 0, 0, 0, 0, 0, 0, 0, 0, 0,

#H BNA85-90

1, 1, 1, 1, 1, 1, 1, 1, 1, 1, 1, 1, 1, 1, 1, 1, 1, 1, 1, 1,

1, 1, 1, 1, 1, 1, 1, 1, 1, 1, 1, 1, 1, 1, 1, 1, 1, 1, 1, 1,

1, 1, 1, 1, 1, 1, 1, 1, 1, 1, 1, 1, 1, 1, 1, 1, 1, 1, 1, 1,

1, 1, 1, 1, 1, 1, 1, 1, 1, 1, 1, 1, 1, 1, 1, 1, 1, 1, 1, 1,

1, 1, 1, 1, 1, 1, 1, 1, 1, 1, 1, 1, 1, 1, 1, 1, 1, 1, 1, 1,

1, 1, 1, 1, 1, 1, 1, 1, 1, 1, 1, 1, 1, 1, 1, 1, 1, 1, 1, 1,

1, 2, 2, 2, 2, 2, 2, 2, 2, 2, 2, 2, 2, 2, 2, 2, 2, 2, 2, 2,

2, 2, 2, 2, 2, 2, 2, 2, 2, 2, 2, 2, 2, 2, 2, 2, 2, 2, 2, 2,

2, 2, 2, 2, 2, 2, 2, 2, 2, 2, 2, 2, 2, 0, 0, 0, 0, 0, 0, 0,

0, 0, 0, 0, 0, 0, 0, 0, 0, 0, 0, 0, 0, 0, 0, 0, 0, 0, 0, 0,

0, 0, 0, 0, 0, 0, 0, 0, 0, 0, 0, 0, 0, 0, 0, 0, 0, 0, 0, 0,

0, 0, 0, 0, 0, 0, 0, 0, 0, 0, 0, 0, 0, 0, 0, 0, 0, 0, 0, 0,

0, 0, 0, 0, 0, 0, 0, 0, 0, 0, 0, 0, 0, 0, 0, 0, 0, 0, 0, 0,

0, 0, 0, 0, 0, 0, 0, 0, 0, 0, 0, 0, 0, 0, 0, 0, 0, 0, 0, 0,

0, 0, 0, 0, 0, 0, 0, 0, 0, 0, 0, 0, 0, 0, 0, 0, 0, 0, 0, 0,

0, 0, 0, 0, 0, 0, 0, 0, 0, 0, 0, 0, 0, 0, 0, 0, 0, 0, 0, 0,

0, 0, 0, 0, 0, 0, 0, 0, 0, 0, 0, 0, 0, 0, 0, 0, 0, 0, 0, 0,

0, 0, 0, 0, 0, 0, 0, 0, 0, 0, 0, 0, 0, 0, 0, 0, 0, 0, 0, 0,

0, 0, 0, 0, 0, 0, 0, 0, 0, 0, 0, 0, 0, 0, 0, 0, 0, 0, 0, 0,

0, 0, 0, 0, 0, 0, 0, 0, 0, 0, 0, 0, 0, 0, 0, 0, 0, 0, 0, 0,

0, 0, 0, 0, 0, 0, 0, 0, 0, 0, 0, 0, 0, 0, 0, 0, 0, 0, 0, 0,

0, 0, 0, 0, 0, 0, 0, 0, 0, 0, 0, 0, 0, 0, 0, 0, 0, 0, 0, 0,

0, 0, 0, 0, 0, 0, 0, 0, 0, 0, 0, 0, 0, 0, 0, 0, 0, 0, 0, 0,

0, 0, 0, 0, 0, 0, 0, 0, 0, 0, 0, 0, 0, 0, 0, 0, 0, 0, 0, 0,

0, 0, 0, 0, 0, 0, 0, 0, 0, 0, 0, 0, 0, 0, 0, 0, 0, 0, 0, 0,

0, 0, 0, 0, 0, 0, 0, 0, 0, 0, 0, 0, 0, 0, 0, 0, 0, 0, 0, 0,

0, 0, 0, 0, 0, 0, 0, 0, 0, 0, 0, 0, 0, 0, 0, 0, 0, 0, 0, 0,

0, 0, 0, 0, 0, 0, 0, 0, 0, 0, 0, 0, 0, 0, 0, 0, 0, 0, 0, 0,

0, 0, 0, 0, 0, 0, 0, 0, 0, 0, 0, 0, 0, 0, 0, 0, 0, 0, 0, 0,

0, 0, 0, 0, 0, 0, 0, 0, 0, 0, 0, 0, 0, 0, 0, 0, 0, 0, 0, 0,

0, 0, 0, 0, 0, 0, 0, 0, 0, 0, 0, 0, 0, 0, 0, 0, 0, 0, 0, 0,

0, 0, 0, 0, 0, 0, 0, 0, 0, 0, 0, 0, 0, 0, 0, 0, 0, 0, 0, 0,

0, 0, 0, 0, 0, 0, 0, 0, 0, 0, 0, 0, 0, 0, 0, 0, 0, 0, 0, 0,

0, 0, 0, 0, 0, 0, 0, 0, 0, 0, 0, 0, 0, 0, 0, 0, 0, 0, 0, 0,

0, 0, 0, 0, 0, 0, 0, 0, 0, 0, 0, 0, 0, 0, 0, 0, 0, 0, 0, 0,

0, 0, 0, 0, 0, 0, 0, 0, 0, 0, 0, 0, 0, 0, 0, 0, 0, 0, 0, 0,

0, 0, 0, 0, 0, 0, 0, 0, 0, 0, 0, 0, 0, 0, 0, 0, 0, 0, 0, 0,

0, 0, 0, 0, 0, 0, 0, 0, 0, 0, 0, 0, 0, 0, 0, 0, 0, 0, 0, 0,

0, 0, 0, 0, 0, 0, 0, 0, 0, 0, 0, 0, 0, 0, 0, 0, 0, 0, 0, 0,

0, 0, 0, 0, 0, 0, 0, 0, 0, 0, 0, 0, 0, 0, 0, 0, 0, 0, 0, 0,

0, 0, 0, 0, 0, 0, 0, 0, 0, 0, 0, 0, 0, 0, 0, 0, 0, 0, 0, 0,

0, 0, 0, 0, 0, 0, 0, 0, 0, 0, 0, 0, 0, 0, 0, 0, 0, 0, 0, 0,

0, 0, 0, 0, 0, 0, 0, 0, 0, 0, 0, 0, 0, 0, 0, 0, 0, 0, 0, 0,

0, 0, 0, 0, 0, 0, 0, 0, 0, 0, 0, 0, 0, 0, 0, 0, 0, 0, 0, 0,

0, 0, 0, 0, 0, 0, 0, 0, 0, 0, 0, 0, 0, 0, 0, 0, 0, 0, 0, 0,

0, 0, 0, 0, 0, 0, 0, 0, 0, 0, 0, 0, 0, 0, 0, 0, 0, 0, 0, 0,

0, 0, 0, 0, 0, 0, 0, 0, 0, 0, 0, 0, 0, 0, 0, 0, 0, 0, 0, 0,

0, 0, 0, 0, 0, 0, 0, 0, 0, 0, 0, 0, 0, 0, 0, 0, 0, 0, 0, 0,

0, 0, 0, 0, 0, 0, 0, 0, 0, 0, 0, 0, 0, 0, 0, 0, 0, 0, 0, 0,

0, 0, 0, 0, 0, 0, 0, 0, 0, 0, 0, 0, 0, 0, 0, 0, 0, 0, 0, 0,

0, 0, 0, 0, 0, 0, 0, 0, 0, 0, 0, 0, 0, 0, 0, 0, 0, 0, 0, 0,

0, 0, 0, 0, 0, 0, 0, 0, 0, 0, 0, 0, 0, 0, 0, 0, 0, 0, 0, 0,

0, 0, 0, 0, 0, 0, 0, 0, 0, 0, 0, 0, 0, 0, 0, 0, 0, 0, 0, 0,

0, 0, 0, 0, 0, 0, 0, 0, 0, 0, 0, 0, 0, 0, 0, 0, 0, 0, 0, 0,

0, 0, 0, 0, 0, 0, 0, 0, 0, 0, 0, 0, 0, 0, 0, 0, 0, 0, 0, 0,

0, 0, 0, 0, 0, 0, 0, 0, 0, 0, 0, 0, 0, 0, 0, 0, 0, 0, 0, 0,

0, 0, 0, 0, 0, 0, 0, 0, 0, 0, 0, 0, 0, 0, 0, 0, 0, 0, 0, 0,

0, 0, 0, 0, 0, 0, 0, 0, 0, 0, 0, 0, 0, 0, 0, 0, 0, 0, 0, 0,

0, 0, 0, 0, 0, 0, 0, 0, 0, 0, 0, 0, 0, 0, 0, 0, 0, 0, 0, 0,

0, 0, 0, 0, 0, 0, 0, 0, 0, 0, 0, 0, 0, 0, 0, 0, 0, 0, 0, 0,

0, 0, 0, 0, 0, 0, 0, 0, 0, 0, 0, 0, 0, 0, 0, 0, 0, 0, 0, 0,

0, 0, 0, 0, 0, 0, 0, 0, 0, 0, 0, 0, 0, 0, 0, 0, 0, 0, 0, 0,

0, 0, 0, 0, 0, 0, 0, 0, 0, 0, 0, 0, 0, 0, 0, 0, 0, 0, 0, 0,

0, 0, 0, 0, 0, 0, 0, 0, 0, 0, 0, 0, 0, 0, 0, 0, 0, 0, 0, 0,

0, 0, 0, 0, 0, 0, 0, 0, 0, 0, 0, 0, 0, 0, 0, 0, 0, 0, 0, 0,

0, 0, 0, 0, 0, 0, 0, 0, 0, 0, 0, 0, 0, 0, 0, 0, 0, 0, 0, 0,

0, 0, 0, 0, 0, 0, 0, 0, 0, 0, 0, 0, 0, 0, 0, 0, 0, 0, 0, 0,

0, 0, 0, 0, 0, 0, 0, 0, 0, 0, 0, 0, 0, 0, 0, 0, 0, 0, 0, 0,

0, 0, 0, 0, 0, 0, 0, 0, 0, 0, 0, 0, 0, 0, 0, 0, 0, 0, 0, 0,

0, 0, 0, 0, 0, 0, 0, 0, 0, 0, 0, 0, 0, 0, 0, 0, 0, 0, 0, 0,

#H HIC 90+

1, 1, 1, 1, 1, 1, 1, 1, 1, 1, 1, 1, 1, 1, 1, 1, 1, 1, 1, 1,

1, 1, 1, 1, 1, 1, 1, 1, 1, 1, 1, 1, 1, 1, 1, 1, 1, 1, 1, 1,

1, 1, 1, 1, 1, 1, 1, 1, 1, 1, 1, 1, 1, 1, 1, 1, 1, 1, 1, 1,

1, 1, 1, 1, 1, 1, 1, 1, 1, 1, 1, 1, 1, 1, 1, 1, 1, 1, 1, 1,

2, 2, 2, 2, 2, 2, 2, 2, 2, 2, 2, 2, 0, 0, 0, 0, 0, 0, 0, 0,

0, 0, 0, 0, 0, 0, 0, 0, 0, 0, 0, 0, 0, 0, 0, 0, 0, 0, 0, 0,

0, 0, 0, 0, 0, 0, 0, 0, 0, 0, 0, 0, 0, 0, 0, 0, 0, 0, 0, 0,

0, 0, 0, 0, 0, 0, 0, 0, 0, 0, 0, 0, 0, 0, 0, 0, 0, 0, 0, 0,

0, 0, 0, 0, 0, 0, 0, 0, 0, 0, 0, 0, 0, 0, 0, 0, 0, 0, 0, 0,

0, 0, 0, 0, 0, 0, 0, 0, 0, 0, 0, 0, 0, 0, 0, 0, 0, 0, 0, 0,

0, 0, 0, 0, 0, 0, 0, 0, 0, 0, 0, 0, 0, 0, 0, 0, 0, 0, 0, 0,

0, 0, 0, 0, 0, 0, 0, 0, 0, 0, 0, 0, 0, 0, 0, 0, 0, 0, 0, 0,

0, 0, 0, 0, 0, 0, 0, 0, 0, 0, 0, 0, 0, 0, 0, 0, 0, 0, 0, 0,

0, 0, 0, 0, 0, 0, 0, 0, 0, 0, 0, 0, 0, 0, 0, 0, 0, 0, 0, 0,

0, 0, 0, 0, 0, 0, 0, 0, 0, 0, 0, 0, 0, 0, 0, 0, 0, 0, 0, 0,

0, 0, 0, 0, 0, 0, 0, 0, 0, 0, 0, 0, 0, 0, 0, 0, 0, 0, 0, 0,

0, 0, 0, 0, 0, 0, 0, 0, 0, 0, 0, 0, 0, 0, 0, 0, 0, 0, 0, 0,

0, 0, 0, 0, 0, 0, 0, 0, 0, 0, 0, 0, 0, 0, 0, 0, 0, 0, 0, 0,

0, 0, 0, 0, 0, 0, 0, 0, 0, 0, 0, 0, 0, 0, 0, 0, 0, 0, 0, 0,

0, 0, 0, 0, 0, 0, 0, 0, 0, 0, 0, 0, 0, 0, 0, 0, 0, 0, 0, 0,

0, 0, 0, 0, 0, 0, 0, 0, 0, 0, 0, 0, 0, 0, 0, 0, 0, 0, 0, 0,

0, 0, 0, 0, 0, 0, 0, 0, 0, 0, 0, 0, 0, 0, 0, 0, 0, 0, 0, 0,

0, 0, 0, 0, 0, 0, 0, 0, 0, 0, 0, 0, 0, 0, 0, 0, 0, 0, 0, 0,

0, 0, 0, 0, 0, 0, 0, 0, 0, 0, 0, 0, 0, 0, 0, 0, 0, 0, 0, 0,

0, 0, 0, 0, 0, 0, 0, 0, 0, 0, 0, 0, 0, 0, 0, 0, 0, 0, 0, 0,

0, 0, 0, 0, 0, 0, 0, 0, 0, 0, 0, 0, 0, 0, 0, 0, 0, 0, 0, 0,

0, 0, 0, 0, 0, 0, 0, 0, 0, 0, 0, 0, 0, 0, 0, 0, 0, 0, 0, 0,

0, 0, 0, 0, 0, 0, 0, 0, 0, 0, 0, 0, 0, 0, 0, 0, 0, 0, 0, 0,

0, 0, 0, 0, 0, 0, 0, 0, 0, 0, 0, 0, 0, 0, 0, 0, 0, 0, 0, 0,

0, 0, 0, 0, 0, 0, 0, 0, 0, 0, 0, 0, 0, 0, 0, 0, 0, 0, 0, 0,

0, 0, 0, 0, 0, 0, 0, 0, 0, 0, 0, 0, 0, 0, 0, 0, 0, 0, 0, 0,

0, 0, 0, 0, 0, 0, 0, 0, 0, 0, 0, 0, 0, 0, 0, 0, 0, 0, 0, 0,

0, 0, 0, 0, 0, 0, 0, 0, 0, 0, 0, 0, 0, 0, 0, 0, 0, 0, 0, 0,

0, 0, 0, 0, 0, 0, 0, 0, 0, 0, 0, 0, 0, 0, 0, 0, 0, 0, 0, 0,

0, 0, 0, 0, 0, 0, 0, 0, 0, 0, 0, 0, 0, 0, 0, 0, 0, 0, 0, 0,

0, 0, 0, 0, 0, 0, 0, 0, 0, 0, 0, 0, 0, 0, 0, 0, 0, 0, 0, 0,

0, 0, 0, 0, 0, 0, 0, 0, 0, 0, 0, 0, 0, 0, 0, 0, 0, 0, 0, 0,

0, 0, 0, 0, 0, 0, 0, 0, 0, 0, 0, 0, 0, 0, 0, 0, 0, 0, 0, 0,

0, 0, 0, 0, 0, 0, 0, 0, 0, 0, 0, 0, 0, 0, 0, 0, 0, 0, 0, 0,

0, 0, 0, 0, 0, 0, 0, 0, 0, 0, 0, 0, 0, 0, 0, 0, 0, 0, 0, 0,

0, 0, 0, 0, 0, 0, 0, 0, 0, 0, 0, 0, 0, 0, 0, 0, 0, 0, 0, 0,

0, 0, 0, 0, 0, 0, 0, 0, 0, 0, 0, 0, 0, 0, 0, 0, 0, 0, 0, 0,

0, 0, 0, 0, 0, 0, 0, 0, 0, 0, 0, 0, 0, 0, 0, 0, 0, 0, 0, 0,

0, 0, 0, 0, 0, 0, 0, 0, 0, 0, 0, 0, 0, 0, 0, 0, 0, 0, 0, 0,

0, 0, 0, 0, 0, 0, 0, 0, 0, 0, 0, 0, 0, 0, 0, 0, 0, 0, 0, 0,

0, 0, 0, 0, 0, 0, 0, 0, 0, 0, 0, 0, 0, 0, 0, 0, 0, 0, 0, 0,

0, 0, 0, 0, 0, 0, 0, 0, 0, 0, 0, 0, 0, 0, 0, 0, 0, 0, 0, 0,

0, 0, 0, 0, 0, 0, 0, 0, 0, 0, 0, 0, 0, 0, 0, 0, 0, 0, 0, 0,

0, 0, 0, 0, 0, 0, 0, 0, 0, 0, 0, 0, 0, 0, 0, 0, 0, 0, 0, 0,

0, 0, 0, 0, 0, 0, 0, 0, 0, 0, 0, 0, 0, 0, 0, 0, 0, 0, 0, 0,

0, 0, 0, 0, 0, 0, 0, 0, 0, 0, 0, 0, 0, 0, 0, 0, 0, 0, 0, 0,

0, 0, 0, 0, 0, 0, 0, 0, 0, 0, 0, 0, 0, 0, 0, 0, 0, 0, 0, 0,

0, 0, 0, 0, 0, 0, 0, 0, 0, 0, 0, 0, 0, 0, 0, 0, 0, 0, 0, 0,

0, 0, 0, 0, 0, 0, 0, 0, 0, 0, 0, 0, 0, 0, 0, 0, 0, 0, 0, 0,

0, 0, 0, 0, 0, 0, 0, 0, 0, 0, 0, 0, 0, 0, 0, 0, 0, 0, 0, 0,

0, 0, 0, 0, 0, 0, 0, 0, 0, 0, 0, 0, 0, 0, 0, 0, 0, 0, 0, 0,

0, 0, 0, 0, 0, 0, 0, 0, 0, 0, 0, 0, 0, 0, 0, 0, 0, 0, 0, 0,

0, 0, 0, 0, 0, 0, 0, 0, 0, 0, 0, 0, 0, 0, 0, 0, 0, 0, 0, 0,

0, 0, 0, 0, 0, 0, 0, 0, 0, 0, 0, 0, 0, 0, 0, 0, 0, 0, 0, 0,

0, 0, 0, 0, 0, 0, 0, 0, 0, 0, 0, 0, 0, 0, 0, 0, 0, 0, 0, 0,

0, 0, 0, 0, 0, 0, 0, 0, 0, 0, 0, 0, 0, 0, 0, 0, 0, 0, 0, 0,

0, 0, 0, 0, 0, 0, 0, 0, 0, 0, 0, 0, 0, 0, 0, 0, 0, 0, 0, 0,

0, 0, 0, 0, 0, 0, 0, 0, 0, 0, 0, 0, 0, 0, 0, 0, 0, 0, 0, 0,

0, 0, 0, 0, 0, 0, 0, 0, 0, 0, 0, 0, 0, 0, 0, 0, 0, 0, 0, 0,

0, 0, 0, 0, 0, 0, 0, 0, 0, 0, 0, 0, 0, 0, 0, 0, 0, 0, 0, 0,

0, 0, 0, 0, 0, 0, 0, 0, 0, 0, 0, 0, 0, 0, 0, 0, 0, 0, 0, 0,

0, 0, 0, 0, 0, 0, 0, 0, 0, 0, 0, 0, 0, 0, 0, 0, 0, 0, 0, 0,

0, 0, 0, 0, 0, 0, 0, 0, 0, 0, 0, 0, 0, 0, 0, 0, 0, 0, 0, 0,

0, 0, 0, 0, 0, 0, 0, 0, 0, 0, 0, 0, 0, 0, 0, 0, 0, 0, 0, 0,

0, 0, 0, 0, 0, 0, 0, 0, 0, 0, 0, 0, 0, 0, 0, 0, 0, 0, 0, 0,

#H BNA90+

1, 1, 1, 1, 1, 1, 1, 1, 1, 1, 1, 1, 1, 1, 1, 1, 1, 1, 1, 1,

1, 1, 1, 1, 1, 1, 1, 1, 1, 1, 1, 1, 1, 1, 1, 1, 1, 1, 1, 1,

1, 1, 1, 1, 1, 1, 1, 1, 1, 1, 1, 1, 1, 1, 1, 1, 1, 1, 1, 1,

1, 1, 2, 2, 2, 2, 2, 2, 2, 2, 2, 2, 2, 2, 0, 0, 0, 0, 0, 0,

0, 0, 0, 0, 0, 0, 0, 0, 0, 0, 0, 0, 0, 0, 0, 0, 0, 0, 0, 0,

0, 0, 0, 0, 0, 0, 0, 0, 0, 0, 0, 0, 0, 0, 0, 0, 0, 0, 0, 0,

0, 0, 0, 0, 0, 0, 0, 0, 0, 0, 0, 0, 0, 0, 0, 0, 0, 0, 0, 0,

0, 0, 0, 0, 0, 0, 0, 0, 0, 0, 0, 0, 0, 0, 0, 0, 0, 0, 0, 0,

0, 0, 0, 0, 0, 0, 0, 0, 0, 0, 0, 0, 0, 0, 0, 0, 0, 0, 0, 0,

0, 0, 0, 0, 0, 0, 0, 0, 0, 0, 0, 0, 0, 0, 0, 0, 0, 0, 0, 0,

0, 0, 0, 0, 0, 0, 0, 0, 0, 0, 0, 0, 0, 0, 0, 0, 0, 0, 0, 0,

0, 0, 0, 0, 0, 0, 0, 0, 0, 0, 0, 0, 0, 0, 0, 0, 0, 0, 0, 0,

0, 0, 0, 0, 0, 0, 0, 0, 0, 0, 0, 0, 0, 0, 0, 0, 0, 0, 0, 0,

0, 0, 0, 0, 0, 0, 0, 0, 0, 0, 0, 0, 0, 0, 0, 0, 0, 0, 0, 0,

0, 0, 0, 0, 0, 0, 0, 0, 0, 0, 0, 0, 0, 0, 0, 0, 0, 0, 0, 0,

0, 0, 0, 0, 0, 0, 0, 0, 0, 0, 0, 0, 0, 0, 0, 0, 0, 0, 0, 0,

0, 0, 0, 0, 0, 0, 0, 0, 0, 0, 0, 0, 0, 0, 0, 0, 0, 0, 0, 0,

0, 0, 0, 0, 0, 0, 0, 0, 0, 0, 0, 0, 0, 0, 0, 0, 0, 0, 0, 0,

0, 0, 0, 0, 0, 0, 0, 0, 0, 0, 0, 0, 0, 0, 0, 0, 0, 0, 0, 0,

0, 0, 0, 0, 0, 0, 0, 0, 0, 0, 0, 0, 0, 0, 0, 0, 0, 0, 0, 0,

0, 0, 0, 0, 0, 0, 0, 0, 0, 0, 0, 0, 0, 0, 0, 0, 0, 0, 0, 0,

0, 0, 0, 0, 0, 0, 0, 0, 0, 0, 0, 0, 0, 0, 0, 0, 0, 0, 0, 0,

0, 0, 0, 0, 0, 0, 0, 0, 0, 0, 0, 0, 0, 0, 0, 0, 0, 0, 0, 0,

0, 0, 0, 0, 0, 0, 0, 0, 0, 0, 0, 0, 0, 0, 0, 0, 0, 0, 0, 0,

0, 0, 0, 0, 0, 0, 0, 0, 0, 0, 0, 0, 0, 0, 0, 0, 0, 0, 0, 0,

0, 0, 0, 0, 0, 0, 0, 0, 0, 0, 0, 0, 0, 0, 0, 0, 0, 0, 0, 0,

0, 0, 0, 0, 0, 0, 0, 0, 0, 0, 0, 0, 0, 0, 0, 0, 0, 0, 0, 0,

0, 0, 0, 0, 0, 0, 0, 0, 0, 0, 0, 0, 0, 0, 0, 0, 0, 0, 0, 0,

0, 0, 0, 0, 0, 0, 0, 0, 0, 0, 0, 0, 0, 0, 0, 0, 0, 0, 0, 0,

0, 0, 0, 0, 0, 0, 0, 0, 0, 0, 0, 0, 0, 0, 0, 0, 0, 0, 0, 0,

0, 0, 0, 0, 0, 0, 0, 0, 0, 0, 0, 0, 0, 0, 0, 0, 0, 0, 0, 0,

0, 0, 0, 0, 0, 0, 0, 0, 0, 0, 0, 0, 0, 0, 0, 0, 0, 0, 0, 0,

0, 0, 0, 0, 0, 0, 0, 0, 0, 0, 0, 0, 0, 0, 0, 0, 0, 0, 0, 0,

0, 0, 0, 0, 0, 0, 0, 0, 0, 0, 0, 0, 0, 0, 0, 0, 0, 0, 0, 0,

0, 0, 0, 0, 0, 0, 0, 0, 0, 0, 0, 0, 0, 0, 0, 0, 0, 0, 0, 0,

0, 0, 0, 0, 0, 0, 0, 0, 0, 0, 0, 0, 0, 0, 0, 0, 0, 0, 0, 0,

0, 0, 0, 0, 0, 0, 0, 0, 0, 0, 0, 0, 0, 0, 0, 0, 0, 0, 0, 0,

0, 0, 0, 0, 0, 0, 0, 0, 0, 0, 0, 0, 0, 0, 0, 0, 0, 0, 0, 0,

0, 0, 0, 0, 0, 0, 0, 0, 0, 0, 0, 0, 0, 0, 0, 0, 0, 0, 0, 0,

0, 0, 0, 0, 0, 0, 0, 0, 0, 0, 0, 0, 0, 0, 0, 0, 0, 0, 0, 0,

0, 0, 0, 0, 0, 0, 0, 0, 0, 0, 0, 0, 0, 0, 0, 0, 0, 0, 0, 0,

0, 0, 0, 0, 0, 0, 0, 0, 0, 0, 0, 0, 0, 0, 0, 0, 0, 0, 0, 0,

0, 0, 0, 0, 0, 0, 0, 0, 0, 0, 0, 0, 0, 0, 0, 0, 0, 0, 0, 0,

0, 0, 0, 0, 0, 0, 0, 0, 0, 0, 0, 0, 0, 0, 0, 0, 0, 0, 0, 0,

0, 0, 0, 0, 0, 0, 0, 0, 0, 0, 0, 0, 0, 0, 0, 0, 0, 0, 0, 0,

0, 0, 0, 0, 0, 0, 0, 0, 0, 0, 0, 0, 0, 0, 0, 0, 0, 0, 0, 0,

0, 0, 0, 0, 0, 0, 0, 0, 0, 0, 0, 0, 0, 0, 0, 0, 0, 0, 0, 0,

0, 0, 0, 0, 0, 0, 0, 0, 0, 0, 0, 0, 0, 0, 0, 0, 0, 0, 0, 0,

0, 0, 0, 0, 0, 0, 0, 0, 0, 0, 0, 0, 0, 0, 0, 0, 0, 0, 0, 0,

0, 0, 0, 0, 0, 0, 0, 0, 0, 0, 0, 0, 0, 0, 0, 0, 0, 0, 0, 0,

0, 0, 0, 0, 0, 0, 0, 0, 0, 0, 0, 0, 0, 0, 0, 0, 0, 0, 0, 0,

0, 0, 0, 0, 0, 0, 0, 0, 0, 0, 0, 0, 0, 0, 0, 0, 0, 0, 0, 0,

0, 0, 0, 0, 0, 0, 0, 0, 0, 0, 0, 0, 0, 0, 0, 0, 0, 0, 0, 0,

0, 0, 0, 0, 0, 0, 0, 0, 0, 0, 0, 0, 0, 0, 0, 0, 0, 0, 0, 0,

0, 0, 0, 0, 0, 0, 0, 0, 0, 0, 0, 0, 0, 0, 0, 0, 0, 0, 0, 0,

0, 0, 0, 0, 0, 0, 0, 0, 0, 0, 0, 0, 0, 0, 0, 0, 0, 0, 0, 0,

0, 0, 0, 0, 0, 0, 0, 0, 0, 0, 0, 0, 0, 0, 0, 0, 0, 0, 0, 0,

0, 0, 0, 0, 0, 0, 0, 0, 0, 0, 0, 0, 0, 0, 0, 0, 0, 0, 0, 0,

0, 0, 0, 0, 0, 0, 0, 0, 0, 0, 0, 0, 0, 0, 0, 0, 0, 0, 0, 0,

0, 0, 0, 0, 0, 0, 0, 0, 0, 0, 0, 0, 0, 0, 0, 0, 0, 0, 0, 0,

0, 0, 0, 0, 0, 0, 0, 0, 0, 0, 0, 0, 0, 0, 0, 0, 0, 0, 0, 0,

0, 0, 0, 0, 0, 0, 0, 0, 0, 0, 0, 0, 0, 0, 0, 0, 0, 0, 0, 0,

0, 0, 0, 0, 0, 0, 0, 0, 0, 0, 0, 0, 0, 0, 0, 0, 0, 0, 0, 0,

0, 0, 0, 0, 0, 0, 0, 0, 0, 0, 0, 0, 0, 0, 0, 0, 0, 0, 0, 0,

0, 0, 0, 0, 0, 0, 0, 0, 0, 0, 0, 0, 0, 0, 0, 0, 0, 0, 0, 0,

0, 0, 0, 0, 0, 0, 0, 0, 0, 0, 0, 0, 0, 0, 0, 0, 0, 0, 0, 0,

0, 0, 0, 0, 0, 0, 0, 0, 0, 0, 0, 0, 0, 0, 0, 0, 0, 0, 0, 0,

0, 0, 0, 0, 0, 0, 0, 0, 0, 0, 0, 0, 0, 0, 0, 0, 0, 0, 0, 0,

0, 0, 0, 0, 0, 0, 0, 0, 0, 0, 0, 0, 0, 0, 0, 0, 0, 0, 0, 0,

0, 0, 0, 0, 0, 0, 0, 0, 0, 0, 0, 0, 0, 0, 0, 0, 0, 0, 0, 0,

#F HIC -84

1, 1, 1, 1, 1, 1, 1, 1, 1, 1, 1, 1, 1, 1, 1, 1, 1, 1, 1, 1,

1, 1, 1, 1, 1, 1, 1, 1, 1, 1, 1, 1, 1, 1, 1, 1, 1, 1, 1, 1,

1, 1, 1, 1, 1, 1, 1, 1, 1, 1, 1, 1, 1, 1, 1, 1, 1, 1, 1, 1,

1, 1, 1, 1, 1, 1, 1, 1, 1, 1, 1, 1, 1, 1, 1, 1, 1, 1, 1, 1,

1, 1, 1, 1, 1, 1, 1, 1, 1, 1, 1, 1, 1, 1, 1, 1, 1, 1, 1, 1,

1, 1, 1, 1, 1, 1, 1, 1, 1, 1, 1, 1, 1, 1, 1, 1, 1, 1, 1, 1,

1, 1, 1, 1, 1, 1, 1, 1, 1, 1, 1, 1, 1, 1, 1, 1, 1, 1, 1, 1,

1, 1, 1, 1, 1, 1, 1, 1, 1, 1, 1, 1, 1, 1, 1, 1, 1, 1, 1, 1,

1, 1, 1, 1, 1, 1, 1, 1, 1, 1, 1, 1, 1, 1, 1, 1, 1, 1, 1, 1,

1, 1, 1, 1, 1, 1, 1, 1, 1, 1, 1, 1, 1, 1, 1, 1, 1, 1, 1, 1,

1, 1, 1, 1, 1, 1, 1, 1, 1, 1, 1, 1, 1, 1, 1, 1, 1, 1, 1, 1,

1, 1, 1, 1, 1, 1, 1, 1, 1, 1, 1, 1, 1, 1, 1, 1, 1, 1, 1, 1,

1, 1, 1, 1, 1, 1, 1, 1, 1, 1, 1, 1, 1, 1, 1, 1, 1, 1, 1, 1,

1, 1, 1, 1, 1, 1, 1, 1, 1, 1, 1, 1, 1, 1, 1, 1, 1, 1, 1, 1,

1, 1, 1, 1, 1, 1, 1, 1, 1, 1, 1, 1, 1, 1, 1, 1, 1, 1, 1, 1,

1, 1, 1, 1, 1, 1, 1, 1, 1, 1, 1, 1, 1, 1, 1, 1, 1, 1, 1, 1,

1, 1, 1, 1, 1, 1, 1, 1, 1, 1, 1, 1, 1, 1, 1, 1, 1, 1, 1, 1,

1, 1, 1, 1, 1, 1, 1, 1, 1, 1, 1, 1, 1, 1, 1, 1, 1, 1, 1, 1,

1, 1, 1, 1, 1, 1, 1, 1, 1, 1, 1, 1, 1, 1, 1, 1, 1, 1, 1, 1,

1, 1, 1, 1, 1, 1, 1, 1, 1, 1, 1, 1, 1, 1, 1, 1, 1, 1, 1, 1,

1, 1, 1, 1, 1, 1, 1, 1, 1, 1, 1, 1, 1, 1, 1, 1, 1, 1, 1, 1,

1, 1, 1, 1, 1, 1, 1, 1, 1, 1, 1, 1, 1, 1, 1, 1, 1, 1, 1, 1,

1, 1, 1, 1, 1, 1, 1, 1, 1, 1, 1, 1, 1, 1, 1, 1, 1, 1, 1, 1,

1, 1, 1, 1, 1, 1, 1, 1, 1, 1, 1, 1, 1, 1, 1, 1, 1, 1, 1, 1,

1, 1, 1, 1, 1, 1, 1, 1, 1, 1, 1, 1, 1, 1, 1, 1, 1, 1, 1, 1,

1, 1, 1, 1, 1, 1, 1, 1, 1, 1, 1, 1, 1, 1, 1, 1, 1, 1, 1, 1,

1, 1, 1, 1, 1, 1, 1, 1, 1, 1, 1, 1, 1, 1, 1, 1, 1, 1, 1, 1,

1, 1, 1, 1, 1, 1, 1, 1, 1, 1, 1, 1, 1, 1, 1, 1, 1, 1, 1, 1,

1, 1, 1, 1, 1, 1, 1, 1, 1, 1, 1, 1, 1, 1, 1, 1, 1, 1, 1, 1,

1, 1, 1, 1, 1, 1, 1, 1, 1, 1, 1, 1, 1, 1, 1, 1, 1, 1, 1, 1,

1, 1, 1, 1, 1, 1, 1, 1, 1, 1, 1, 1, 1, 1, 1, 1, 1, 1, 1, 1,

1, 1, 1, 1, 1, 1, 1, 1, 1, 1, 1, 1, 1, 1, 1, 1, 1, 1, 1, 1,

1, 1, 1, 1, 1, 1, 1, 1, 1, 1, 1, 1, 1, 1, 1, 1, 1, 1, 1, 1,

1, 1, 1, 1, 1, 1, 1, 1, 1, 1, 1, 1, 1, 1, 1, 1, 1, 1, 1, 1,

1, 1, 1, 1, 1, 1, 1, 1, 1, 1, 1, 1, 1, 1, 1, 1, 1, 1, 1, 1,

1, 1, 1, 1, 1, 1, 1, 1, 1, 1, 1, 1, 1, 1, 1, 1, 1, 1, 1, 1,

1, 1, 1, 1, 1, 1, 1, 1, 1, 1, 1, 1, 1, 1, 1, 1, 1, 1, 1, 1,

1, 1, 1, 1, 1, 1, 1, 1, 1, 1, 1, 1, 1, 1, 1, 1, 1, 1, 1, 1,

1, 1, 1, 1, 1, 1, 1, 1, 1, 1, 1, 1, 1, 1, 1, 1, 1, 1, 1, 1,

1, 1, 1, 1, 1, 1, 1, 1, 1, 1, 1, 1, 1, 1, 1, 1, 1, 1, 1, 1,

1, 1, 1, 1, 1, 1, 1, 1, 1, 1, 1, 1, 1, 1, 1, 1, 1, 1, 1, 1,

1, 1, 1, 1, 1, 1, 1, 1, 1, 1, 1, 1, 1, 1, 1, 1, 1, 1, 1, 1,

1, 1, 1, 1, 1, 1, 1, 1, 1, 1, 1, 1, 1, 1, 1, 1, 1, 1, 1, 1,

1, 1, 1, 1, 1, 1, 1, 1, 1, 1, 1, 1, 1, 1, 1, 1, 1, 1, 1, 1,

1, 1, 1, 1, 1, 1, 1, 1, 1, 1, 1, 1, 1, 1, 1, 1, 1, 1, 1, 1,

1, 1, 1, 1, 1, 1, 1, 1, 1, 1, 1, 1, 1, 1, 1, 1, 1, 1, 1, 1,

1, 1, 1, 1, 1, 1, 1, 1, 1, 1, 1, 1, 1, 1, 1, 1, 1, 1, 1, 1,

1, 1, 1, 1, 1, 1, 1, 1, 1, 1, 1, 1, 1, 1, 1, 1, 1, 1, 1, 1,

1, 1, 1, 1, 1, 1, 1, 1, 1, 1, 1, 1, 1, 1, 1, 1, 1, 1, 1, 1,

1, 1, 1, 1, 1, 1, 1, 1, 1, 1, 1, 1, 1, 1, 1, 1, 1, 1, 1, 1,

1, 1, 1, 1, 1, 1, 1, 1, 1, 1, 1, 1, 1, 1, 2, 2, 2, 2, 2, 2,

2, 2, 2, 2, 2, 2, 2, 2, 2, 2, 2, 2, 2, 2, 2, 2, 2, 2, 2, 2,

2, 2, 2, 2, 2, 2, 2, 2, 2, 2, 2, 2, 2, 2, 2, 2, 2, 2, 2, 2,

2, 2, 2, 2, 2, 2, 2, 2, 2, 2, 2, 2, 2, 2, 2, 2, 2, 2, 2, 2,

2, 2, 2, 2, 2, 2, 2, 2, 2, 2, 2, 2, 2, 2, 2, 2, 2, 2, 2, 2,

2, 2, 2, 2, 2, 2, 2, 2, 2, 2, 2, 2, 2, 2, 2, 2, 2, 2, 2, 2,

2, 2, 2, 2, 2, 2, 2, 2, 2, 2, 2, 2, 2, 2, 2, 2, 2, 2, 2, 2,

2, 2, 2, 2, 2, 2, 2, 2, 2, 2, 2, 2, 2, 2, 2, 2, 2, 2, 2, 2,

2, 2, 2, 2, 2, 2, 2, 2, 2, 2, 2, 2, 2, 2, 2, 2, 2, 2, 2, 2,

2, 2, 2, 2, 2, 2, 2, 2, 2, 2, 2, 2, 2, 2, 2, 2, 2, 2, 2, 2,

2, 2, 2, 2, 2, 2, 2, 2, 2, 2, 2, 2, 2, 2, 2, 2, 2, 2, 2, 2,

2, 2, 2, 2, 2, 2, 2, 2, 2, 2, 2, 2, 2, 2, 2, 2, 2, 2, 2, 2,

2, 2, 2, 2, 2, 2, 2, 2, 2, 2, 2, 2, 2, 2, 2, 2, 2, 2, 2, 2,

2, 2, 2, 2, 2, 2, 2, 2, 2, 2, 2, 2, 2, 2, 2, 2, 2, 2, 2, 2,

2, 2, 2, 2, 2, 2, 2, 2, 2, 2, 2, 2, 2, 2, 2, 2, 2, 2, 2, 2,

2, 2, 2, 2, 2, 2, 2, 2, 2, 2, 2, 2, 2, 2, 2, 2, 2, 2, 2, 2,

2, 2, 2, 2, 2, 2, 2, 2, 2, 2, 2, 2, 2, 2, 2, 2, 2, 2, 2, 2,

2, 2, 2, 2, 2, 2, 2, 2, 2, 2, 2, 2, 2, 2, 2, 2, 2, 2, 2, 2,

2, 2, 2, 2, 2, 2, 2, 2, 2, 2, 2, 2, 2, 2, 2, 2, 2, 2, 2, 2,

2, 2, 2, 2, 2, 0, 0, 0, 0, 0, 0, 0, 0, 0, 0, 0, 0, 0, 0, 0,

# F BNA84-

1, 1, 1, 1, 1, 1, 1, 1, 1, 1, 1, 1, 1, 1, 1, 1, 1, 1, 1, 1,

1, 1, 1, 1, 1, 1, 1, 1, 1, 1, 1, 1, 1, 1, 1, 1, 1, 1, 1, 1,

1, 1, 1, 1, 1, 1, 1, 1, 1, 1, 1, 1, 1, 1, 1, 1, 1, 1, 1, 1,

1, 1, 1, 1, 1, 1, 1, 1, 1, 1, 1, 1, 1, 1, 1, 1, 1, 1, 1, 1,

1, 1, 1, 1, 1, 1, 1, 1, 1, 1, 1, 1, 1, 1, 1, 1, 1, 1, 1, 1,

1, 1, 1, 1, 1, 1, 1, 1, 1, 1, 1, 1, 1, 1, 1, 1, 1, 1, 1, 1,

1, 1, 1, 1, 1, 1, 1, 1, 1, 1, 1, 1, 1, 1, 1, 1, 1, 1, 1, 1,

1, 1, 1, 1, 1, 1, 1, 1, 1, 1, 1, 1, 1, 1, 1, 1, 1, 1, 1, 1,

1, 1, 1, 1, 1, 1, 1, 1, 1, 1, 1, 1, 1, 1, 1, 1, 1, 1, 1, 1,

1, 1, 1, 1, 1, 1, 1, 1, 1, 1, 1, 1, 1, 1, 1, 1, 1, 1, 1, 1,

1, 1, 1, 1, 1, 1, 1, 1, 1, 1, 1, 1, 1, 1, 1, 1, 1, 1, 1, 1,

1, 1, 1, 1, 1, 1, 1, 1, 1, 1, 1, 1, 1, 1, 1, 1, 1, 1, 1, 1,

1, 1, 1, 1, 1, 1, 1, 1, 1, 1, 1, 1, 1, 1, 1, 1, 1, 1, 1, 1,

1, 1, 1, 1, 1, 1, 1, 1, 1, 1, 1, 1, 1, 1, 1, 1, 1, 1, 1, 1,

1, 1, 1, 1, 1, 1, 1, 1, 1, 1, 1, 1, 1, 1, 1, 1, 1, 1, 1, 1,

1, 1, 1, 1, 1, 1, 1, 1, 1, 1, 1, 1, 1, 1, 1, 1, 1, 1, 1, 1,

1, 1, 1, 1, 1, 1, 1, 1, 1, 1, 1, 1, 1, 1, 1, 1, 1, 1, 1, 1,

1, 1, 1, 1, 1, 1, 1, 1, 1, 1, 1, 1, 1, 1, 1, 1, 1, 1, 1, 1,

1, 1, 1, 1, 1, 1, 1, 1, 1, 1, 1, 1, 1, 1, 1, 1, 1, 1, 1, 1,

1, 1, 1, 1, 1, 1, 1, 1, 1, 1, 1, 1, 1, 1, 1, 1, 1, 1, 1, 1,

1, 1, 1, 1, 1, 1, 1, 1, 1, 1, 1, 1, 1, 1, 1, 1, 1, 1, 1, 1,

1, 1, 1, 1, 1, 1, 1, 1, 1, 1, 1, 1, 1, 1, 1, 1, 1, 1, 1, 1,

1, 1, 1, 1, 1, 1, 1, 1, 1, 1, 1, 1, 1, 1, 1, 1, 1, 1, 1, 1,

1, 1, 1, 1, 1, 1, 1, 1, 1, 1, 1, 1, 1, 1, 1, 1, 1, 1, 1, 1,

1, 1, 1, 1, 1, 1, 1, 1, 1, 1, 1, 1, 1, 1, 1, 1, 1, 1, 1, 1,

1, 1, 1, 1, 1, 1, 1, 1, 1, 1, 1, 1, 1, 1, 1, 1, 1, 1, 1, 1,

1, 1, 1, 1, 1, 1, 1, 1, 1, 1, 1, 1, 1, 1, 1, 1, 1, 1, 1, 1,

1, 1, 1, 1, 1, 1, 1, 1, 1, 1, 1, 1, 1, 1, 1, 1, 1, 1, 1, 1,

1, 1, 1, 1, 1, 1, 1, 1, 1, 1, 1, 1, 1, 1, 1, 1, 1, 1, 1, 1,

1, 1, 1, 1, 1, 1, 1, 1, 1, 1, 1, 1, 1, 1, 1, 1, 1, 1, 1, 1,

1, 1, 1, 1, 1, 1, 1, 1, 1, 1, 1, 1, 1, 1, 1, 1, 1, 1, 1, 1,

1, 1, 1, 1, 1, 1, 1, 1, 1, 1, 1, 1, 1, 1, 1, 1, 1, 1, 1, 1,

1, 1, 2, 2, 2, 2, 2, 2, 2, 2, 2, 2, 2, 2, 2, 2, 2, 2, 2, 2,

2, 2, 2, 2, 2, 2, 2, 2, 2, 2, 2, 2, 2, 2, 2, 2, 2, 2, 2, 2,

2, 2, 2, 2, 2, 2, 2, 2, 2, 2, 2, 2, 2, 2, 2, 2, 2, 2, 2, 2,

2, 2, 2, 2, 2, 2, 2, 2, 2, 2, 2, 2, 2, 2, 2, 2, 2, 2, 2, 2,

2, 2, 2, 2, 2, 2, 2, 2, 2, 2, 2, 2, 2, 2, 2, 2, 2, 2, 2, 2,

2, 2, 2, 2, 2, 2, 2, 2, 2, 2, 2, 2, 2, 2, 2, 2, 2, 2, 2, 2,

2, 2, 2, 2, 2, 2, 2, 2, 2, 2, 2, 2, 2, 2, 2, 2, 2, 2, 2, 2,

2, 2, 2, 2, 2, 2, 2, 2, 2, 2, 2, 2, 2, 2, 2, 2, 2, 2, 2, 2,

2, 2, 2, 2, 2, 2, 2, 2, 2, 2, 2, 2, 2, 2, 2, 2, 2, 2, 2, 2,

2, 2, 2, 2, 2, 2, 2, 2, 2, 2, 2, 2, 2, 2, 2, 2, 2, 2, 2, 2,

2, 2, 2, 2, 2, 2, 2, 2, 2, 2, 2, 2, 2, 2, 2, 2, 2, 2, 2, 2,

2, 2, 2, 2, 2, 2, 2, 2, 2, 2, 2, 2, 2, 2, 2, 2, 2, 2, 2, 2,

2, 2, 2, 2, 2, 2, 2, 2, 2, 2, 2, 2, 2, 2, 2, 2, 2, 2, 2, 2,

2, 2, 2, 2, 2, 2, 2, 2, 2, 2, 2, 2, 2, 2, 2, 2, 2, 2, 2, 2,

2, 2, 2, 2, 2, 2, 2, 2, 2, 2, 2, 2, 2, 2, 2, 2, 2, 2, 2, 2,

2, 2, 2, 2, 2, 2, 2, 2, 2, 2, 2, 2, 2, 2, 2, 2, 2, 2, 2, 2,

2, 2, 2, 2, 2, 2, 2, 2, 2, 2, 2, 2, 2, 2, 2, 2, 2, 2, 2, 2,

2, 2, 2, 2, 2, 2, 2, 2, 2, 2, 2, 2, 2, 2, 2, 2, 2, 2, 2, 2,

2, 2, 2, 2, 2, 2, 2, 2, 2, 2, 2, 2, 2, 0, 0, 0, 0, 0, 0, 0,

0, 0, 0, 0, 0, 0, 0, 0, 0, 0, 0, 0, 0, 0, 0, 0, 0, 0, 0, 0,

0, 0, 0, 0, 0, 0, 0, 0, 0, 0, 0, 0, 0, 0, 0, 0, 0, 0, 0, 0,

0, 0, 0, 0, 0, 0, 0, 0, 0, 0, 0, 0, 0, 0, 0, 0, 0, 0, 0, 0,

0, 0, 0, 0, 0, 0, 0, 0, 0, 0, 0, 0, 0, 0, 0, 0, 0, 0, 0, 0,

0, 0, 0, 0, 0, 0, 0, 0, 0, 0, 0, 0, 0, 0, 0, 0, 0, 0, 0, 0,

0, 0, 0, 0, 0, 0, 0, 0, 0, 0, 0, 0, 0, 0, 0, 0, 0, 0, 0, 0,

0, 0, 0, 0, 0, 0, 0, 0, 0, 0, 0, 0, 0, 0, 0, 0, 0, 0, 0, 0,

0, 0, 0, 0, 0, 0, 0, 0, 0, 0, 0, 0, 0, 0, 0, 0, 0, 0, 0, 0,

0, 0, 0, 0, 0, 0, 0, 0, 0, 0, 0, 0, 0, 0, 0, 0, 0, 0, 0, 0,

0, 0, 0, 0, 0, 0, 0, 0, 0, 0, 0, 0, 0, 0, 0, 0, 0, 0, 0, 0,

0, 0, 0, 0, 0, 0, 0, 0, 0, 0, 0, 0, 0, 0, 0, 0, 0, 0, 0, 0,

0, 0, 0, 0, 0, 0, 0, 0, 0, 0, 0, 0, 0, 0, 0, 0, 0, 0, 0, 0,

0, 0, 0, 0, 0, 0, 0, 0, 0, 0, 0, 0, 0, 0, 0, 0, 0, 0, 0, 0,

0, 0, 0, 0, 0, 0, 0, 0, 0, 0, 0, 0, 0, 0, 0, 0, 0, 0, 0, 0,

0, 0, 0, 0, 0, 0, 0, 0, 0, 0, 0, 0, 0, 0, 0, 0, 0, 0, 0, 0,

0, 0, 0, 0, 0, 0, 0, 0, 0, 0, 0, 0, 0, 0, 0, 0, 0, 0, 0, 0,

0, 0, 0, 0, 0, 0, 0, 0, 0, 0, 0, 0, 0, 0, 0, 0, 0, 0, 0, 0,

0, 0, 0, 0, 0, 0, 0, 0, 0, 0, 0, 0, 0, 0, 0, 0, 0, 0, 0, 0,

0, 0, 0, 0, 0, 0, 0, 0, 0, 0, 0, 0, 0, 0, 0, 0, 0, 0, 0, 0,

# F HIC85-89

1, 1, 1, 1, 1, 1, 1, 1, 1, 1, 1, 1, 1, 1, 1, 1, 1, 1, 1, 1,

1, 1, 1, 1, 1, 1, 1, 1, 1, 1, 1, 1, 1, 1, 1, 1, 1, 1, 1, 1,

1, 1, 1, 1, 1, 1, 1, 1, 1, 1, 1, 1, 1, 1, 1, 1, 1, 1, 1, 1,

1, 1, 1, 1, 1, 1, 1, 1, 1, 1, 1, 1, 1, 1, 1, 1, 1, 1, 1, 1,

1, 1, 1, 1, 1, 1, 1, 1, 1, 1, 1, 1, 1, 1, 1, 1, 1, 1, 1, 1,

1, 1, 1, 1, 1, 1, 1, 1, 1, 1, 1, 1, 1, 1, 1, 1, 1, 1, 1, 1,

1, 1, 1, 1, 1, 1, 1, 1, 1, 1, 1, 1, 1, 1, 1, 1, 1, 1, 1, 1,

1, 1, 1, 1, 1, 1, 1, 1, 1, 1, 1, 1, 1, 1, 1, 1, 1, 1, 1, 1,

1, 1, 1, 1, 1, 1, 1, 1, 1, 1, 1, 1, 1, 1, 1, 1, 1, 1, 1, 1,

1, 1, 1, 1, 1, 1, 1, 1, 1, 1, 1, 1, 1, 1, 1, 1, 1, 1, 1, 1,

1, 1, 1, 1, 1, 1, 1, 1, 1, 1, 1, 1, 1, 1, 1, 1, 1, 1, 1, 1,

1, 1, 1, 1, 1, 1, 1, 1, 1, 1, 1, 1, 1, 1, 1, 1, 1, 1, 1, 1,

1, 1, 1, 1, 1, 1, 1, 1, 1, 1, 1, 1, 1, 1, 1, 1, 1, 1, 1, 1,

1, 1, 1, 1, 1, 1, 1, 1, 1, 1, 1, 1, 1, 1, 1, 1, 1, 1, 1, 1,

1, 1, 1, 1, 1, 1, 1, 1, 1, 1, 1, 1, 1, 1, 1, 1, 1, 1, 1, 1,

1, 1, 1, 1, 1, 1, 1, 1, 1, 1, 1, 1, 1, 1, 1, 1, 1, 1, 1, 1,

1, 1, 1, 1, 1, 1, 1, 1, 1, 1, 1, 1, 1, 1, 1, 1, 1, 1, 1, 1,

1, 1, 1, 1, 1, 1, 1, 1, 1, 1, 1, 1, 1, 1, 1, 1, 1, 1, 1, 1,

1, 1, 1, 1, 1, 1, 1, 1, 1, 1, 1, 1, 1, 1, 1, 1, 1, 1, 1, 1,

1, 1, 1, 1, 1, 1, 1, 1, 1, 1, 1, 1, 1, 1, 1, 1, 1, 1, 1, 1,

1, 1, 1, 1, 1, 1, 1, 1, 1, 1, 1, 1, 1, 1, 1, 1, 1, 1, 1, 1,

1, 1, 1, 1, 1, 1, 1, 1, 1, 1, 1, 1, 1, 1, 1, 1, 1, 1, 1, 1,

1, 1, 1, 1, 1, 1, 1, 1, 1, 1, 1, 1, 1, 1, 1, 1, 1, 1, 1, 1,

1, 1, 1, 1, 1, 1, 1, 1, 1, 1, 1, 1, 1, 1, 1, 1, 1, 1, 1, 1,

1, 1, 1, 1, 1, 1, 1, 1, 1, 1, 1, 1, 1, 1, 1, 1, 1, 1, 1, 1,

1, 1, 1, 1, 1, 1, 1, 1, 1, 1, 1, 1, 1, 1, 1, 1, 1, 1, 1, 1,

1, 1, 1, 1, 1, 1, 1, 1, 1, 1, 1, 1, 1, 1, 1, 1, 1, 1, 1, 1,

1, 1, 1, 1, 1, 1, 1, 1, 1, 1, 1, 1, 1, 1, 1, 1, 1, 1, 1, 1,

1, 1, 1, 1, 1, 1, 1, 1, 1, 1, 1, 1, 1, 1, 1, 1, 1, 1, 1, 1,

1, 1, 1, 1, 1, 1, 1, 1, 1, 1, 1, 1, 1, 1, 1, 1, 1, 1, 1, 1,

1, 1, 1, 1, 1, 1, 1, 1, 1, 1, 1, 1, 1, 1, 1, 1, 1, 1, 1, 1,

1, 1, 1, 1, 1, 1, 1, 1, 1, 1, 1, 1, 1, 1, 1, 1, 1, 1, 1, 1,

1, 1, 1, 1, 1, 1, 1, 1, 1, 1, 1, 1, 1, 1, 1, 1, 1, 1, 1, 1,

1, 1, 1, 1, 1, 1, 1, 1, 1, 1, 1, 1, 1, 1, 1, 1, 1, 1, 1, 1,

1, 1, 1, 1, 1, 1, 1, 1, 1, 1, 1, 1, 1, 1, 1, 1, 1, 1, 1, 1,

1, 1, 1, 1, 2, 2, 2, 2, 2, 2, 2, 2, 2, 2, 2, 2, 2, 2, 2, 2,

2, 2, 2, 2, 2, 2, 2, 2, 2, 2, 2, 2, 2, 2, 2, 2, 2, 2, 2, 2,

2, 2, 2, 2, 2, 2, 2, 2, 2, 2, 2, 2, 2, 2, 2, 2, 2, 2, 2, 2,

2, 2, 2, 2, 2, 2, 2, 2, 2, 2, 2, 2, 2, 2, 2, 2, 2, 2, 2, 2,

2, 2, 2, 2, 2, 2, 2, 2, 2, 2, 2, 2, 2, 2, 2, 2, 2, 2, 2, 2,

2, 2, 2, 2, 2, 2, 2, 2, 2, 2, 2, 2, 2, 2, 2, 2, 2, 2, 2, 2,

2, 2, 2, 2, 2, 2, 2, 2, 2, 2, 2, 2, 2, 2, 2, 2, 2, 2, 2, 2,

2, 2, 2, 2, 2, 2, 2, 2, 2, 2, 2, 2, 2, 2, 2, 2, 2, 2, 0, 0,

0, 0, 0, 0, 0, 0, 0, 0, 0, 0, 0, 0, 0, 0, 0, 0, 0, 0, 0, 0,

0, 0, 0, 0, 0, 0, 0, 0, 0, 0, 0, 0, 0, 0, 0, 0, 0, 0, 0, 0,

0, 0, 0, 0, 0, 0, 0, 0, 0, 0, 0, 0, 0, 0, 0, 0, 0, 0, 0, 0,

0, 0, 0, 0, 0, 0, 0, 0, 0, 0, 0, 0, 0, 0, 0, 0, 0, 0, 0, 0,

0, 0, 0, 0, 0, 0, 0, 0, 0, 0, 0, 0, 0, 0, 0, 0, 0, 0, 0, 0,

0, 0, 0, 0, 0, 0, 0, 0, 0, 0, 0, 0, 0, 0, 0, 0, 0, 0, 0, 0,

0, 0, 0, 0, 0, 0, 0, 0, 0, 0, 0, 0, 0, 0, 0, 0, 0, 0, 0, 0,

0, 0, 0, 0, 0, 0, 0, 0, 0, 0, 0, 0, 0, 0, 0, 0, 0, 0, 0, 0,

0, 0, 0, 0, 0, 0, 0, 0, 0, 0, 0, 0, 0, 0, 0, 0, 0, 0, 0, 0,

0, 0, 0, 0, 0, 0, 0, 0, 0, 0, 0, 0, 0, 0, 0, 0, 0, 0, 0, 0,

0, 0, 0, 0, 0, 0, 0, 0, 0, 0, 0, 0, 0, 0, 0, 0, 0, 0, 0, 0,

0, 0, 0, 0, 0, 0, 0, 0, 0, 0, 0, 0, 0, 0, 0, 0, 0, 0, 0, 0,

0, 0, 0, 0, 0, 0, 0, 0, 0, 0, 0, 0, 0, 0, 0, 0, 0, 0, 0, 0,

0, 0, 0, 0, 0, 0, 0, 0, 0, 0, 0, 0, 0, 0, 0, 0, 0, 0, 0, 0,

0, 0, 0, 0, 0, 0, 0, 0, 0, 0, 0, 0, 0, 0, 0, 0, 0, 0, 0, 0,

0, 0, 0, 0, 0, 0, 0, 0, 0, 0, 0, 0, 0, 0, 0, 0, 0, 0, 0, 0,

0, 0, 0, 0, 0, 0, 0, 0, 0, 0, 0, 0, 0, 0, 0, 0, 0, 0, 0, 0,

0, 0, 0, 0, 0, 0, 0, 0, 0, 0, 0, 0, 0, 0, 0, 0, 0, 0, 0, 0,

0, 0, 0, 0, 0, 0, 0, 0, 0, 0, 0, 0, 0, 0, 0, 0, 0, 0, 0, 0,

0, 0, 0, 0, 0, 0, 0, 0, 0, 0, 0, 0, 0, 0, 0, 0, 0, 0, 0, 0,

0, 0, 0, 0, 0, 0, 0, 0, 0, 0, 0, 0, 0, 0, 0, 0, 0, 0, 0, 0,

0, 0, 0, 0, 0, 0, 0, 0, 0, 0, 0, 0, 0, 0, 0, 0, 0, 0, 0, 0,

0, 0, 0, 0, 0, 0, 0, 0, 0, 0, 0, 0, 0, 0, 0, 0, 0, 0, 0, 0,

0, 0, 0, 0, 0, 0, 0, 0, 0, 0, 0, 0, 0, 0, 0, 0, 0, 0, 0, 0,

0, 0, 0, 0, 0, 0, 0, 0, 0, 0, 0, 0, 0, 0, 0, 0, 0, 0, 0, 0,

0, 0, 0, 0, 0, 0, 0, 0, 0, 0, 0, 0, 0, 0, 0, 0, 0, 0, 0, 0,

0, 0, 0, 0, 0, 0, 0, 0, 0, 0, 0, 0, 0, 0, 0, 0, 0, 0, 0, 0,

#F BNA85-90

1, 1, 1, 1, 1, 1, 1, 1, 1, 1, 1, 1, 1, 1, 1, 1, 1, 1, 1, 1,

1, 1, 1, 1, 1, 1, 1, 1, 1, 1, 1, 1, 1, 1, 1, 1, 1, 1, 1, 1,

1, 1, 1, 1, 1, 1, 1, 1, 1, 1, 1, 1, 1, 1, 1, 1, 1, 1, 1, 1,

1, 1, 1, 1, 1, 1, 1, 1, 1, 1, 1, 1, 1, 1, 1, 1, 1, 1, 1, 1,

1, 1, 1, 1, 1, 1, 1, 1, 1, 1, 1, 1, 1, 1, 1, 1, 1, 1, 1, 1,

1, 1, 1, 1, 1, 1, 1, 1, 1, 1, 1, 1, 1, 1, 1, 1, 1, 1, 1, 1,

1, 1, 1, 1, 1, 1, 1, 1, 1, 1, 1, 1, 1, 1, 1, 1, 1, 1, 1, 1,

1, 1, 1, 1, 1, 1, 1, 1, 1, 1, 1, 1, 1, 1, 1, 1, 1, 1, 1, 1,

1, 1, 1, 1, 1, 1, 1, 1, 1, 1, 1, 1, 1, 1, 1, 1, 1, 1, 1, 1,

1, 1, 1, 1, 1, 1, 1, 1, 1, 1, 1, 1, 1, 1, 1, 1, 1, 1, 1, 1,

1, 1, 1, 1, 1, 1, 1, 1, 1, 1, 1, 1, 1, 1, 1, 1, 1, 1, 1, 1,

1, 1, 1, 1, 1, 1, 1, 1, 1, 1, 1, 1, 1, 1, 1, 1, 1, 1, 1, 1,

1, 1, 1, 1, 1, 1, 1, 1, 1, 1, 1, 1, 1, 1, 1, 1, 1, 1, 1, 1,

1, 1, 1, 1, 1, 1, 1, 1, 1, 1, 1, 1, 1, 1, 1, 1, 1, 1, 1, 1,

1, 1, 1, 1, 1, 1, 1, 1, 1, 1, 1, 1, 1, 1, 1, 1, 1, 1, 1, 1,

1, 1, 1, 1, 1, 1, 1, 1, 1, 1, 1, 1, 1, 1, 1, 1, 1, 1, 1, 1,

1, 1, 1, 1, 1, 1, 1, 1, 1, 1, 1, 1, 1, 1, 1, 1, 1, 1, 1, 1,

1, 1, 1, 1, 1, 1, 1, 1, 1, 1, 1, 1, 1, 1, 1, 1, 1, 1, 1, 1,

1, 1, 1, 1, 1, 1, 1, 1, 1, 1, 1, 1, 1, 1, 1, 1, 1, 1, 1, 1,

1, 1, 1, 1, 1, 1, 1, 1, 1, 1, 1, 1, 1, 1, 1, 1, 1, 1, 1, 1,

1, 1, 1, 1, 1, 2, 2, 2, 2, 2, 2, 2, 2, 2, 2, 2, 2, 2, 2, 2,

2, 2, 2, 2, 2, 2, 2, 2, 2, 2, 2, 2, 2, 2, 2, 2, 2, 2, 2, 2,

2, 2, 2, 2, 2, 2, 2, 2, 2, 2, 2, 2, 2, 2, 2, 2, 2, 2, 2, 2,

2, 2, 2, 2, 2, 2, 2, 2, 2, 2, 2, 2, 2, 2, 2, 2, 2, 2, 2, 2,

2, 2, 2, 2, 2, 2, 2, 2, 2, 2, 2, 2, 2, 2, 2, 2, 2, 2, 2, 2,

2, 2, 2, 2, 2, 2, 2, 2, 2, 2, 2, 2, 2, 2, 2, 2, 2, 2, 2, 2,

2, 2, 2, 2, 2, 2, 2, 2, 2, 2, 2, 2, 2, 2, 2, 2, 2, 2, 2, 2,

2, 2, 2, 2, 2, 2, 2, 2, 2, 2, 2, 2, 2, 2, 2, 2, 2, 2, 2, 0,

0, 0, 0, 0, 0, 0, 0, 0, 0, 0, 0, 0, 0, 0, 0, 0, 0, 0, 0, 0,

0, 0, 0, 0, 0, 0, 0, 0, 0, 0, 0, 0, 0, 0, 0, 0, 0, 0, 0, 0,

0, 0, 0, 0, 0, 0, 0, 0, 0, 0, 0, 0, 0, 0, 0, 0, 0, 0, 0, 0,

0, 0, 0, 0, 0, 0, 0, 0, 0, 0, 0, 0, 0, 0, 0, 0, 0, 0, 0, 0,

0, 0, 0, 0, 0, 0, 0, 0, 0, 0, 0, 0, 0, 0, 0, 0, 0, 0, 0, 0,

0, 0, 0, 0, 0, 0, 0, 0, 0, 0, 0, 0, 0, 0, 0, 0, 0, 0, 0, 0,

0, 0, 0, 0, 0, 0, 0, 0, 0, 0, 0, 0, 0, 0, 0, 0, 0, 0, 0, 0,

0, 0, 0, 0, 0, 0, 0, 0, 0, 0, 0, 0, 0, 0, 0, 0, 0, 0, 0, 0,

0, 0, 0, 0, 0, 0, 0, 0, 0, 0, 0, 0, 0, 0, 0, 0, 0, 0, 0, 0,

0, 0, 0, 0, 0, 0, 0, 0, 0, 0, 0, 0, 0, 0, 0, 0, 0, 0, 0, 0,

0, 0, 0, 0, 0, 0, 0, 0, 0, 0, 0, 0, 0, 0, 0, 0, 0, 0, 0, 0,

0, 0, 0, 0, 0, 0, 0, 0, 0, 0, 0, 0, 0, 0, 0, 0, 0, 0, 0, 0,

0, 0, 0, 0, 0, 0, 0, 0, 0, 0, 0, 0, 0, 0, 0, 0, 0, 0, 0, 0,

0, 0, 0, 0, 0, 0, 0, 0, 0, 0, 0, 0, 0, 0, 0, 0, 0, 0, 0, 0,

0, 0, 0, 0, 0, 0, 0, 0, 0, 0, 0, 0, 0, 0, 0, 0, 0, 0, 0, 0,

0, 0, 0, 0, 0, 0, 0, 0, 0, 0, 0, 0, 0, 0, 0, 0, 0, 0, 0, 0,

0, 0, 0, 0, 0, 0, 0, 0, 0, 0, 0, 0, 0, 0, 0, 0, 0, 0, 0, 0,

0, 0, 0, 0, 0, 0, 0, 0, 0, 0, 0, 0, 0, 0, 0, 0, 0, 0, 0, 0,

0, 0, 0, 0, 0, 0, 0, 0, 0, 0, 0, 0, 0, 0, 0, 0, 0, 0, 0, 0,

0, 0, 0, 0, 0, 0, 0, 0, 0, 0, 0, 0, 0, 0, 0, 0, 0, 0, 0, 0,

0, 0, 0, 0, 0, 0, 0, 0, 0, 0, 0, 0, 0, 0, 0, 0, 0, 0, 0, 0,

0, 0, 0, 0, 0, 0, 0, 0, 0, 0, 0, 0, 0, 0, 0, 0, 0, 0, 0, 0,

0, 0, 0, 0, 0, 0, 0, 0, 0, 0, 0, 0, 0, 0, 0, 0, 0, 0, 0, 0,

0, 0, 0, 0, 0, 0, 0, 0, 0, 0, 0, 0, 0, 0, 0, 0, 0, 0, 0, 0,

0, 0, 0, 0, 0, 0, 0, 0, 0, 0, 0, 0, 0, 0, 0, 0, 0, 0, 0, 0,

0, 0, 0, 0, 0, 0, 0, 0, 0, 0, 0, 0, 0, 0, 0, 0, 0, 0, 0, 0,

0, 0, 0, 0, 0, 0, 0, 0, 0, 0, 0, 0, 0, 0, 0, 0, 0, 0, 0, 0,

0, 0, 0, 0, 0, 0, 0, 0, 0, 0, 0, 0, 0, 0, 0, 0, 0, 0, 0, 0,

0, 0, 0, 0, 0, 0, 0, 0, 0, 0, 0, 0, 0, 0, 0, 0, 0, 0, 0, 0,

0, 0, 0, 0, 0, 0, 0, 0, 0, 0, 0, 0, 0, 0, 0, 0, 0, 0, 0, 0,

0, 0, 0, 0, 0, 0, 0, 0, 0, 0, 0, 0, 0, 0, 0, 0, 0, 0, 0, 0,

0, 0, 0, 0, 0, 0, 0, 0, 0, 0, 0, 0, 0, 0, 0, 0, 0, 0, 0, 0,

0, 0, 0, 0, 0, 0, 0, 0, 0, 0, 0, 0, 0, 0, 0, 0, 0, 0, 0, 0,

0, 0, 0, 0, 0, 0, 0, 0, 0, 0, 0, 0, 0, 0, 0, 0, 0, 0, 0, 0,

0, 0, 0, 0, 0, 0, 0, 0, 0, 0, 0, 0, 0, 0, 0, 0, 0, 0, 0, 0,

0, 0, 0, 0, 0, 0, 0, 0, 0, 0, 0, 0, 0, 0, 0, 0, 0, 0, 0, 0,

0, 0, 0, 0, 0, 0, 0, 0, 0, 0, 0, 0, 0, 0, 0, 0, 0, 0, 0, 0,

0, 0, 0, 0, 0, 0, 0, 0, 0, 0, 0, 0, 0, 0, 0, 0, 0, 0, 0, 0,

0, 0, 0, 0, 0, 0, 0, 0, 0, 0, 0, 0, 0, 0, 0, 0, 0, 0, 0, 0,

0, 0, 0, 0, 0, 0, 0, 0, 0, 0, 0, 0, 0, 0, 0, 0, 0, 0, 0, 0,

0, 0, 0, 0, 0, 0, 0, 0, 0, 0, 0, 0, 0, 0, 0, 0, 0, 0, 0, 0,

0, 0, 0, 0, 0, 0, 0, 0, 0, 0, 0, 0, 0, 0, 0, 0, 0, 0, 0, 0,

#F HIC 90+

1, 1, 1, 1, 1, 1, 1, 1, 1, 1, 1, 1, 1, 1, 1, 1, 1, 1, 1, 1,

1, 1, 1, 1, 1, 1, 1, 1, 1, 1, 1, 1, 1, 1, 1, 1, 1, 1, 1, 1,

1, 1, 1, 1, 1, 1, 1, 1, 1, 1, 1, 1, 1, 1, 1, 1, 1, 1, 1, 1,

1, 1, 1, 1, 1, 1, 1, 1, 1, 1, 1, 1, 1, 1, 1, 1, 1, 1, 1, 1,

1, 1, 1, 1, 1, 1, 1, 1, 1, 1, 1, 1, 1, 1, 1, 1, 1, 1, 1, 1,

1, 1, 1, 1, 1, 1, 1, 1, 1, 1, 1, 1, 1, 1, 1, 1, 1, 1, 1, 1,

1, 1, 1, 1, 1, 1, 1, 1, 1, 1, 1, 1, 1, 1, 1, 1, 1, 1, 1, 1,

1, 1, 1, 1, 1, 1, 1, 1, 1, 1, 1, 1, 1, 1, 1, 1, 1, 1, 1, 1,

1, 1, 1, 1, 1, 1, 1, 1, 1, 1, 1, 1, 1, 1, 1, 1, 1, 1, 1, 1,

1, 1, 1, 1, 1, 1, 1, 1, 1, 1, 1, 1, 1, 1, 1, 1, 1, 1, 1, 1,

1, 1, 1, 1, 1, 1, 1, 1, 1, 1, 1, 1, 1, 1, 1, 1, 1, 1, 1, 1,

1, 1, 1, 1, 1, 1, 1, 1, 1, 1, 1, 1, 1, 1, 1, 1, 1, 1, 1, 1,

1, 1, 1, 1, 1, 1, 1, 1, 1, 1, 1, 1, 1, 1, 1, 1, 1, 1, 1, 1,

1, 1, 1, 1, 1, 1, 1, 1, 1, 1, 1, 1, 1, 1, 1, 1, 1, 1, 1, 1,

1, 1, 1, 1, 1, 1, 1, 1, 1, 1, 1, 1, 1, 1, 1, 1, 1, 1, 1, 1,

1, 1, 1, 1, 1, 1, 1, 1, 1, 1, 1, 1, 1, 1, 1, 1, 1, 1, 1, 1,

1, 1, 1, 1, 1, 1, 1, 1, 1, 1, 1, 1, 1, 1, 1, 1, 1, 1, 1, 1,

1, 1, 1, 1, 1, 1, 1, 1, 1, 1, 1, 1, 1, 1, 1, 1, 1, 1, 1, 1,

1, 1, 1, 1, 1, 1, 1, 1, 1, 1, 1, 1, 1, 1, 1, 1, 1, 1, 1, 1,

1, 1, 1, 1, 1, 1, 1, 1, 1, 1, 1, 1, 1, 1, 1, 1, 1, 1, 1, 1,

1, 1, 1, 1, 1, 1, 1, 1, 1, 1, 1, 1, 1, 1, 1, 1, 1, 1, 1, 1,

1, 1, 1, 1, 1, 1, 1, 1, 1, 1, 1, 1, 1, 1, 1, 1, 1, 1, 1, 1,

1, 1, 1, 1, 1, 1, 1, 1, 1, 1, 1, 1, 1, 1, 1, 1, 1, 1, 1, 1,

1, 1, 1, 1, 1, 1, 1, 1, 1, 1, 1, 1, 1, 1, 1, 1, 1, 1, 1, 1,

1, 1, 1, 1, 1, 1, 1, 1, 1, 1, 1, 1, 1, 1, 1, 1, 1, 1, 1, 1,

1, 1, 1, 1, 1, 1, 1, 1, 1, 1, 1, 1, 1, 1, 1, 1, 1, 1, 1, 1,

1, 1, 1, 1, 1, 1, 1, 1, 1, 1, 1, 1, 1, 1, 1, 1, 1, 1, 1, 1,

1, 1, 1, 1, 1, 1, 1, 1, 1, 1, 1, 1, 1, 1, 1, 1, 2, 2, 2, 2,

2, 2, 2, 2, 2, 2, 2, 2, 2, 2, 2, 2, 2, 2, 2, 2, 2, 2, 2, 2,

2, 2, 2, 2, 2, 2, 2, 2, 2, 2, 2, 2, 2, 2, 2, 2, 2, 2, 2, 2,

2, 2, 2, 2, 2, 2, 2, 2, 2, 2, 2, 2, 2, 2, 2, 2, 2, 2, 2, 2,

2, 2, 2, 2, 2, 2, 2, 2, 2, 2, 2, 2, 2, 2, 2, 2, 2, 2, 2, 2,

2, 2, 0, 0, 0, 0, 0, 0, 0, 0, 0, 0, 0, 0, 0, 0, 0, 0, 0, 0,

0, 0, 0, 0, 0, 0, 0, 0, 0, 0, 0, 0, 0, 0, 0, 0, 0, 0, 0, 0,

0, 0, 0, 0, 0, 0, 0, 0, 0, 0, 0, 0, 0, 0, 0, 0, 0, 0, 0, 0,

0, 0, 0, 0, 0, 0, 0, 0, 0, 0, 0, 0, 0, 0, 0, 0, 0, 0, 0, 0,

0, 0, 0, 0, 0, 0, 0, 0, 0, 0, 0, 0, 0, 0, 0, 0, 0, 0, 0, 0,

0, 0, 0, 0, 0, 0, 0, 0, 0, 0, 0, 0, 0, 0, 0, 0, 0, 0, 0, 0,

0, 0, 0, 0, 0, 0, 0, 0, 0, 0, 0, 0, 0, 0, 0, 0, 0, 0, 0, 0,

0, 0, 0, 0, 0, 0, 0, 0, 0, 0, 0, 0, 0, 0, 0, 0, 0, 0, 0, 0,

0, 0, 0, 0, 0, 0, 0, 0, 0, 0, 0, 0, 0, 0, 0, 0, 0, 0, 0, 0,

0, 0, 0, 0, 0, 0, 0, 0, 0, 0, 0, 0, 0, 0, 0, 0, 0, 0, 0, 0,

0, 0, 0, 0, 0, 0, 0, 0, 0, 0, 0, 0, 0, 0, 0, 0, 0, 0, 0, 0,

0, 0, 0, 0, 0, 0, 0, 0, 0, 0, 0, 0, 0, 0, 0, 0, 0, 0, 0, 0,

0, 0, 0, 0, 0, 0, 0, 0, 0, 0, 0, 0, 0, 0, 0, 0, 0, 0, 0, 0,

0, 0, 0, 0, 0, 0, 0, 0, 0, 0, 0, 0, 0, 0, 0, 0, 0, 0, 0, 0,

0, 0, 0, 0, 0, 0, 0, 0, 0, 0, 0, 0, 0, 0, 0, 0, 0, 0, 0, 0,

0, 0, 0, 0, 0, 0, 0, 0, 0, 0, 0, 0, 0, 0, 0, 0, 0, 0, 0, 0,

0, 0, 0, 0, 0, 0, 0, 0, 0, 0, 0, 0, 0, 0, 0, 0, 0, 0, 0, 0,

0, 0, 0, 0, 0, 0, 0, 0, 0, 0, 0, 0, 0, 0, 0, 0, 0, 0, 0, 0,

0, 0, 0, 0, 0, 0, 0, 0, 0, 0, 0, 0, 0, 0, 0, 0, 0, 0, 0, 0,

0, 0, 0, 0, 0, 0, 0, 0, 0, 0, 0, 0, 0, 0, 0, 0, 0, 0, 0, 0,

0, 0, 0, 0, 0, 0, 0, 0, 0, 0, 0, 0, 0, 0, 0, 0, 0, 0, 0, 0,

0, 0, 0, 0, 0, 0, 0, 0, 0, 0, 0, 0, 0, 0, 0, 0, 0, 0, 0, 0,

0, 0, 0, 0, 0, 0, 0, 0, 0, 0, 0, 0, 0, 0, 0, 0, 0, 0, 0, 0,

0, 0, 0, 0, 0, 0, 0, 0, 0, 0, 0, 0, 0, 0, 0, 0, 0, 0, 0, 0,

0, 0, 0, 0, 0, 0, 0, 0, 0, 0, 0, 0, 0, 0, 0, 0, 0, 0, 0, 0,

0, 0, 0, 0, 0, 0, 0, 0, 0, 0, 0, 0, 0, 0, 0, 0, 0, 0, 0, 0,

0, 0, 0, 0, 0, 0, 0, 0, 0, 0, 0, 0, 0, 0, 0, 0, 0, 0, 0, 0,

0, 0, 0, 0, 0, 0, 0, 0, 0, 0, 0, 0, 0, 0, 0, 0, 0, 0, 0, 0,

0, 0, 0, 0, 0, 0, 0, 0, 0, 0, 0, 0, 0, 0, 0, 0, 0, 0, 0, 0,

0, 0, 0, 0, 0, 0, 0, 0, 0, 0, 0, 0, 0, 0, 0, 0, 0, 0, 0, 0,

0, 0, 0, 0, 0, 0, 0, 0, 0, 0, 0, 0, 0, 0, 0, 0, 0, 0, 0, 0,

0, 0, 0, 0, 0, 0, 0, 0, 0, 0, 0, 0, 0, 0, 0, 0, 0, 0, 0, 0,

0, 0, 0, 0, 0, 0, 0, 0, 0, 0, 0, 0, 0, 0, 0, 0, 0, 0, 0, 0,

0, 0, 0, 0, 0, 0, 0, 0, 0, 0, 0, 0, 0, 0, 0, 0, 0, 0, 0, 0,

0, 0, 0, 0, 0, 0, 0, 0, 0, 0, 0, 0, 0, 0, 0, 0, 0, 0, 0, 0,

0, 0, 0, 0, 0, 0, 0, 0, 0, 0, 0, 0, 0, 0, 0, 0, 0, 0, 0, 0,

0, 0, 0, 0, 0, 0, 0, 0, 0, 0, 0, 0, 0, 0, 0, 0, 0, 0, 0, 0,

0, 0, 0, 0, 0, 0, 0, 0, 0, 0, 0, 0, 0, 0, 0, 0, 0, 0, 0, 0,

#F BNA90+

1, 1, 1, 1, 1, 1, 1, 1, 1, 1, 1, 1, 1, 1, 1, 1, 1, 1, 1, 1,

1, 1, 1, 1, 1, 1, 1, 1, 1, 1, 1, 1, 1, 1, 1, 1, 1, 1, 1, 1,

1, 1, 1, 1, 1, 1, 1, 1, 1, 1, 1, 1, 1, 1, 1, 1, 1, 1, 1, 1,

1, 1, 1, 1, 1, 1, 1, 1, 1, 1, 1, 1, 1, 1, 1, 1, 1, 1, 1, 1,

1, 1, 1, 1, 1, 1, 1, 1, 1, 1, 1, 1, 1, 1, 1, 1, 1, 1, 1, 1,

1, 1, 1, 1, 1, 1, 1, 1, 1, 1, 1, 1, 1, 1, 1, 1, 1, 1, 1, 1,

1, 1, 1, 1, 1, 1, 1, 1, 1, 1, 1, 1, 1, 1, 1, 1, 1, 1, 1, 1,

1, 1, 1, 1, 1, 1, 1, 1, 1, 1, 1, 1, 2, 2, 2, 2, 2, 2, 2, 2,

2, 2, 2, 2, 2, 2, 2, 2, 2, 2, 2, 2, 2, 2, 2, 2, 2, 2, 2, 2,

2, 2, 2, 2, 2, 2, 2, 2, 2, 2, 2, 2, 2, 2, 2, 2, 2, 2, 2, 2,

2, 2, 2, 2, 2, 2, 2, 2, 2, 2, 2, 2, 2, 2, 2, 2, 2, 2, 2, 2,

2, 2, 2, 2, 2, 2, 2, 2, 2, 2, 2, 2, 2, 2, 2, 2, 2, 2, 0, 0,

0, 0, 0, 0, 0, 0, 0, 0, 0, 0, 0, 0, 0, 0, 0, 0, 0, 0, 0, 0,

0, 0, 0, 0, 0, 0, 0, 0, 0, 0, 0, 0, 0, 0, 0, 0, 0, 0, 0, 0,

0, 0, 0, 0, 0, 0, 0, 0, 0, 0, 0, 0, 0, 0, 0, 0, 0, 0, 0, 0,

0, 0, 0, 0, 0, 0, 0, 0, 0, 0, 0, 0, 0, 0, 0, 0, 0, 0, 0, 0,

0, 0, 0, 0, 0, 0, 0, 0, 0, 0, 0, 0, 0, 0, 0, 0, 0, 0, 0, 0,

0, 0, 0, 0, 0, 0, 0, 0, 0, 0, 0, 0, 0, 0, 0, 0, 0, 0, 0, 0,

0, 0, 0, 0, 0, 0, 0, 0, 0, 0, 0, 0, 0, 0, 0, 0, 0, 0, 0, 0,

0, 0, 0, 0, 0, 0, 0, 0, 0, 0, 0, 0, 0, 0, 0, 0, 0, 0, 0, 0,

0, 0, 0, 0, 0, 0, 0, 0, 0, 0, 0, 0, 0, 0, 0, 0, 0, 0, 0, 0,

0, 0, 0, 0, 0, 0, 0, 0, 0, 0, 0, 0, 0, 0, 0, 0, 0, 0, 0, 0,

0, 0, 0, 0, 0, 0, 0, 0, 0, 0, 0, 0, 0, 0, 0, 0, 0, 0, 0, 0,

0, 0, 0, 0, 0, 0, 0, 0, 0, 0, 0, 0, 0, 0, 0, 0, 0, 0, 0, 0,

0, 0, 0, 0, 0, 0, 0, 0, 0, 0, 0, 0, 0, 0, 0, 0, 0, 0, 0, 0,

0, 0, 0, 0, 0, 0, 0, 0, 0, 0, 0, 0, 0, 0, 0, 0, 0, 0, 0, 0,

0, 0, 0, 0, 0, 0, 0, 0, 0, 0, 0, 0, 0, 0, 0, 0, 0, 0, 0, 0,

0, 0, 0, 0, 0, 0, 0, 0, 0, 0, 0, 0, 0, 0, 0, 0, 0, 0, 0, 0,

0, 0, 0, 0, 0, 0, 0, 0, 0, 0, 0, 0, 0, 0, 0, 0, 0, 0, 0, 0,

0, 0, 0, 0, 0, 0, 0, 0, 0, 0, 0, 0, 0, 0, 0, 0, 0, 0, 0, 0,

0, 0, 0, 0, 0, 0, 0, 0, 0, 0, 0, 0, 0, 0, 0, 0, 0, 0, 0, 0,

0, 0, 0, 0, 0, 0, 0, 0, 0, 0, 0, 0, 0, 0, 0, 0, 0, 0, 0, 0,

0, 0, 0, 0, 0, 0, 0, 0, 0, 0, 0, 0, 0, 0, 0, 0, 0, 0, 0, 0,

0, 0, 0, 0, 0, 0, 0, 0, 0, 0, 0, 0, 0, 0, 0, 0, 0, 0, 0, 0,

0, 0, 0, 0, 0, 0, 0, 0, 0, 0, 0, 0, 0, 0, 0, 0, 0, 0, 0, 0,

0, 0, 0, 0, 0, 0, 0, 0, 0, 0, 0, 0, 0, 0, 0, 0, 0, 0, 0, 0,

0, 0, 0, 0, 0, 0, 0, 0, 0, 0, 0, 0, 0, 0, 0, 0, 0, 0, 0, 0,

0, 0, 0, 0, 0, 0, 0, 0, 0, 0, 0, 0, 0, 0, 0, 0, 0, 0, 0, 0,

0, 0, 0, 0, 0, 0, 0, 0, 0, 0, 0, 0, 0, 0, 0, 0, 0, 0, 0, 0,

0, 0, 0, 0, 0, 0, 0, 0, 0, 0, 0, 0, 0, 0, 0, 0, 0, 0, 0, 0,

0, 0, 0, 0, 0, 0, 0, 0, 0, 0, 0, 0, 0, 0, 0, 0, 0, 0, 0, 0,

0, 0, 0, 0, 0, 0, 0, 0, 0, 0, 0, 0, 0, 0, 0, 0, 0, 0, 0, 0,

0, 0, 0, 0, 0, 0, 0, 0, 0, 0, 0, 0, 0, 0, 0, 0, 0, 0, 0, 0,

0, 0, 0, 0, 0, 0, 0, 0, 0, 0, 0, 0, 0, 0, 0, 0, 0, 0, 0, 0,

0, 0, 0, 0, 0, 0, 0, 0, 0, 0, 0, 0, 0, 0, 0, 0, 0, 0, 0, 0,

0, 0, 0, 0, 0, 0, 0, 0, 0, 0, 0, 0, 0, 0, 0, 0, 0, 0, 0, 0,

0, 0, 0, 0, 0, 0, 0, 0, 0, 0, 0, 0, 0, 0, 0, 0, 0, 0, 0, 0,

0, 0, 0, 0, 0, 0, 0, 0, 0, 0, 0, 0, 0, 0, 0, 0, 0, 0, 0, 0,

0, 0, 0, 0, 0, 0, 0, 0, 0, 0, 0, 0, 0, 0, 0, 0, 0, 0, 0, 0,

0, 0, 0, 0, 0, 0, 0, 0, 0, 0, 0, 0, 0, 0, 0, 0, 0, 0, 0, 0,

0, 0, 0, 0, 0, 0, 0, 0, 0, 0, 0, 0, 0, 0, 0, 0, 0, 0, 0, 0,

0, 0, 0, 0, 0, 0, 0, 0, 0, 0, 0, 0, 0, 0, 0, 0, 0, 0, 0, 0,

0, 0, 0, 0, 0, 0, 0, 0, 0, 0, 0, 0, 0, 0, 0, 0, 0, 0, 0, 0,

0, 0, 0, 0, 0, 0, 0, 0, 0, 0, 0, 0, 0, 0, 0, 0, 0, 0, 0, 0,

0, 0, 0, 0, 0, 0, 0, 0, 0, 0, 0, 0, 0, 0, 0, 0, 0, 0, 0, 0,

0, 0, 0, 0, 0, 0, 0, 0, 0, 0, 0, 0, 0, 0, 0, 0, 0, 0, 0, 0,

0, 0, 0, 0, 0, 0, 0, 0, 0, 0, 0, 0, 0, 0, 0, 0, 0, 0, 0, 0,

0, 0, 0, 0, 0, 0, 0, 0, 0, 0, 0, 0, 0, 0, 0, 0, 0, 0, 0, 0,

0, 0, 0, 0, 0, 0, 0, 0, 0, 0, 0, 0, 0, 0, 0, 0, 0, 0, 0, 0,

0, 0, 0, 0, 0, 0, 0, 0, 0, 0, 0, 0, 0, 0, 0, 0, 0, 0, 0, 0,

0, 0, 0, 0, 0, 0, 0, 0, 0, 0, 0, 0, 0, 0, 0, 0, 0, 0, 0, 0,

0, 0, 0, 0, 0, 0, 0, 0, 0, 0, 0, 0, 0, 0, 0, 0, 0, 0, 0, 0,

0, 0, 0, 0, 0, 0, 0, 0, 0, 0, 0, 0, 0, 0, 0, 0, 0, 0, 0, 0,

0, 0, 0, 0, 0, 0, 0, 0, 0, 0, 0, 0, 0, 0, 0, 0, 0, 0, 0, 0,

0, 0, 0, 0, 0, 0, 0, 0, 0, 0, 0, 0, 0, 0, 0, 0, 0, 0, 0, 0,

0, 0, 0, 0, 0, 0, 0, 0, 0, 0, 0, 0, 0, 0, 0, 0, 0, 0, 0, 0,

0, 0, 0, 0, 0, 0, 0, 0, 0, 0, 0, 0, 0, 0, 0, 0, 0, 0, 0, 0,

0, 0, 0, 0, 0, 0, 0, 0, 0, 0, 0, 0, 0, 0, 0, 0, 0, 0, 0, 0,

0, 0, 0, 0, 0, 0, 0, 0, 0, 0, 0, 0, 0, 0, 0, 0, 0, 0, 0, 0,

0, 0, 0, 0, 0, 0, 0, 0, 0, 0, 0, 0, 0, 0, 0, 0, 0, 0, 0, 0

),.Dim=c(1400,12))

,z=structure(.Data=c(

#H HIC -84

1, 1, 1, 1, 1, 1, 1, 1, 1, 1, 1, 1, 1, 1, 1, 1, 1, 1, 1, 1,

1, 1, 1, 1, 1, 1, 1, 1, 1, 1, 1, 1, 1, 1, 1, 1, 1, 1, 1, 1,

1, 1, 1, 1, 1, 1, 1, 1, 1, 1, 1, 1, 1, 1, 1, 1, 1, 1, 1, 1,

1, 1, 1, 1, 1, 1, 1, 1, 1, 1, 1, 1, 1, 1, 1, 1, 1, 1, 1, 1,

1, 1, 1, 1, 1, 1, 1, 1, 1, 1, 1, 1, 1, 1, 1, 1, 1, 1, 1, 1,

1, 1, 1, 1, 1, 1, 1, 1, 1, 1, 1, 1, 1, 1, 1, 1, 1, 1, 1, 1,

1, 1, 1, 1, 1, 1, 1, 1, 1, 1, 1, 1, 1, 1, 1, 1, 1, 1, 1, 1,

1, 1, 1, 1, 1, 1, 1, 1, 1, 1, 1, 1, 1, 1, 1, 1, 1, 1, 1, 1,

1, 1, 1, 1, 1, 1, 1, 1, 1, 1, 1, 1, 1, 1, 1, 1, 1, 1, 1, 1,

1, 1, 1, 1, 1, 1, 1, 1, 1, 1, 1, 1, 1, 1, 1, 1, 1, 1, 1, 1,

1, 1, 1, 1, 1, 1, 1, 1, 1, 1, 1, 1, 1, 1, 1, 1, 1, 1, 1, 1,

1, 1, 1, 1, 1, 1, 1, 1, 1, 1, 1, 1, 1, 1, 1, 1, 1, 1, 1, 1,

1, 1, 1, 1, 1, 1, 1, 1, 1, 1, 1, 1, 1, 1, 1, 1, 1, 1, 1, 1,

1, 1, 1, 1, 1, 1, 1, 1, 1, 1, 1, 1, 1, 1, 1, 1, 1, 1, 1, 1,

1, 1, 1, 1, 1, 1, 1, 1, 1, 1, 1, 1, 1, 1, 1, 1, 1, 1, 1, 1,

1, 1, 1, 1, 1, 1, 1, 1, 1, 1, 1, 1, 1, 1, 1, 1, 1, 1, 1, 1,

1, 1, 1, 1, 1, 1, 1, 1, 1, 1, 1, 1, 1, 1, 1, 1, 1, 1, 1, 1,

1, 1, 1, 1, 1, 1, 1, 1, 1, 1, 1, 1, 1, 1, 1, 1, 1, 1, 1, 1,

1, 1, 1, 1, 1, 1, 1, 1, 1, 1, 1, 1, 1, 1, 1, 1, 1, 1, 1, 1,

1, 1, 1, 1, 1, 1, 1, 1, 1, 1, 1, 1, 1, 1, 1, 1, 1, 1, 1, 1,

1, 1, 1, 1, 1, 1, 1, 1, 1, 1, 1, 1, 1, 1, 1, 1, 1, 1, 1, 1,

1, 1, 1, 1, 1, 1, 1, 1, 1, 1, 1, 1, 1, 1, 1, 1, 1, 1, 1, 1,

1, 1, 1, 1, 1, 1, 1, 1, 1, 1, 1, 1, 1, 1, 1, 1, 1, 1, 1, 1,

1, 1, 1, 1, 1, 1, 1, 1, 1, 1, 1, 1, 1, 1, 1, 1, 1, 1, 1, 1,

1, 1, 1, 1, 1, 1, 1, 1, 1, 1, 1, 1, 1, 1, 1, 1, 1, 1, 1, 1,

1, 1, 1, 1, 1, 1, 1, 1, 1, 1, 1, 1, 1, 1, 1, 1, 1, 1, 1, 1,

1, 1, 1, 1, 1, 1, 1, 1, 1, 1, 1, 1, 1, 1, 1, 1, 1, 1, 1, 1,

1, 1, 1, 1, 1, 1, 1, 1, 1, 1, 1, 1, 1, 1, 1, 1, 1, 1, 1, 1,

1, 1, 1, 1, 1, 1, 1, 1, 1, 1, 1, 1, 1, 1, 1, 1, 1, 1, 1, 1,

1, 1, 1, 1, 1, 1, 1, 1, 1, 1, 1, 1, 1, 1, 1, 1, 1, 1, 1, 1,

1, 1, 1, 1, 1, 1, 1, 1, 1, 1, 1, 1, 1, 1, 1, 1, 1, 1, 1, 1,

1, 1, 1, 1, 1, NA, NA, NA, NA, NA, NA, NA, NA, NA, NA, NA, NA, NA, NA, NA,

NA, NA, NA, NA, NA, NA, NA, NA, NA, NA, NA, NA, NA, NA, NA, NA, NA, NA, NA, NA,

NA, NA, NA, NA, NA, NA, NA, NA, NA, NA, NA, NA, NA, NA, NA, NA, NA, NA, NA, NA,

NA, NA, NA, NA, NA, NA, NA, NA, NA, NA, NA, NA, NA, NA, NA, NA, NA, NA, NA, NA,

NA, NA, NA, NA, NA, NA, NA, NA, NA, NA, NA, NA, NA, NA, NA, NA, NA, NA, NA, NA,

NA, NA, NA, NA, NA, NA, NA, NA, NA, NA, NA, NA, NA, NA, NA, NA, NA, NA, NA, NA,

NA, NA, NA, NA, NA, NA, NA, NA, NA, NA, NA, NA, NA, NA, NA, NA, NA, NA, NA, NA,

NA, NA, NA, NA, NA, NA, NA, NA, NA, NA, NA, NA, NA, NA, NA, NA, NA, NA, NA, NA,

NA, NA, NA, NA, NA, NA, NA, NA, NA, NA, NA, NA, NA, NA, NA, NA, NA, NA, NA, NA,

NA, NA, NA, NA, NA, NA, NA, NA, NA, NA, NA, NA, NA, NA, NA, NA, NA, NA, NA, NA,

NA, NA, NA, NA, NA, NA, NA, NA, NA, NA, NA, NA, NA, NA, NA, NA, NA, NA, NA, NA,

NA, NA, NA, NA, NA, NA, NA, NA, NA, NA, NA, NA, NA, NA, NA, NA, NA, NA, NA, NA,

NA, NA, NA, NA, NA, NA, NA, NA, NA, NA, NA, NA, NA, NA, NA, NA, NA, NA, NA, NA,

NA, NA, NA, NA, NA, NA, NA, NA, NA, NA, NA, NA, NA, NA, NA, NA, NA, NA, NA, NA,

NA, NA, NA, NA, NA, NA, NA, NA, NA, NA, NA, NA, NA, NA, NA, NA, NA, NA, NA, NA,

NA, NA, NA, NA, NA, NA, NA, NA, NA, NA, NA, NA, NA, NA, NA, NA, NA, NA, NA, NA,

NA, NA, NA, NA, NA, NA, NA, NA, NA, NA, NA, NA, NA, NA, NA, NA, NA, NA, NA, NA,

NA, NA, NA, NA, NA, NA, NA, NA, NA, NA, NA, NA, NA, NA, NA, NA, NA, NA, NA, NA,

NA, NA, NA, NA, NA, NA, NA, NA, NA, NA, NA, NA, NA, NA, NA, NA, NA, NA, NA, NA,

NA, NA, NA, NA, NA, NA, NA, NA, NA, NA, NA, NA, NA, NA, NA, NA, NA, NA, NA, NA,

NA, NA, NA, NA, NA, NA, NA, NA, NA, NA, NA, NA, NA, NA, NA, NA, NA, NA, NA, NA,

NA, NA, NA, NA, NA, NA, NA, NA, NA, NA, NA, NA, NA, NA, NA, NA, NA, NA, NA, NA,

NA, NA, NA, NA, NA, NA, NA, NA, NA, NA, NA, NA, NA, NA, NA, NA, NA, NA, NA, NA,

NA, NA, NA, NA, NA, NA, NA, NA, NA, NA, NA, NA, NA, NA, NA, NA, NA, NA, NA, NA,

NA, NA, NA, NA, NA, NA, NA, NA, NA, NA, NA, NA, NA, NA, NA, NA, NA, NA, NA, NA,

NA, NA, NA, NA, NA, NA, NA, NA, NA, NA, NA, NA, NA, NA, NA, NA, NA, NA, NA, NA,

NA, NA, NA, NA, NA, NA, NA, NA, NA, NA, NA, NA, NA, NA, NA, NA, NA, NA, NA, NA,

NA, NA, NA, NA, NA, NA, NA, NA, NA, NA, NA, NA, NA, NA, NA, NA, NA, NA, NA, NA,

NA, NA, NA, NA, NA, NA, NA, NA, NA, NA, NA, NA, NA, NA, NA, NA, NA, NA, NA, NA,

NA, NA, NA, NA, NA, NA, NA, NA, NA, NA, NA, NA, NA, NA, NA, NA, NA, NA, NA, NA,

NA, NA, NA, NA, NA, NA, NA, NA, NA, NA, NA, NA, NA, NA, NA, NA, NA, NA, NA, NA,

NA, NA, NA, NA, NA, NA, NA, NA, NA, NA, NA, NA, NA, NA, NA, NA, NA, NA, NA, NA,

NA, NA, NA, NA, NA, NA, NA, NA, NA, NA, NA, NA, NA, NA, NA, NA, NA, NA, NA, NA,

NA, NA, NA, NA, NA, NA, NA, NA, NA, NA, NA, NA, NA, NA, NA, NA, NA, NA, NA, NA,

NA, NA, NA, NA, NA, NA, NA, NA, NA, NA, NA, NA, NA, NA, NA, NA, NA, NA, NA, NA,

NA, NA, NA, NA, NA, NA, NA, NA, NA, NA, NA, NA, NA, NA, NA, NA, NA, NA, NA, NA,

NA, NA, NA, NA, NA, NA, NA, NA, NA, NA, NA, NA, NA, NA, NA, NA, NA, NA, NA, NA,

NA, NA, NA, NA, NA, NA, NA, NA, NA, NA, NA, NA, NA, NA, NA, NA, NA, NA, NA, NA,

NA, NA, NA, NA, NA, NA, NA, NA, NA, NA, NA, NA, NA, NA, NA, NA, NA, NA, NA, NA,

#H BNA84-

1, 1, 1, 1, 1, 1, 1, 1, 1, 1, 1, 1, 1, 1, 1, 1, 1, 1, 1, 1,

1, 1, 1, 1, 1, 1, 1, 1, 1, 1, 1, 1, 1, 1, 1, 1, 1, 1, 1, 1,

1, 1, 1, 1, 1, 1, 1, 1, 1, 1, 1, 1, 1, 1, 1, 1, 1, 1, 1, 1,

1, 1, 1, 1, 1, 1, 1, 1, 1, 1, 1, 1, 1, 1, 1, 1, 1, 1, 1, 1,

1, 1, 1, 1, 1, 1, 1, 1, 1, 1, 1, 1, 1, 1, 1, 1, 1, 1, 1, 1,

1, 1, 1, 1, 1, 1, 1, 1, 1, 1, 1, 1, 1, 1, 1, 1, 1, 1, 1, 1,

1, 1, 1, 1, 1, 1, 1, 1, 1, 1, 1, 1, 1, 1, 1, 1, 1, 1, 1, 1,

1, 1, 1, 1, 1, 1, 1, 1, 1, 1, 1, 1, 1, 1, 1, 1, 1, 1, 1, 1,

1, 1, 1, 1, 1, 1, 1, 1, 1, 1, 1, 1, 1, 1, 1, 1, 1, 1, 1, 1,

1, 1, 1, 1, 1, 1, 1, 1, 1, 1, 1, 1, 1, 1, 1, 1, 1, 1, 1, 1,

1, 1, 1, 1, 1, 1, 1, 1, 1, 1, 1, 1, 1, 1, 1, 1, 1, 1, 1, 1,

1, 1, 1, 1, 1, 1, 1, 1, 1, 1, 1, 1, 1, 1, 1, 1, 1, 1, 1, 1,

1, 1, 1, 1, 1, 1, 1, 1, 1, 1, 1, 1, 1, 1, 1, 1, 1, 1, 1, 1,

1, 1, 1, 1, 1, 1, 1, 1, 1, 1, 1, 1, 1, 1, 1, 1, 1, 1, 1, 1,

1, 1, 1, 1, 1, 1, 1, 1, 1, 1, 1, 1, 1, 1, 1, 1, 1, 1, 1, 1,

1, 1, 1, 1, 1, 1, 1, 1, 1, 1, 1, 1, 1, 1, 1, 1, 1, 1, 1, 1,

1, 1, 1, 1, 1, 1, 1, 1, 1, 1, 1, 1, 1, 1, 1, 1, 1, 1, 1, 1,

1, 1, 1, 1, 1, 1, 1, 1, 1, 1, 1, 1, 1, 1, 1, 1, 1, 1, 1, 1,

1, 1, 1, 1, 1, 1, 1, 1, 1, 1, 1, 1, 1, 1, 1, 1, 1, 1, 1, 1,

1, 1, 1, 1, 1, 1, 1, 1, 1, 1, 1, 1, 1, 1, 1, 1, 1, 1, 1, 1,

1, 1, 1, 1, 1, 1, 1, 1, 1, 1, 1, 1, 1, 1, 1, 1, 1, 1, 1, 1,

1, 1, 1, 1, 1, 1, 1, 1, 1, 1, 1, 1, 1, 1, 1, 1, 1, 1, 1, 1,

1, 1, 1, 1, 1, 1, 1, 1, 1, 1, 1, 1, 1, 1, 1, 1, 1, 1, 1, 1,

1, 1, 1, 1, 1, 1, 1, 1, 1, 1, 1, 1, 1, 1, 1, 1, 1, 1, 1, 1,

1, 1, 1, 1, 1, 1, 1, 1, 1, 1, 1, 1, 1, 1, 1, 1, NA, NA, NA, NA,

NA, NA, NA, NA, NA, NA, NA, NA, NA, NA, NA, NA, NA, NA, NA, NA, NA, NA, NA, NA,

NA, NA, NA, NA, NA, NA, NA, NA, NA, NA, NA, NA, NA, NA, NA, NA, NA, NA, NA, NA,

NA, NA, NA, NA, NA, NA, NA, NA, NA, NA, NA, NA, NA, NA, NA, NA, NA, NA, NA, NA,

NA, NA, NA, NA, NA, NA, NA, NA, NA, NA, NA, NA, NA, NA, NA, NA, NA, NA, NA, NA,

NA, NA, NA, NA, NA, NA, NA, NA, NA, NA, NA, NA, NA, NA, NA, NA, NA, NA, NA, NA,

NA, NA, NA, NA, NA, NA, NA, NA, NA, NA, NA, NA, NA, NA, NA, NA, NA, NA, NA, NA,

NA, NA, NA, NA, NA, NA, NA, NA, NA, NA, NA, NA, NA, NA, NA, NA, NA, NA, NA, NA,

NA, NA, NA, NA, NA, NA, NA, NA, NA, NA, NA, NA, NA, NA, NA, NA, NA, NA, NA, NA,

NA, NA, NA, NA, NA, NA, NA, NA, NA, NA, NA, NA, NA, NA, NA, NA, NA, NA, NA, NA,

NA, NA, NA, NA, NA, NA, NA, NA, NA, NA, NA, NA, NA, NA, NA, NA, NA, NA, NA, NA,

NA, NA, NA, NA, NA, NA, NA, NA, NA, NA, NA, NA, NA, NA, NA, NA, NA, NA, NA, NA,

NA, NA, NA, NA, NA, NA, NA, NA, NA, NA, NA, NA, NA, NA, NA, NA, NA, NA, NA, NA,

NA, NA, NA, NA, NA, NA, NA, NA, NA, NA, NA, NA, NA, NA, NA, NA, NA, NA, NA, NA,

NA, NA, NA, NA, NA, NA, NA, NA, NA, NA, NA, NA, NA, NA, NA, NA, NA, NA, NA, NA,

NA, NA, NA, NA, NA, NA, NA, NA, NA, NA, NA, NA, NA, NA, NA, NA, NA, NA, NA, NA,

NA, NA, NA, NA, NA, NA, NA, NA, NA, NA, NA, NA, NA, NA, NA, NA, NA, NA, NA, NA,

NA, NA, NA, NA, NA, NA, NA, NA, NA, NA, NA, NA, NA, NA, NA, NA, NA, NA, NA, NA,

NA, NA, NA, NA, NA, NA, NA, NA, NA, NA, NA, NA, NA, NA, NA, NA, NA, NA, NA, NA,

NA, NA, NA, NA, NA, NA, NA, NA, NA, NA, NA, NA, NA, NA, NA, NA, NA, NA, NA, NA,

NA, NA, NA, NA, NA, NA, NA, NA, NA, NA, NA, NA, NA, NA, NA, NA, NA, NA, NA, NA,

NA, NA, NA, NA, NA, NA, NA, NA, NA, NA, NA, NA, NA, NA, NA, NA, NA, NA, NA, NA,

NA, NA, NA, NA, NA, NA, NA, NA, NA, NA, NA, NA, NA, NA, NA, NA, NA, NA, NA, NA,

NA, NA, NA, NA, NA, NA, NA, NA, NA, NA, NA, NA, NA, NA, NA, NA, NA, NA, NA, NA,

NA, NA, NA, NA, NA, NA, NA, NA, NA, NA, NA, NA, NA, NA, NA, NA, NA, NA, NA, NA,

NA, NA, NA, NA, NA, NA, NA, NA, NA, NA, NA, NA, NA, NA, NA, NA, NA, NA, NA, NA,

NA, NA, NA, NA, NA, NA, NA, NA, NA, NA, NA, NA, NA, NA, NA, NA, NA, NA, NA, NA,

NA, NA, NA, NA, NA, NA, NA, NA, NA, NA, NA, NA, NA, NA, NA, NA, NA, NA, NA, NA,

NA, NA, NA, NA, NA, NA, NA, NA, NA, NA, NA, NA, NA, NA, NA, NA, NA, NA, NA, NA,

NA, NA, NA, NA, NA, NA, NA, NA, NA, NA, NA, NA, NA, NA, NA, NA, NA, NA, NA, NA,

NA, NA, NA, NA, NA, NA, NA, NA, NA, NA, NA, NA, NA, NA, NA, NA, NA, NA, NA, NA,

NA, NA, NA, NA, NA, NA, NA, NA, NA, NA, NA, NA, NA, NA, NA, NA, NA, NA, NA, NA,

NA, NA, NA, NA, NA, NA, NA, NA, NA, NA, NA, NA, NA, NA, NA, NA, NA, NA, NA, NA,

NA, NA, NA, NA, NA, NA, NA, NA, NA, NA, NA, NA, NA, NA, NA, NA, NA, NA, NA, NA,

NA, NA, NA, NA, NA, NA, NA, NA, NA, NA, NA, NA, NA, NA, NA, NA, NA, NA, NA, NA,

NA, NA, NA, NA, NA, NA, NA, NA, NA, NA, NA, NA, NA, NA, NA, NA, NA, NA, NA, NA,

NA, NA, NA, NA, NA, NA, NA, NA, NA, NA, NA, NA, NA, NA, NA, NA, NA, NA, NA, NA,

NA, NA, NA, NA, NA, NA, NA, NA, NA, NA, NA, NA, NA, NA, NA, NA, NA, NA, NA, NA,

NA, NA, NA, NA, NA, NA, NA, NA, NA, NA, NA, NA, NA, NA, NA, NA, NA, NA, NA, NA,

NA, NA, NA, NA, NA, NA, NA, NA, NA, NA, NA, NA, NA, NA, NA, NA, NA, NA, NA, NA,

NA, NA, NA, NA, NA, NA, NA, NA, NA, NA, NA, NA, NA, NA, NA, NA, NA, NA, NA, NA,

NA, NA, NA, NA, NA, NA, NA, NA, NA, NA, NA, NA, NA, NA, NA, NA, NA, NA, NA, NA,

NA, NA, NA, NA, NA, NA, NA, NA, NA, NA, NA, NA, NA, NA, NA, NA, NA, NA, NA, NA,

NA, NA, NA, NA, NA, NA, NA, NA, NA, NA, NA, NA, NA, NA, NA, NA, NA, NA, NA, NA,

NA, NA, NA, NA, NA, NA, NA, NA, NA, NA, NA, NA, NA, NA, NA, NA, NA, NA, NA, NA,

NA, NA, NA, NA, NA, NA, NA, NA, NA, NA, NA, NA, NA, NA, NA, NA, NA, NA, NA, NA,

#H HIC85-89

1, 1, 1, 1, 1, 1, 1, 1, 1, 1, 1, 1, 1, 1, 1, 1, 1, 1, 1, 1,

1, 1, 1, 1, 1, 1, 1, 1, 1, 1, 1, 1, 1, 1, 1, 1, 1, 1, 1, 1,

1, 1, 1, 1, 1, 1, 1, 1, 1, 1, 1, 1, 1, 1, 1, 1, 1, 1, 1, 1,

1, 1, 1, 1, 1, 1, 1, 1, 1, 1, 1, 1, 1, 1, 1, 1, 1, 1, 1, 1,

1, 1, 1, 1, 1, 1, 1, 1, 1, 1, 1, 1, 1, 1, 1, 1, 1, 1, 1, 1,

1, 1, 1, 1, 1, 1, 1, 1, 1, 1, 1, 1, 1, 1, 1, 1, 1, 1, 1, 1,

1, 1, 1, 1, 1, 1, 1, 1, 1, 1, 1, 1, 1, 1, 1, 1, 1, 1, 1, 1,

1, 1, 1, 1, 1, 1, 1, 1, 1, 1, 1, 1, 1, 1, 1, 1, 1, 1, 1, 1,

1, 1, 1, 1, 1, 1, 1, 1, 1, 1, 1, 1, 1, 1, 1, 1, 1, 1, 1, 1,

1, 1, 1, 1, 1, 1, 1, 1, 1, 1, 1, 1, 1, 1, 1, 1, 1, 1, 1, 1,

1, 1, 1, 1, 1, 1, 1, 1, 1, 1, 1, 1, 1, 1, 1, 1, 1, 1, 1, 1,

1, 1, 1, 1, 1, 1, 1, 1, 1, NA, NA, NA, NA, NA, NA, NA, NA, NA, NA, NA,

NA, NA, NA, NA, NA, NA, NA, NA, NA, NA, NA, NA, NA, NA, NA, NA, NA, NA, NA, NA,

NA, NA, NA, NA, NA, NA, NA, NA, NA, NA, NA, NA, NA, NA, NA, NA, NA, NA, NA, NA,

NA, NA, NA, NA, NA, NA, NA, NA, NA, NA, NA, NA, NA, NA, NA, NA, NA, NA, NA, NA,

NA, NA, NA, NA, NA, NA, NA, NA, NA, NA, NA, NA, NA, NA, NA, NA, NA, NA, NA, NA,

NA, NA, NA, NA, NA, NA, NA, NA, NA, NA, NA, NA, NA, NA, NA, NA, NA, NA, NA, NA,

NA, NA, NA, NA, NA, NA, NA, NA, NA, NA, NA, NA, NA, NA, NA, NA, NA, NA, NA, NA,

NA, NA, NA, NA, NA, NA, NA, NA, NA, NA, NA, NA, NA, NA, NA, NA, NA, NA, NA, NA,

NA, NA, NA, NA, NA, NA, NA, NA, NA, NA, NA, NA, NA, NA, NA, NA, NA, NA, NA, NA,

NA, NA, NA, NA, NA, NA, NA, NA, NA, NA, NA, NA, NA, NA, NA, NA, NA, NA, NA, NA,

NA, NA, NA, NA, NA, NA, NA, NA, NA, NA, NA, NA, NA, NA, NA, NA, NA, NA, NA, NA,

NA, NA, NA, NA, NA, NA, NA, NA, NA, NA, NA, NA, NA, NA, NA, NA, NA, NA, NA, NA,

NA, NA, NA, NA, NA, NA, NA, NA, NA, NA, NA, NA, NA, NA, NA, NA, NA, NA, NA, NA,

NA, NA, NA, NA, NA, NA, NA, NA, NA, NA, NA, NA, NA, NA, NA, NA, NA, NA, NA, NA,

NA, NA, NA, NA, NA, NA, NA, NA, NA, NA, NA, NA, NA, NA, NA, NA, NA, NA, NA, NA,

NA, NA, NA, NA, NA, NA, NA, NA, NA, NA, NA, NA, NA, NA, NA, NA, NA, NA, NA, NA,

NA, NA, NA, NA, NA, NA, NA, NA, NA, NA, NA, NA, NA, NA, NA, NA, NA, NA, NA, NA,

NA, NA, NA, NA, NA, NA, NA, NA, NA, NA, NA, NA, NA, NA, NA, NA, NA, NA, NA, NA,

NA, NA, NA, NA, NA, NA, NA, NA, NA, NA, NA, NA, NA, NA, NA, NA, NA, NA, NA, NA,

NA, NA, NA, NA, NA, NA, NA, NA, NA, NA, NA, NA, NA, NA, NA, NA, NA, NA, NA, NA,

NA, NA, NA, NA, NA, NA, NA, NA, NA, NA, NA, NA, NA, NA, NA, NA, NA, NA, NA, NA,

NA, NA, NA, NA, NA, NA, NA, NA, NA, NA, NA, NA, NA, NA, NA, NA, NA, NA, NA, NA,

NA, NA, NA, NA, NA, NA, NA, NA, NA, NA, NA, NA, NA, NA, NA, NA, NA, NA, NA, NA,

NA, NA, NA, NA, NA, NA, NA, NA, NA, NA, NA, NA, NA, NA, NA, NA, NA, NA, NA, NA,

NA, NA, NA, NA, NA, NA, NA, NA, NA, NA, NA, NA, NA, NA, NA, NA, NA, NA, NA, NA,

NA, NA, NA, NA, NA, NA, NA, NA, NA, NA, NA, NA, NA, NA, NA, NA, NA, NA, NA, NA,

NA, NA, NA, NA, NA, NA, NA, NA, NA, NA, NA, NA, NA, NA, NA, NA, NA, NA, NA, NA,

NA, NA, NA, NA, NA, NA, NA, NA, NA, NA, NA, NA, NA, NA, NA, NA, NA, NA, NA, NA,

NA, NA, NA, NA, NA, NA, NA, NA, NA, NA, NA, NA, NA, NA, NA, NA, NA, NA, NA, NA,

NA, NA, NA, NA, NA, NA, NA, NA, NA, NA, NA, NA, NA, NA, NA, NA, NA, NA, NA, NA,

NA, NA, NA, NA, NA, NA, NA, NA, NA, NA, NA, NA, NA, NA, NA, NA, NA, NA, NA, NA,

NA, NA, NA, NA, NA, NA, NA, NA, NA, NA, NA, NA, NA, NA, NA, NA, NA, NA, NA, NA,

NA, NA, NA, NA, NA, NA, NA, NA, NA, NA, NA, NA, NA, NA, NA, NA, NA, NA, NA, NA,

NA, NA, NA, NA, NA, NA, NA, NA, NA, NA, NA, NA, NA, NA, NA, NA, NA, NA, NA, NA,

NA, NA, NA, NA, NA, NA, NA, NA, NA, NA, NA, NA, NA, NA, NA, NA, NA, NA, NA, NA,

NA, NA, NA, NA, NA, NA, NA, NA, NA, NA, NA, NA, NA, NA, NA, NA, NA, NA, NA, NA,

NA, NA, NA, NA, NA, NA, NA, NA, NA, NA, NA, NA, NA, NA, NA, NA, NA, NA, NA, NA,

NA, NA, NA, NA, NA, NA, NA, NA, NA, NA, NA, NA, NA, NA, NA, NA, NA, NA, NA, NA,

NA, NA, NA, NA, NA, NA, NA, NA, NA, NA, NA, NA, NA, NA, NA, NA, NA, NA, NA, NA,

NA, NA, NA, NA, NA, NA, NA, NA, NA, NA, NA, NA, NA, NA, NA, NA, NA, NA, NA, NA,

NA, NA, NA, NA, NA, NA, NA, NA, NA, NA, NA, NA, NA, NA, NA, NA, NA, NA, NA, NA,

NA, NA, NA, NA, NA, NA, NA, NA, NA, NA, NA, NA, NA, NA, NA, NA, NA, NA, NA, NA,

NA, NA, NA, NA, NA, NA, NA, NA, NA, NA, NA, NA, NA, NA, NA, NA, NA, NA, NA, NA,

NA, NA, NA, NA, NA, NA, NA, NA, NA, NA, NA, NA, NA, NA, NA, NA, NA, NA, NA, NA,

NA, NA, NA, NA, NA, NA, NA, NA, NA, NA, NA, NA, NA, NA, NA, NA, NA, NA, NA, NA,

NA, NA, NA, NA, NA, NA, NA, NA, NA, NA, NA, NA, NA, NA, NA, NA, NA, NA, NA, NA,

NA, NA, NA, NA, NA, NA, NA, NA, NA, NA, NA, NA, NA, NA, NA, NA, NA, NA, NA, NA,

NA, NA, NA, NA, NA, NA, NA, NA, NA, NA, NA, NA, NA, NA, NA, NA, NA, NA, NA, NA,

NA, NA, NA, NA, NA, NA, NA, NA, NA, NA, NA, NA, NA, NA, NA, NA, NA, NA, NA, NA,

NA, NA, NA, NA, NA, NA, NA, NA, NA, NA, NA, NA, NA, NA, NA, NA, NA, NA, NA, NA,

NA, NA, NA, NA, NA, NA, NA, NA, NA, NA, NA, NA, NA, NA, NA, NA, NA, NA, NA, NA,

NA, NA, NA, NA, NA, NA, NA, NA, NA, NA, NA, NA, NA, NA, NA, NA, NA, NA, NA, NA,

NA, NA, NA, NA, NA, NA, NA, NA, NA, NA, NA, NA, NA, NA, NA, NA, NA, NA, NA, NA,

NA, NA, NA, NA, NA, NA, NA, NA, NA, NA, NA, NA, NA, NA, NA, NA, NA, NA, NA, NA,

NA, NA, NA, NA, NA, NA, NA, NA, NA, NA, NA, NA, NA, NA, NA, NA, NA, NA, NA, NA,

NA, NA, NA, NA, NA, NA, NA, NA, NA, NA, NA, NA, NA, NA, NA, NA, NA, NA, NA, NA,

NA, NA, NA, NA, NA, NA, NA, NA, NA, NA, NA, NA, NA, NA, NA, NA, NA, NA, NA, NA,

NA, NA, NA, NA, NA, NA, NA, NA, NA, NA, NA, NA, NA, NA, NA, NA, NA, NA, NA, NA,

NA, NA, NA, NA, NA, NA, NA, NA, NA, NA, NA, NA, NA, NA, NA, NA, NA, NA, NA, NA,

#H BNA85-90A

1, 1, 1, 1, 1, 1, 1, 1, 1, 1, 1, 1, 1, 1, 1, 1, 1, 1, 1, 1,

1, 1, 1, 1, 1, 1, 1, 1, 1, 1, 1, 1, 1, 1, 1, 1, 1, 1, 1, 1,

1, 1, 1, 1, 1, 1, 1, 1, 1, 1, 1, 1, 1, 1, 1, 1, 1, 1, 1, 1,

1, 1, 1, 1, 1, 1, 1, 1, 1, 1, 1, 1, 1, 1, 1, 1, 1, 1, 1, 1,

1, 1, 1, 1, 1, 1, 1, 1, 1, 1, 1, 1, 1, 1, 1, 1, 1, 1, 1, 1,

1, 1, 1, 1, 1, 1, 1, 1, 1, 1, 1, 1, 1, 1, 1, 1, 1, 1, 1, 1,

1, 1, 1, 1, 1, 1, 1, 1, 1, 1, 1, 1, 1, 1, 1, 1, 1, 1, 1, 1,

1, 1, 1, 1, 1, 1, 1, 1, 1, 1, 1, 1, 1, 1, 1, 1, 1, 1, 1, 1,

1, 1, 1, 1, 1, 1, 1, 1, 1, 1, 1, 1, 1, NA, NA, NA, NA, NA, NA, NA,

NA, NA, NA, NA, NA, NA, NA, NA, NA, NA, NA, NA, NA, NA, NA, NA, NA, NA, NA, NA,

NA, NA, NA, NA, NA, NA, NA, NA, NA, NA, NA, NA, NA, NA, NA, NA, NA, NA, NA, NA,

NA, NA, NA, NA, NA, NA, NA, NA, NA, NA, NA, NA, NA, NA, NA, NA, NA, NA, NA, NA,

NA, NA, NA, NA, NA, NA, NA, NA, NA, NA, NA, NA, NA, NA, NA, NA, NA, NA, NA, NA,

NA, NA, NA, NA, NA, NA, NA, NA, NA, NA, NA, NA, NA, NA, NA, NA, NA, NA, NA, NA,

NA, NA, NA, NA, NA, NA, NA, NA, NA, NA, NA, NA, NA, NA, NA, NA, NA, NA, NA, NA,

NA, NA, NA, NA, NA, NA, NA, NA, NA, NA, NA, NA, NA, NA, NA, NA, NA, NA, NA, NA,

NA, NA, NA, NA, NA, NA, NA, NA, NA, NA, NA, NA, NA, NA, NA, NA, NA, NA, NA, NA,

NA, NA, NA, NA, NA, NA, NA, NA, NA, NA, NA, NA, NA, NA, NA, NA, NA, NA, NA, NA,

NA, NA, NA, NA, NA, NA, NA, NA, NA, NA, NA, NA, NA, NA, NA, NA, NA, NA, NA, NA,

NA, NA, NA, NA, NA, NA, NA, NA, NA, NA, NA, NA, NA, NA, NA, NA, NA, NA, NA, NA,

NA, NA, NA, NA, NA, NA, NA, NA, NA, NA, NA, NA, NA, NA, NA, NA, NA, NA, NA, NA,

NA, NA, NA, NA, NA, NA, NA, NA, NA, NA, NA, NA, NA, NA, NA, NA, NA, NA, NA, NA,

NA, NA, NA, NA, NA, NA, NA, NA, NA, NA, NA, NA, NA, NA, NA, NA, NA, NA, NA, NA,

NA, NA, NA, NA, NA, NA, NA, NA, NA, NA, NA, NA, NA, NA, NA, NA, NA, NA, NA, NA,

NA, NA, NA, NA, NA, NA, NA, NA, NA, NA, NA, NA, NA, NA, NA, NA, NA, NA, NA, NA,

NA, NA, NA, NA, NA, NA, NA, NA, NA, NA, NA, NA, NA, NA, NA, NA, NA, NA, NA, NA,

NA, NA, NA, NA, NA, NA, NA, NA, NA, NA, NA, NA, NA, NA, NA, NA, NA, NA, NA, NA,

NA, NA, NA, NA, NA, NA, NA, NA, NA, NA, NA, NA, NA, NA, NA, NA, NA, NA, NA, NA,

NA, NA, NA, NA, NA, NA, NA, NA, NA, NA, NA, NA, NA, NA, NA, NA, NA, NA, NA, NA,

NA, NA, NA, NA, NA, NA, NA, NA, NA, NA, NA, NA, NA, NA, NA, NA, NA, NA, NA, NA,

NA, NA, NA, NA, NA, NA, NA, NA, NA, NA, NA, NA, NA, NA, NA, NA, NA, NA, NA, NA,

NA, NA, NA, NA, NA, NA, NA, NA, NA, NA, NA, NA, NA, NA, NA, NA, NA, NA, NA, NA,

NA, NA, NA, NA, NA, NA, NA, NA, NA, NA, NA, NA, NA, NA, NA, NA, NA, NA, NA, NA,

NA, NA, NA, NA, NA, NA, NA, NA, NA, NA, NA, NA, NA, NA, NA, NA, NA, NA, NA, NA,

NA, NA, NA, NA, NA, NA, NA, NA, NA, NA, NA, NA, NA, NA, NA, NA, NA, NA, NA, NA,

NA, NA, NA, NA, NA, NA, NA, NA, NA, NA, NA, NA, NA, NA, NA, NA, NA, NA, NA, NA,

NA, NA, NA, NA, NA, NA, NA, NA, NA, NA, NA, NA, NA, NA, NA, NA, NA, NA, NA, NA,

NA, NA, NA, NA, NA, NA, NA, NA, NA, NA, NA, NA, NA, NA, NA, NA, NA, NA, NA, NA,

NA, NA, NA, NA, NA, NA, NA, NA, NA, NA, NA, NA, NA, NA, NA, NA, NA, NA, NA, NA,

NA, NA, NA, NA, NA, NA, NA, NA, NA, NA, NA, NA, NA, NA, NA, NA, NA, NA, NA, NA,

NA, NA, NA, NA, NA, NA, NA, NA, NA, NA, NA, NA, NA, NA, NA, NA, NA, NA, NA, NA,

NA, NA, NA, NA, NA, NA, NA, NA, NA, NA, NA, NA, NA, NA, NA, NA, NA, NA, NA, NA,

NA, NA, NA, NA, NA, NA, NA, NA, NA, NA, NA, NA, NA, NA, NA, NA, NA, NA, NA, NA,

NA, NA, NA, NA, NA, NA, NA, NA, NA, NA, NA, NA, NA, NA, NA, NA, NA, NA, NA, NA,

NA, NA, NA, NA, NA, NA, NA, NA, NA, NA, NA, NA, NA, NA, NA, NA, NA, NA, NA, NA,

NA, NA, NA, NA, NA, NA, NA, NA, NA, NA, NA, NA, NA, NA, NA, NA, NA, NA, NA, NA,

NA, NA, NA, NA, NA, NA, NA, NA, NA, NA, NA, NA, NA, NA, NA, NA, NA, NA, NA, NA,

NA, NA, NA, NA, NA, NA, NA, NA, NA, NA, NA, NA, NA, NA, NA, NA, NA, NA, NA, NA,

NA, NA, NA, NA, NA, NA, NA, NA, NA, NA, NA, NA, NA, NA, NA, NA, NA, NA, NA, NA,

NA, NA, NA, NA, NA, NA, NA, NA, NA, NA, NA, NA, NA, NA, NA, NA, NA, NA, NA, NA,

NA, NA, NA, NA, NA, NA, NA, NA, NA, NA, NA, NA, NA, NA, NA, NA, NA, NA, NA, NA,

NA, NA, NA, NA, NA, NA, NA, NA, NA, NA, NA, NA, NA, NA, NA, NA, NA, NA, NA, NA,

NA, NA, NA, NA, NA, NA, NA, NA, NA, NA, NA, NA, NA, NA, NA, NA, NA, NA, NA, NA,

NA, NA, NA, NA, NA, NA, NA, NA, NA, NA, NA, NA, NA, NA, NA, NA, NA, NA, NA, NA,

NA, NA, NA, NA, NA, NA, NA, NA, NA, NA, NA, NA, NA, NA, NA, NA, NA, NA, NA, NA,

NA, NA, NA, NA, NA, NA, NA, NA, NA, NA, NA, NA, NA, NA, NA, NA, NA, NA, NA, NA,

NA, NA, NA, NA, NA, NA, NA, NA, NA, NA, NA, NA, NA, NA, NA, NA, NA, NA, NA, NA,

NA, NA, NA, NA, NA, NA, NA, NA, NA, NA, NA, NA, NA, NA, NA, NA, NA, NA, NA, NA,

NA, NA, NA, NA, NA, NA, NA, NA, NA, NA, NA, NA, NA, NA, NA, NA, NA, NA, NA, NA,

NA, NA, NA, NA, NA, NA, NA, NA, NA, NA, NA, NA, NA, NA, NA, NA, NA, NA, NA, NA,

NA, NA, NA, NA, NA, NA, NA, NA, NA, NA, NA, NA, NA, NA, NA, NA, NA, NA, NA, NA,

NA, NA, NA, NA, NA, NA, NA, NA, NA, NA, NA, NA, NA, NA, NA, NA, NA, NA, NA, NA,

NA, NA, NA, NA, NA, NA, NA, NA, NA, NA, NA, NA, NA, NA, NA, NA, NA, NA, NA, NA,

NA, NA, NA, NA, NA, NA, NA, NA, NA, NA, NA, NA, NA, NA, NA, NA, NA, NA, NA, NA,

NA, NA, NA, NA, NA, NA, NA, NA, NA, NA, NA, NA, NA, NA, NA, NA, NA, NA, NA, NA,

NA, NA, NA, NA, NA, NA, NA, NA, NA, NA, NA, NA, NA, NA, NA, NA, NA, NA, NA, NA,

NA, NA, NA, NA, NA, NA, NA, NA, NA, NA, NA, NA, NA, NA, NA, NA, NA, NA, NA, NA,

NA, NA, NA, NA, NA, NA, NA, NA, NA, NA, NA, NA, NA, NA, NA, NA, NA, NA, NA, NA,

NA, NA, NA, NA, NA, NA, NA, NA, NA, NA, NA, NA, NA, NA, NA, NA, NA, NA, NA, NA,

NA, NA, NA, NA, NA, NA, NA, NA, NA, NA, NA, NA, NA, NA, NA, NA, NA, NA, NA, NA,

#H HIC 90+

1, 1, 1, 1, 1, 1, 1, 1, 1, 1, 1, 1, 1, 1, 1, 1, 1, 1, 1, 1,

1, 1, 1, 1, 1, 1, 1, 1, 1, 1, 1, 1, 1, 1, 1, 1, 1, 1, 1, 1,

1, 1, 1, 1, 1, 1, 1, 1, 1, 1, 1, 1, 1, 1, 1, 1, 1, 1, 1, 1,

1, 1, 1, 1, 1, 1, 1, 1, 1, 1, 1, 1, 1, 1, 1, 1, 1, 1, 1, 1,

1, 1, 1, 1, 1, 1, 1, 1, 1, 1, 1, 1, NA, NA, NA, NA, NA, NA, NA, NA,

NA, NA, NA, NA, NA, NA, NA, NA, NA, NA, NA, NA, NA, NA, NA, NA, NA, NA, NA, NA,

NA, NA, NA, NA, NA, NA, NA, NA, NA, NA, NA, NA, NA, NA, NA, NA, NA, NA, NA, NA,

NA, NA, NA, NA, NA, NA, NA, NA, NA, NA, NA, NA, NA, NA, NA, NA, NA, NA, NA, NA,

NA, NA, NA, NA, NA, NA, NA, NA, NA, NA, NA, NA, NA, NA, NA, NA, NA, NA, NA, NA,

NA, NA, NA, NA, NA, NA, NA, NA, NA, NA, NA, NA, NA, NA, NA, NA, NA, NA, NA, NA,

NA, NA, NA, NA, NA, NA, NA, NA, NA, NA, NA, NA, NA, NA, NA, NA, NA, NA, NA, NA,

NA, NA, NA, NA, NA, NA, NA, NA, NA, NA, NA, NA, NA, NA, NA, NA, NA, NA, NA, NA,

NA, NA, NA, NA, NA, NA, NA, NA, NA, NA, NA, NA, NA, NA, NA, NA, NA, NA, NA, NA,

NA, NA, NA, NA, NA, NA, NA, NA, NA, NA, NA, NA, NA, NA, NA, NA, NA, NA, NA, NA,

NA, NA, NA, NA, NA, NA, NA, NA, NA, NA, NA, NA, NA, NA, NA, NA, NA, NA, NA, NA,

NA, NA, NA, NA, NA, NA, NA, NA, NA, NA, NA, NA, NA, NA, NA, NA, NA, NA, NA, NA,

NA, NA, NA, NA, NA, NA, NA, NA, NA, NA, NA, NA, NA, NA, NA, NA, NA, NA, NA, NA,

NA, NA, NA, NA, NA, NA, NA, NA, NA, NA, NA, NA, NA, NA, NA, NA, NA, NA, NA, NA,

NA, NA, NA, NA, NA, NA, NA, NA, NA, NA, NA, NA, NA, NA, NA, NA, NA, NA, NA, NA,

NA, NA, NA, NA, NA, NA, NA, NA, NA, NA, NA, NA, NA, NA, NA, NA, NA, NA, NA, NA,

NA, NA, NA, NA, NA, NA, NA, NA, NA, NA, NA, NA, NA, NA, NA, NA, NA, NA, NA, NA,

NA, NA, NA, NA, NA, NA, NA, NA, NA, NA, NA, NA, NA, NA, NA, NA, NA, NA, NA, NA,

NA, NA, NA, NA, NA, NA, NA, NA, NA, NA, NA, NA, NA, NA, NA, NA, NA, NA, NA, NA,

NA, NA, NA, NA, NA, NA, NA, NA, NA, NA, NA, NA, NA, NA, NA, NA, NA, NA, NA, NA,

NA, NA, NA, NA, NA, NA, NA, NA, NA, NA, NA, NA, NA, NA, NA, NA, NA, NA, NA, NA,

NA, NA, NA, NA, NA, NA, NA, NA, NA, NA, NA, NA, NA, NA, NA, NA, NA, NA, NA, NA,

NA, NA, NA, NA, NA, NA, NA, NA, NA, NA, NA, NA, NA, NA, NA, NA, NA, NA, NA, NA,

NA, NA, NA, NA, NA, NA, NA, NA, NA, NA, NA, NA, NA, NA, NA, NA, NA, NA, NA, NA,

NA, NA, NA, NA, NA, NA, NA, NA, NA, NA, NA, NA, NA, NA, NA, NA, NA, NA, NA, NA,

NA, NA, NA, NA, NA, NA, NA, NA, NA, NA, NA, NA, NA, NA, NA, NA, NA, NA, NA, NA,

NA, NA, NA, NA, NA, NA, NA, NA, NA, NA, NA, NA, NA, NA, NA, NA, NA, NA, NA, NA,

NA, NA, NA, NA, NA, NA, NA, NA, NA, NA, NA, NA, NA, NA, NA, NA, NA, NA, NA, NA,

NA, NA, NA, NA, NA, NA, NA, NA, NA, NA, NA, NA, NA, NA, NA, NA, NA, NA, NA, NA,

NA, NA, NA, NA, NA, NA, NA, NA, NA, NA, NA, NA, NA, NA, NA, NA, NA, NA, NA, NA,

NA, NA, NA, NA, NA, NA, NA, NA, NA, NA, NA, NA, NA, NA, NA, NA, NA, NA, NA, NA,

NA, NA, NA, NA, NA, NA, NA, NA, NA, NA, NA, NA, NA, NA, NA, NA, NA, NA, NA, NA,

NA, NA, NA, NA, NA, NA, NA, NA, NA, NA, NA, NA, NA, NA, NA, NA, NA, NA, NA, NA,

NA, NA, NA, NA, NA, NA, NA, NA, NA, NA, NA, NA, NA, NA, NA, NA, NA, NA, NA, NA,

NA, NA, NA, NA, NA, NA, NA, NA, NA, NA, NA, NA, NA, NA, NA, NA, NA, NA, NA, NA,

NA, NA, NA, NA, NA, NA, NA, NA, NA, NA, NA, NA, NA, NA, NA, NA, NA, NA, NA, NA,

NA, NA, NA, NA, NA, NA, NA, NA, NA, NA, NA, NA, NA, NA, NA, NA, NA, NA, NA, NA,

NA, NA, NA, NA, NA, NA, NA, NA, NA, NA, NA, NA, NA, NA, NA, NA, NA, NA, NA, NA,

NA, NA, NA, NA, NA, NA, NA, NA, NA, NA, NA, NA, NA, NA, NA, NA, NA, NA, NA, NA,

NA, NA, NA, NA, NA, NA, NA, NA, NA, NA, NA, NA, NA, NA, NA, NA, NA, NA, NA, NA,

NA, NA, NA, NA, NA, NA, NA, NA, NA, NA, NA, NA, NA, NA, NA, NA, NA, NA, NA, NA,

NA, NA, NA, NA, NA, NA, NA, NA, NA, NA, NA, NA, NA, NA, NA, NA, NA, NA, NA, NA,

NA, NA, NA, NA, NA, NA, NA, NA, NA, NA, NA, NA, NA, NA, NA, NA, NA, NA, NA, NA,

NA, NA, NA, NA, NA, NA, NA, NA, NA, NA, NA, NA, NA, NA, NA, NA, NA, NA, NA, NA,

NA, NA, NA, NA, NA, NA, NA, NA, NA, NA, NA, NA, NA, NA, NA, NA, NA, NA, NA, NA,

NA, NA, NA, NA, NA, NA, NA, NA, NA, NA, NA, NA, NA, NA, NA, NA, NA, NA, NA, NA,

NA, NA, NA, NA, NA, NA, NA, NA, NA, NA, NA, NA, NA, NA, NA, NA, NA, NA, NA, NA,

NA, NA, NA, NA, NA, NA, NA, NA, NA, NA, NA, NA, NA, NA, NA, NA, NA, NA, NA, NA,

NA, NA, NA, NA, NA, NA, NA, NA, NA, NA, NA, NA, NA, NA, NA, NA, NA, NA, NA, NA,

NA, NA, NA, NA, NA, NA, NA, NA, NA, NA, NA, NA, NA, NA, NA, NA, NA, NA, NA, NA,

NA, NA, NA, NA, NA, NA, NA, NA, NA, NA, NA, NA, NA, NA, NA, NA, NA, NA, NA, NA,

NA, NA, NA, NA, NA, NA, NA, NA, NA, NA, NA, NA, NA, NA, NA, NA, NA, NA, NA, NA,

NA, NA, NA, NA, NA, NA, NA, NA, NA, NA, NA, NA, NA, NA, NA, NA, NA, NA, NA, NA,

NA, NA, NA, NA, NA, NA, NA, NA, NA, NA, NA, NA, NA, NA, NA, NA, NA, NA, NA, NA,

NA, NA, NA, NA, NA, NA, NA, NA, NA, NA, NA, NA, NA, NA, NA, NA, NA, NA, NA, NA,

NA, NA, NA, NA, NA, NA, NA, NA, NA, NA, NA, NA, NA, NA, NA, NA, NA, NA, NA, NA,

NA, NA, NA, NA, NA, NA, NA, NA, NA, NA, NA, NA, NA, NA, NA, NA, NA, NA, NA, NA,

NA, NA, NA, NA, NA, NA, NA, NA, NA, NA, NA, NA, NA, NA, NA, NA, NA, NA, NA, NA,

NA, NA, NA, NA, NA, NA, NA, NA, NA, NA, NA, NA, NA, NA, NA, NA, NA, NA, NA, NA,

NA, NA, NA, NA, NA, NA, NA, NA, NA, NA, NA, NA, NA, NA, NA, NA, NA, NA, NA, NA,

NA, NA, NA, NA, NA, NA, NA, NA, NA, NA, NA, NA, NA, NA, NA, NA, NA, NA, NA, NA,

NA, NA, NA, NA, NA, NA, NA, NA, NA, NA, NA, NA, NA, NA, NA, NA, NA, NA, NA, NA,

NA, NA, NA, NA, NA, NA, NA, NA, NA, NA, NA, NA, NA, NA, NA, NA, NA, NA, NA, NA,

NA, NA, NA, NA, NA, NA, NA, NA, NA, NA, NA, NA, NA, NA, NA, NA, NA, NA, NA, NA,

NA, NA, NA, NA, NA, NA, NA, NA, NA, NA, NA, NA, NA, NA, NA, NA, NA, NA, NA, NA,

NA, NA, NA, NA, NA, NA, NA, NA, NA, NA, NA, NA, NA, NA, NA, NA, NA, NA, NA, NA,

#H BNA90+

1, 1, 1, 1, 1, 1, 1, 1, 1, 1, 1, 1, 1, 1, 1, 1, 1, 1, 1, 1,

1, 1, 1, 1, 1, 1, 1, 1, 1, 1, 1, 1, 1, 1, 1, 1, 1, 1, 1, 1,

1, 1, 1, 1, 1, 1, 1, 1, 1, 1, 1, 1, 1, 1, 1, 1, 1, 1, 1, 1,

1, 1, 1, 1, 1, 1, 1, 1, 1, 1, 1, 1, 1, 1, NA, NA, NA, NA, NA, NA,

NA, NA, NA, NA, NA, NA, NA, NA, NA, NA, NA, NA, NA, NA, NA, NA, NA, NA, NA, NA,

NA, NA, NA, NA, NA, NA, NA, NA, NA, NA, NA, NA, NA, NA, NA, NA, NA, NA, NA, NA,

NA, NA, NA, NA, NA, NA, NA, NA, NA, NA, NA, NA, NA, NA, NA, NA, NA, NA, NA, NA,

NA, NA, NA, NA, NA, NA, NA, NA, NA, NA, NA, NA, NA, NA, NA, NA, NA, NA, NA, NA,

NA, NA, NA, NA, NA, NA, NA, NA, NA, NA, NA, NA, NA, NA, NA, NA, NA, NA, NA, NA,

NA, NA, NA, NA, NA, NA, NA, NA, NA, NA, NA, NA, NA, NA, NA, NA, NA, NA, NA, NA,

NA, NA, NA, NA, NA, NA, NA, NA, NA, NA, NA, NA, NA, NA, NA, NA, NA, NA, NA, NA,

NA, NA, NA, NA, NA, NA, NA, NA, NA, NA, NA, NA, NA, NA, NA, NA, NA, NA, NA, NA,

NA, NA, NA, NA, NA, NA, NA, NA, NA, NA, NA, NA, NA, NA, NA, NA, NA, NA, NA, NA,

NA, NA, NA, NA, NA, NA, NA, NA, NA, NA, NA, NA, NA, NA, NA, NA, NA, NA, NA, NA,

NA, NA, NA, NA, NA, NA, NA, NA, NA, NA, NA, NA, NA, NA, NA, NA, NA, NA, NA, NA,

NA, NA, NA, NA, NA, NA, NA, NA, NA, NA, NA, NA, NA, NA, NA, NA, NA, NA, NA, NA,

NA, NA, NA, NA, NA, NA, NA, NA, NA, NA, NA, NA, NA, NA, NA, NA, NA, NA, NA, NA,

NA, NA, NA, NA, NA, NA, NA, NA, NA, NA, NA, NA, NA, NA, NA, NA, NA, NA, NA, NA,

NA, NA, NA, NA, NA, NA, NA, NA, NA, NA, NA, NA, NA, NA, NA, NA, NA, NA, NA, NA,

NA, NA, NA, NA, NA, NA, NA, NA, NA, NA, NA, NA, NA, NA, NA, NA, NA, NA, NA, NA,

NA, NA, NA, NA, NA, NA, NA, NA, NA, NA, NA, NA, NA, NA, NA, NA, NA, NA, NA, NA,

NA, NA, NA, NA, NA, NA, NA, NA, NA, NA, NA, NA, NA, NA, NA, NA, NA, NA, NA, NA,

NA, NA, NA, NA, NA, NA, NA, NA, NA, NA, NA, NA, NA, NA, NA, NA, NA, NA, NA, NA,

NA, NA, NA, NA, NA, NA, NA, NA, NA, NA, NA, NA, NA, NA, NA, NA, NA, NA, NA, NA,

NA, NA, NA, NA, NA, NA, NA, NA, NA, NA, NA, NA, NA, NA, NA, NA, NA, NA, NA, NA,

NA, NA, NA, NA, NA, NA, NA, NA, NA, NA, NA, NA, NA, NA, NA, NA, NA, NA, NA, NA,

NA, NA, NA, NA, NA, NA, NA, NA, NA, NA, NA, NA, NA, NA, NA, NA, NA, NA, NA, NA,

NA, NA, NA, NA, NA, NA, NA, NA, NA, NA, NA, NA, NA, NA, NA, NA, NA, NA, NA, NA,

NA, NA, NA, NA, NA, NA, NA, NA, NA, NA, NA, NA, NA, NA, NA, NA, NA, NA, NA, NA,

NA, NA, NA, NA, NA, NA, NA, NA, NA, NA, NA, NA, NA, NA, NA, NA, NA, NA, NA, NA,

NA, NA, NA, NA, NA, NA, NA, NA, NA, NA, NA, NA, NA, NA, NA, NA, NA, NA, NA, NA,

NA, NA, NA, NA, NA, NA, NA, NA, NA, NA, NA, NA, NA, NA, NA, NA, NA, NA, NA, NA,

NA, NA, NA, NA, NA, NA, NA, NA, NA, NA, NA, NA, NA, NA, NA, NA, NA, NA, NA, NA,

NA, NA, NA, NA, NA, NA, NA, NA, NA, NA, NA, NA, NA, NA, NA, NA, NA, NA, NA, NA,

NA, NA, NA, NA, NA, NA, NA, NA, NA, NA, NA, NA, NA, NA, NA, NA, NA, NA, NA, NA,

NA, NA, NA, NA, NA, NA, NA, NA, NA, NA, NA, NA, NA, NA, NA, NA, NA, NA, NA, NA,

NA, NA, NA, NA, NA, NA, NA, NA, NA, NA, NA, NA, NA, NA, NA, NA, NA, NA, NA, NA,

NA, NA, NA, NA, NA, NA, NA, NA, NA, NA, NA, NA, NA, NA, NA, NA, NA, NA, NA, NA,

NA, NA, NA, NA, NA, NA, NA, NA, NA, NA, NA, NA, NA, NA, NA, NA, NA, NA, NA, NA,

NA, NA, NA, NA, NA, NA, NA, NA, NA, NA, NA, NA, NA, NA, NA, NA, NA, NA, NA, NA,

NA, NA, NA, NA, NA, NA, NA, NA, NA, NA, NA, NA, NA, NA, NA, NA, NA, NA, NA, NA,

NA, NA, NA, NA, NA, NA, NA, NA, NA, NA, NA, NA, NA, NA, NA, NA, NA, NA, NA, NA,

NA, NA, NA, NA, NA, NA, NA, NA, NA, NA, NA, NA, NA, NA, NA, NA, NA, NA, NA, NA,

NA, NA, NA, NA, NA, NA, NA, NA, NA, NA, NA, NA, NA, NA, NA, NA, NA, NA, NA, NA,

NA, NA, NA, NA, NA, NA, NA, NA, NA, NA, NA, NA, NA, NA, NA, NA, NA, NA, NA, NA,

NA, NA, NA, NA, NA, NA, NA, NA, NA, NA, NA, NA, NA, NA, NA, NA, NA, NA, NA, NA,

NA, NA, NA, NA, NA, NA, NA, NA, NA, NA, NA, NA, NA, NA, NA, NA, NA, NA, NA, NA,

NA, NA, NA, NA, NA, NA, NA, NA, NA, NA, NA, NA, NA, NA, NA, NA, NA, NA, NA, NA,

NA, NA, NA, NA, NA, NA, NA, NA, NA, NA, NA, NA, NA, NA, NA, NA, NA, NA, NA, NA,

NA, NA, NA, NA, NA, NA, NA, NA, NA, NA, NA, NA, NA, NA, NA, NA, NA, NA, NA, NA,

NA, NA, NA, NA, NA, NA, NA, NA, NA, NA, NA, NA, NA, NA, NA, NA, NA, NA, NA, NA,

NA, NA, NA, NA, NA, NA, NA, NA, NA, NA, NA, NA, NA, NA, NA, NA, NA, NA, NA, NA,

NA, NA, NA, NA, NA, NA, NA, NA, NA, NA, NA, NA, NA, NA, NA, NA, NA, NA, NA, NA,

NA, NA, NA, NA, NA, NA, NA, NA, NA, NA, NA, NA, NA, NA, NA, NA, NA, NA, NA, NA,

NA, NA, NA, NA, NA, NA, NA, NA, NA, NA, NA, NA, NA, NA, NA, NA, NA, NA, NA, NA,

NA, NA, NA, NA, NA, NA, NA, NA, NA, NA, NA, NA, NA, NA, NA, NA, NA, NA, NA, NA,

NA, NA, NA, NA, NA, NA, NA, NA, NA, NA, NA, NA, NA, NA, NA, NA, NA, NA, NA, NA,

NA, NA, NA, NA, NA, NA, NA, NA, NA, NA, NA, NA, NA, NA, NA, NA, NA, NA, NA, NA,

NA, NA, NA, NA, NA, NA, NA, NA, NA, NA, NA, NA, NA, NA, NA, NA, NA, NA, NA, NA,

NA, NA, NA, NA, NA, NA, NA, NA, NA, NA, NA, NA, NA, NA, NA, NA, NA, NA, NA, NA,

NA, NA, NA, NA, NA, NA, NA, NA, NA, NA, NA, NA, NA, NA, NA, NA, NA, NA, NA, NA,

NA, NA, NA, NA, NA, NA, NA, NA, NA, NA, NA, NA, NA, NA, NA, NA, NA, NA, NA, NA,

NA, NA, NA, NA, NA, NA, NA, NA, NA, NA, NA, NA, NA, NA, NA, NA, NA, NA, NA, NA,

NA, NA, NA, NA, NA, NA, NA, NA, NA, NA, NA, NA, NA, NA, NA, NA, NA, NA, NA, NA,

NA, NA, NA, NA, NA, NA, NA, NA, NA, NA, NA, NA, NA, NA, NA, NA, NA, NA, NA, NA,

NA, NA, NA, NA, NA, NA, NA, NA, NA, NA, NA, NA, NA, NA, NA, NA, NA, NA, NA, NA,

NA, NA, NA, NA, NA, NA, NA, NA, NA, NA, NA, NA, NA, NA, NA, NA, NA, NA, NA, NA,

NA, NA, NA, NA, NA, NA, NA, NA, NA, NA, NA, NA, NA, NA, NA, NA, NA, NA, NA, NA,

NA, NA, NA, NA, NA, NA, NA, NA, NA, NA, NA, NA, NA, NA, NA, NA, NA, NA, NA, NA,

NA, NA, NA, NA, NA, NA, NA, NA, NA, NA, NA, NA, NA, NA, NA, NA, NA, NA, NA, NA,

#F HIC -84

1, 1, 1, 1, 1, 1, 1, 1, 1, 1, 1, 1, 1, 1, 1, 1, 1, 1, 1, 1,

1, 1, 1, 1, 1, 1, 1, 1, 1, 1, 1, 1, 1, 1, 1, 1, 1, 1, 1, 1,

1, 1, 1, 1, 1, 1, 1, 1, 1, 1, 1, 1, 1, 1, 1, 1, 1, 1, 1, 1,

1, 1, 1, 1, 1, 1, 1, 1, 1, 1, 1, 1, 1, 1, 1, 1, 1, 1, 1, 1,

1, 1, 1, 1, 1, 1, 1, 1, 1, 1, 1, 1, 1, 1, 1, 1, 1, 1, 1, 1,

1, 1, 1, 1, 1, 1, 1, 1, 1, 1, 1, 1, 1, 1, 1, 1, 1, 1, 1, 1,

1, 1, 1, 1, 1, 1, 1, 1, 1, 1, 1, 1, 1, 1, 1, 1, 1, 1, 1, 1,

1, 1, 1, 1, 1, 1, 1, 1, 1, 1, 1, 1, 1, 1, 1, 1, 1, 1, 1, 1,

1, 1, 1, 1, 1, 1, 1, 1, 1, 1, 1, 1, 1, 1, 1, 1, 1, 1, 1, 1,

1, 1, 1, 1, 1, 1, 1, 1, 1, 1, 1, 1, 1, 1, 1, 1, 1, 1, 1, 1,

1, 1, 1, 1, 1, 1, 1, 1, 1, 1, 1, 1, 1, 1, 1, 1, 1, 1, 1, 1,

1, 1, 1, 1, 1, 1, 1, 1, 1, 1, 1, 1, 1, 1, 1, 1, 1, 1, 1, 1,

1, 1, 1, 1, 1, 1, 1, 1, 1, 1, 1, 1, 1, 1, 1, 1, 1, 1, 1, 1,

1, 1, 1, 1, 1, 1, 1, 1, 1, 1, 1, 1, 1, 1, 1, 1, 1, 1, 1, 1,

1, 1, 1, 1, 1, 1, 1, 1, 1, 1, 1, 1, 1, 1, 1, 1, 1, 1, 1, 1,

1, 1, 1, 1, 1, 1, 1, 1, 1, 1, 1, 1, 1, 1, 1, 1, 1, 1, 1, 1,

1, 1, 1, 1, 1, 1, 1, 1, 1, 1, 1, 1, 1, 1, 1, 1, 1, 1, 1, 1,

1, 1, 1, 1, 1, 1, 1, 1, 1, 1, 1, 1, 1, 1, 1, 1, 1, 1, 1, 1,

1, 1, 1, 1, 1, 1, 1, 1, 1, 1, 1, 1, 1, 1, 1, 1, 1, 1, 1, 1,

1, 1, 1, 1, 1, 1, 1, 1, 1, 1, 1, 1, 1, 1, 1, 1, 1, 1, 1, 1,

1, 1, 1, 1, 1, 1, 1, 1, 1, 1, 1, 1, 1, 1, 1, 1, 1, 1, 1, 1,

1, 1, 1, 1, 1, 1, 1, 1, 1, 1, 1, 1, 1, 1, 1, 1, 1, 1, 1, 1,

1, 1, 1, 1, 1, 1, 1, 1, 1, 1, 1, 1, 1, 1, 1, 1, 1, 1, 1, 1,

1, 1, 1, 1, 1, 1, 1, 1, 1, 1, 1, 1, 1, 1, 1, 1, 1, 1, 1, 1,

1, 1, 1, 1, 1, 1, 1, 1, 1, 1, 1, 1, 1, 1, 1, 1, 1, 1, 1, 1,

1, 1, 1, 1, 1, 1, 1, 1, 1, 1, 1, 1, 1, 1, 1, 1, 1, 1, 1, 1,

1, 1, 1, 1, 1, 1, 1, 1, 1, 1, 1, 1, 1, 1, 1, 1, 1, 1, 1, 1,

1, 1, 1, 1, 1, 1, 1, 1, 1, 1, 1, 1, 1, 1, 1, 1, 1, 1, 1, 1,

1, 1, 1, 1, 1, 1, 1, 1, 1, 1, 1, 1, 1, 1, 1, 1, 1, 1, 1, 1,

1, 1, 1, 1, 1, 1, 1, 1, 1, 1, 1, 1, 1, 1, 1, 1, 1, 1, 1, 1,

1, 1, 1, 1, 1, 1, 1, 1, 1, 1, 1, 1, 1, 1, 1, 1, 1, 1, 1, 1,

1, 1, 1, 1, 1, 1, 1, 1, 1, 1, 1, 1, 1, 1, 1, 1, 1, 1, 1, 1,

1, 1, 1, 1, 1, 1, 1, 1, 1, 1, 1, 1, 1, 1, 1, 1, 1, 1, 1, 1,

1, 1, 1, 1, 1, 1, 1, 1, 1, 1, 1, 1, 1, 1, 1, 1, 1, 1, 1, 1,

1, 1, 1, 1, 1, 1, 1, 1, 1, 1, 1, 1, 1, 1, 1, 1, 1, 1, 1, 1,

1, 1, 1, 1, 1, 1, 1, 1, 1, 1, 1, 1, 1, 1, 1, 1, 1, 1, 1, 1,

1, 1, 1, 1, 1, 1, 1, 1, 1, 1, 1, 1, 1, 1, 1, 1, 1, 1, 1, 1,

1, 1, 1, 1, 1, 1, 1, 1, 1, 1, 1, 1, 1, 1, 1, 1, 1, 1, 1, 1,

1, 1, 1, 1, 1, 1, 1, 1, 1, 1, 1, 1, 1, 1, 1, 1, 1, 1, 1, 1,

1, 1, 1, 1, 1, 1, 1, 1, 1, 1, 1, 1, 1, 1, 1, 1, 1, 1, 1, 1,

1, 1, 1, 1, 1, 1, 1, 1, 1, 1, 1, 1, 1, 1, 1, 1, 1, 1, 1, 1,

1, 1, 1, 1, 1, 1, 1, 1, 1, 1, 1, 1, 1, 1, 1, 1, 1, 1, 1, 1,

1, 1, 1, 1, 1, 1, 1, 1, 1, 1, 1, 1, 1, 1, 1, 1, 1, 1, 1, 1,

1, 1, 1, 1, 1, 1, 1, 1, 1, 1, 1, 1, 1, 1, 1, 1, 1, 1, 1, 1,

1, 1, 1, 1, 1, 1, 1, 1, 1, 1, 1, 1, 1, 1, 1, 1, 1, 1, 1, 1,

1, 1, 1, 1, 1, 1, 1, 1, 1, 1, 1, 1, 1, 1, 1, 1, 1, 1, 1, 1,

1, 1, 1, 1, 1, 1, 1, 1, 1, 1, 1, 1, 1, 1, 1, 1, 1, 1, 1, 1,

1, 1, 1, 1, 1, 1, 1, 1, 1, 1, 1, 1, 1, 1, 1, 1, 1, 1, 1, 1,

1, 1, 1, 1, 1, 1, 1, 1, 1, 1, 1, 1, 1, 1, 1, 1, 1, 1, 1, 1,

1, 1, 1, 1, 1, 1, 1, 1, 1, 1, 1, 1, 1, 1, 1, 1, 1, 1, 1, 1,

1, 1, 1, 1, 1, 1, 1, 1, 1, 1, 1, 1, 1, 1, 1, 1, 1, 1, 1, 1,

1, 1, 1, 1, 1, 1, 1, 1, 1, 1, 1, 1, 1, 1, 1, 1, 1, 1, 1, 1,

1, 1, 1, 1, 1, 1, 1, 1, 1, 1, 1, 1, 1, 1, 1, 1, 1, 1, 1, 1,

1, 1, 1, 1, 1, 1, 1, 1, 1, 1, 1, 1, 1, 1, 1, 1, 1, 1, 1, 1,

1, 1, 1, 1, 1, 1, 1, 1, 1, 1, 1, 1, 1, 1, 1, 1, 1, 1, 1, 1,

1, 1, 1, 1, 1, 1, 1, 1, 1, 1, 1, 1, 1, 1, 1, 1, 1, 1, 1, 1,

1, 1, 1, 1, 1, 1, 1, 1, 1, 1, 1, 1, 1, 1, 1, 1, 1, 1, 1, 1,

1, 1, 1, 1, 1, 1, 1, 1, 1, 1, 1, 1, 1, 1, 1, 1, 1, 1, 1, 1,

1, 1, 1, 1, 1, 1, 1, 1, 1, 1, 1, 1, 1, 1, 1, 1, 1, 1, 1, 1,

1, 1, 1, 1, 1, 1, 1, 1, 1, 1, 1, 1, 1, 1, 1, 1, 1, 1, 1, 1,

1, 1, 1, 1, 1, 1, 1, 1, 1, 1, 1, 1, 1, 1, 1, 1, 1, 1, 1, 1,

1, 1, 1, 1, 1, 1, 1, 1, 1, 1, 1, 1, 1, 1, 1, 1, 1, 1, 1, 1,

1, 1, 1, 1, 1, 1, 1, 1, 1, 1, 1, 1, 1, 1, 1, 1, 1, 1, 1, 1,

1, 1, 1, 1, 1, 1, 1, 1, 1, 1, 1, 1, 1, 1, 1, 1, 1, 1, 1, 1,

1, 1, 1, 1, 1, 1, 1, 1, 1, 1, 1, 1, 1, 1, 1, 1, 1, 1, 1, 1,

1, 1, 1, 1, 1, 1, 1, 1, 1, 1, 1, 1, 1, 1, 1, 1, 1, 1, 1, 1,

1, 1, 1, 1, 1, 1, 1, 1, 1, 1, 1, 1, 1, 1, 1, 1, 1, 1, 1, 1,

1, 1, 1, 1, 1, 1, 1, 1, 1, 1, 1, 1, 1, 1, 1, 1, 1, 1, 1, 1,

1, 1, 1, 1, 1, 1, 1, 1, 1, 1, 1, 1, 1, 1, 1, 1, 1, 1, 1, 1,

1, 1, 1, 1, 1, NA, NA, NA, NA, NA, NA, NA, NA, NA, NA, NA, NA, NA, NA, NA,

# F BNA84-

1, 1, 1, 1, 1, 1, 1, 1, 1, 1, 1, 1, 1, 1, 1, 1, 1, 1, 1, 1,

1, 1, 1, 1, 1, 1, 1, 1, 1, 1, 1, 1, 1, 1, 1, 1, 1, 1, 1, 1,

1, 1, 1, 1, 1, 1, 1, 1, 1, 1, 1, 1, 1, 1, 1, 1, 1, 1, 1, 1,

1, 1, 1, 1, 1, 1, 1, 1, 1, 1, 1, 1, 1, 1, 1, 1, 1, 1, 1, 1,

1, 1, 1, 1, 1, 1, 1, 1, 1, 1, 1, 1, 1, 1, 1, 1, 1, 1, 1, 1,

1, 1, 1, 1, 1, 1, 1, 1, 1, 1, 1, 1, 1, 1, 1, 1, 1, 1, 1, 1,

1, 1, 1, 1, 1, 1, 1, 1, 1, 1, 1, 1, 1, 1, 1, 1, 1, 1, 1, 1,

1, 1, 1, 1, 1, 1, 1, 1, 1, 1, 1, 1, 1, 1, 1, 1, 1, 1, 1, 1,

1, 1, 1, 1, 1, 1, 1, 1, 1, 1, 1, 1, 1, 1, 1, 1, 1, 1, 1, 1,

1, 1, 1, 1, 1, 1, 1, 1, 1, 1, 1, 1, 1, 1, 1, 1, 1, 1, 1, 1,

1, 1, 1, 1, 1, 1, 1, 1, 1, 1, 1, 1, 1, 1, 1, 1, 1, 1, 1, 1,

1, 1, 1, 1, 1, 1, 1, 1, 1, 1, 1, 1, 1, 1, 1, 1, 1, 1, 1, 1,

1, 1, 1, 1, 1, 1, 1, 1, 1, 1, 1, 1, 1, 1, 1, 1, 1, 1, 1, 1,

1, 1, 1, 1, 1, 1, 1, 1, 1, 1, 1, 1, 1, 1, 1, 1, 1, 1, 1, 1,

1, 1, 1, 1, 1, 1, 1, 1, 1, 1, 1, 1, 1, 1, 1, 1, 1, 1, 1, 1,

1, 1, 1, 1, 1, 1, 1, 1, 1, 1, 1, 1, 1, 1, 1, 1, 1, 1, 1, 1,

1, 1, 1, 1, 1, 1, 1, 1, 1, 1, 1, 1, 1, 1, 1, 1, 1, 1, 1, 1,

1, 1, 1, 1, 1, 1, 1, 1, 1, 1, 1, 1, 1, 1, 1, 1, 1, 1, 1, 1,

1, 1, 1, 1, 1, 1, 1, 1, 1, 1, 1, 1, 1, 1, 1, 1, 1, 1, 1, 1,

1, 1, 1, 1, 1, 1, 1, 1, 1, 1, 1, 1, 1, 1, 1, 1, 1, 1, 1, 1,

1, 1, 1, 1, 1, 1, 1, 1, 1, 1, 1, 1, 1, 1, 1, 1, 1, 1, 1, 1,

1, 1, 1, 1, 1, 1, 1, 1, 1, 1, 1, 1, 1, 1, 1, 1, 1, 1, 1, 1,

1, 1, 1, 1, 1, 1, 1, 1, 1, 1, 1, 1, 1, 1, 1, 1, 1, 1, 1, 1,

1, 1, 1, 1, 1, 1, 1, 1, 1, 1, 1, 1, 1, 1, 1, 1, 1, 1, 1, 1,

1, 1, 1, 1, 1, 1, 1, 1, 1, 1, 1, 1, 1, 1, 1, 1, 1, 1, 1, 1,

1, 1, 1, 1, 1, 1, 1, 1, 1, 1, 1, 1, 1, 1, 1, 1, 1, 1, 1, 1,

1, 1, 1, 1, 1, 1, 1, 1, 1, 1, 1, 1, 1, 1, 1, 1, 1, 1, 1, 1,

1, 1, 1, 1, 1, 1, 1, 1, 1, 1, 1, 1, 1, 1, 1, 1, 1, 1, 1, 1,

1, 1, 1, 1, 1, 1, 1, 1, 1, 1, 1, 1, 1, 1, 1, 1, 1, 1, 1, 1,

1, 1, 1, 1, 1, 1, 1, 1, 1, 1, 1, 1, 1, 1, 1, 1, 1, 1, 1, 1,

1, 1, 1, 1, 1, 1, 1, 1, 1, 1, 1, 1, 1, 1, 1, 1, 1, 1, 1, 1,

1, 1, 1, 1, 1, 1, 1, 1, 1, 1, 1, 1, 1, 1, 1, 1, 1, 1, 1, 1,

1, 1, 1, 1, 1, 1, 1, 1, 1, 1, 1, 1, 1, 1, 1, 1, 1, 1, 1, 1,

1, 1, 1, 1, 1, 1, 1, 1, 1, 1, 1, 1, 1, 1, 1, 1, 1, 1, 1, 1,

1, 1, 1, 1, 1, 1, 1, 1, 1, 1, 1, 1, 1, 1, 1, 1, 1, 1, 1, 1,

1, 1, 1, 1, 1, 1, 1, 1, 1, 1, 1, 1, 1, 1, 1, 1, 1, 1, 1, 1,

1, 1, 1, 1, 1, 1, 1, 1, 1, 1, 1, 1, 1, 1, 1, 1, 1, 1, 1, 1,

1, 1, 1, 1, 1, 1, 1, 1, 1, 1, 1, 1, 1, 1, 1, 1, 1, 1, 1, 1,

1, 1, 1, 1, 1, 1, 1, 1, 1, 1, 1, 1, 1, 1, 1, 1, 1, 1, 1, 1,

1, 1, 1, 1, 1, 1, 1, 1, 1, 1, 1, 1, 1, 1, 1, 1, 1, 1, 1, 1,

1, 1, 1, 1, 1, 1, 1, 1, 1, 1, 1, 1, 1, 1, 1, 1, 1, 1, 1, 1,

1, 1, 1, 1, 1, 1, 1, 1, 1, 1, 1, 1, 1, 1, 1, 1, 1, 1, 1, 1,

1, 1, 1, 1, 1, 1, 1, 1, 1, 1, 1, 1, 1, 1, 1, 1, 1, 1, 1, 1,

1, 1, 1, 1, 1, 1, 1, 1, 1, 1, 1, 1, 1, 1, 1, 1, 1, 1, 1, 1,

1, 1, 1, 1, 1, 1, 1, 1, 1, 1, 1, 1, 1, 1, 1, 1, 1, 1, 1, 1,

1, 1, 1, 1, 1, 1, 1, 1, 1, 1, 1, 1, 1, 1, 1, 1, 1, 1, 1, 1,

1, 1, 1, 1, 1, 1, 1, 1, 1, 1, 1, 1, 1, 1, 1, 1, 1, 1, 1, 1,

1, 1, 1, 1, 1, 1, 1, 1, 1, 1, 1, 1, 1, 1, 1, 1, 1, 1, 1, 1,

1, 1, 1, 1, 1, 1, 1, 1, 1, 1, 1, 1, 1, 1, 1, 1, 1, 1, 1, 1,

1, 1, 1, 1, 1, 1, 1, 1, 1, 1, 1, 1, 1, 1, 1, 1, 1, 1, 1, 1,

1, 1, 1, 1, 1, 1, 1, 1, 1, 1, 1, 1, 1, NA, NA, NA, NA, NA, NA, NA,

NA, NA, NA, NA, NA, NA, NA, NA, NA, NA, NA, NA, NA, NA, NA, NA, NA, NA, NA, NA,

NA, NA, NA, NA, NA, NA, NA, NA, NA, NA, NA, NA, NA, NA, NA, NA, NA, NA, NA, NA,

NA, NA, NA, NA, NA, NA, NA, NA, NA, NA, NA, NA, NA, NA, NA, NA, NA, NA, NA, NA,

NA, NA, NA, NA, NA, NA, NA, NA, NA, NA, NA, NA, NA, NA, NA, NA, NA, NA, NA, NA,

NA, NA, NA, NA, NA, NA, NA, NA, NA, NA, NA, NA, NA, NA, NA, NA, NA, NA, NA, NA,

NA, NA, NA, NA, NA, NA, NA, NA, NA, NA, NA, NA, NA, NA, NA, NA, NA, NA, NA, NA,

NA, NA, NA, NA, NA, NA, NA, NA, NA, NA, NA, NA, NA, NA, NA, NA, NA, NA, NA, NA,

NA, NA, NA, NA, NA, NA, NA, NA, NA, NA, NA, NA, NA, NA, NA, NA, NA, NA, NA, NA,

NA, NA, NA, NA, NA, NA, NA, NA, NA, NA, NA, NA, NA, NA, NA, NA, NA, NA, NA, NA,

NA, NA, NA, NA, NA, NA, NA, NA, NA, NA, NA, NA, NA, NA, NA, NA, NA, NA, NA, NA,

NA, NA, NA, NA, NA, NA, NA, NA, NA, NA, NA, NA, NA, NA, NA, NA, NA, NA, NA, NA,

NA, NA, NA, NA, NA, NA, NA, NA, NA, NA, NA, NA, NA, NA, NA, NA, NA, NA, NA, NA,

NA, NA, NA, NA, NA, NA, NA, NA, NA, NA, NA, NA, NA, NA, NA, NA, NA, NA, NA, NA,

NA, NA, NA, NA, NA, NA, NA, NA, NA, NA, NA, NA, NA, NA, NA, NA, NA, NA, NA, NA,

NA, NA, NA, NA, NA, NA, NA, NA, NA, NA, NA, NA, NA, NA, NA, NA, NA, NA, NA, NA,

NA, NA, NA, NA, NA, NA, NA, NA, NA, NA, NA, NA, NA, NA, NA, NA, NA, NA, NA, NA,

NA, NA, NA, NA, NA, NA, NA, NA, NA, NA, NA, NA, NA, NA, NA, NA, NA, NA, NA, NA,

NA, NA, NA, NA, NA, NA, NA, NA, NA, NA, NA, NA, NA, NA, NA, NA, NA, NA, NA, NA,

NA, NA, NA, NA, NA, NA, NA, NA, NA, NA, NA, NA, NA, NA, NA, NA, NA, NA, NA, NA,

# F TRT85-89

1, 1, 1, 1, 1, 1, 1, 1, 1, 1, 1, 1, 1, 1, 1, 1, 1, 1, 1, 1,

1, 1, 1, 1, 1, 1, 1, 1, 1, 1, 1, 1, 1, 1, 1, 1, 1, 1, 1, 1,

1, 1, 1, 1, 1, 1, 1, 1, 1, 1, 1, 1, 1, 1, 1, 1, 1, 1, 1, 1,

1, 1, 1, 1, 1, 1, 1, 1, 1, 1, 1, 1, 1, 1, 1, 1, 1, 1, 1, 1,

1, 1, 1, 1, 1, 1, 1, 1, 1, 1, 1, 1, 1, 1, 1, 1, 1, 1, 1, 1,

1, 1, 1, 1, 1, 1, 1, 1, 1, 1, 1, 1, 1, 1, 1, 1, 1, 1, 1, 1,

1, 1, 1, 1, 1, 1, 1, 1, 1, 1, 1, 1, 1, 1, 1, 1, 1, 1, 1, 1,

1, 1, 1, 1, 1, 1, 1, 1, 1, 1, 1, 1, 1, 1, 1, 1, 1, 1, 1, 1,

1, 1, 1, 1, 1, 1, 1, 1, 1, 1, 1, 1, 1, 1, 1, 1, 1, 1, 1, 1,

1, 1, 1, 1, 1, 1, 1, 1, 1, 1, 1, 1, 1, 1, 1, 1, 1, 1, 1, 1,

1, 1, 1, 1, 1, 1, 1, 1, 1, 1, 1, 1, 1, 1, 1, 1, 1, 1, 1, 1,

1, 1, 1, 1, 1, 1, 1, 1, 1, 1, 1, 1, 1, 1, 1, 1, 1, 1, 1, 1,

1, 1, 1, 1, 1, 1, 1, 1, 1, 1, 1, 1, 1, 1, 1, 1, 1, 1, 1, 1,

1, 1, 1, 1, 1, 1, 1, 1, 1, 1, 1, 1, 1, 1, 1, 1, 1, 1, 1, 1,

1, 1, 1, 1, 1, 1, 1, 1, 1, 1, 1, 1, 1, 1, 1, 1, 1, 1, 1, 1,

1, 1, 1, 1, 1, 1, 1, 1, 1, 1, 1, 1, 1, 1, 1, 1, 1, 1, 1, 1,

1, 1, 1, 1, 1, 1, 1, 1, 1, 1, 1, 1, 1, 1, 1, 1, 1, 1, 1, 1,

1, 1, 1, 1, 1, 1, 1, 1, 1, 1, 1, 1, 1, 1, 1, 1, 1, 1, 1, 1,

1, 1, 1, 1, 1, 1, 1, 1, 1, 1, 1, 1, 1, 1, 1, 1, 1, 1, 1, 1,

1, 1, 1, 1, 1, 1, 1, 1, 1, 1, 1, 1, 1, 1, 1, 1, 1, 1, 1, 1,

1, 1, 1, 1, 1, 1, 1, 1, 1, 1, 1, 1, 1, 1, 1, 1, 1, 1, 1, 1,

1, 1, 1, 1, 1, 1, 1, 1, 1, 1, 1, 1, 1, 1, 1, 1, 1, 1, 1, 1,

1, 1, 1, 1, 1, 1, 1, 1, 1, 1, 1, 1, 1, 1, 1, 1, 1, 1, 1, 1,

1, 1, 1, 1, 1, 1, 1, 1, 1, 1, 1, 1, 1, 1, 1, 1, 1, 1, 1, 1,

1, 1, 1, 1, 1, 1, 1, 1, 1, 1, 1, 1, 1, 1, 1, 1, 1, 1, 1, 1,

1, 1, 1, 1, 1, 1, 1, 1, 1, 1, 1, 1, 1, 1, 1, 1, 1, 1, 1, 1,

1, 1, 1, 1, 1, 1, 1, 1, 1, 1, 1, 1, 1, 1, 1, 1, 1, 1, 1, 1,

1, 1, 1, 1, 1, 1, 1, 1, 1, 1, 1, 1, 1, 1, 1, 1, 1, 1, 1, 1,

1, 1, 1, 1, 1, 1, 1, 1, 1, 1, 1, 1, 1, 1, 1, 1, 1, 1, 1, 1,

1, 1, 1, 1, 1, 1, 1, 1, 1, 1, 1, 1, 1, 1, 1, 1, 1, 1, 1, 1,

1, 1, 1, 1, 1, 1, 1, 1, 1, 1, 1, 1, 1, 1, 1, 1, 1, 1, 1, 1,

1, 1, 1, 1, 1, 1, 1, 1, 1, 1, 1, 1, 1, 1, 1, 1, 1, 1, 1, 1,

1, 1, 1, 1, 1, 1, 1, 1, 1, 1, 1, 1, 1, 1, 1, 1, 1, 1, 1, 1,

1, 1, 1, 1, 1, 1, 1, 1, 1, 1, 1, 1, 1, 1, 1, 1, 1, 1, 1, 1,

1, 1, 1, 1, 1, 1, 1, 1, 1, 1, 1, 1, 1, 1, 1, 1, 1, 1, 1, 1,

1, 1, 1, 1, 1, 1, 1, 1, 1, 1, 1, 1, 1, 1, 1, 1, 1, 1, 1, 1,

1, 1, 1, 1, 1, 1, 1, 1, 1, 1, 1, 1, 1, 1, 1, 1, 1, 1, 1, 1,

1, 1, 1, 1, 1, 1, 1, 1, 1, 1, 1, 1, 1, 1, 1, 1, 1, 1, 1, 1,

1, 1, 1, 1, 1, 1, 1, 1, 1, 1, 1, 1, 1, 1, 1, 1, 1, 1, 1, 1,

1, 1, 1, 1, 1, 1, 1, 1, 1, 1, 1, 1, 1, 1, 1, 1, 1, 1, 1, 1,

1, 1, 1, 1, 1, 1, 1, 1, 1, 1, 1, 1, 1, 1, 1, 1, 1, 1, 1, 1,

1, 1, 1, 1, 1, 1, 1, 1, 1, 1, 1, 1, 1, 1, 1, 1, 1, 1, 1, 1,

1, 1, 1, 1, 1, 1, 1, 1, 1, 1, 1, 1, 1, 1, 1, 1, 1, 1, NA, NA,

NA, NA, NA, NA, NA, NA, NA, NA, NA, NA, NA, NA, NA, NA, NA, NA, NA, NA, NA, NA,

NA, NA, NA, NA, NA, NA, NA, NA, NA, NA, NA, NA, NA, NA, NA, NA, NA, NA, NA, NA,

NA, NA, NA, NA, NA, NA, NA, NA, NA, NA, NA, NA, NA, NA, NA, NA, NA, NA, NA, NA,

NA, NA, NA, NA, NA, NA, NA, NA, NA, NA, NA, NA, NA, NA, NA, NA, NA, NA, NA, NA,

NA, NA, NA, NA, NA, NA, NA, NA, NA, NA, NA, NA, NA, NA, NA, NA, NA, NA, NA, NA,

NA, NA, NA, NA, NA, NA, NA, NA, NA, NA, NA, NA, NA, NA, NA, NA, NA, NA, NA, NA,

NA, NA, NA, NA, NA, NA, NA, NA, NA, NA, NA, NA, NA, NA, NA, NA, NA, NA, NA, NA,

NA, NA, NA, NA, NA, NA, NA, NA, NA, NA, NA, NA, NA, NA, NA, NA, NA, NA, NA, NA,

NA, NA, NA, NA, NA, NA, NA, NA, NA, NA, NA, NA, NA, NA, NA, NA, NA, NA, NA, NA,

NA, NA, NA, NA, NA, NA, NA, NA, NA, NA, NA, NA, NA, NA, NA, NA, NA, NA, NA, NA,

NA, NA, NA, NA, NA, NA, NA, NA, NA, NA, NA, NA, NA, NA, NA, NA, NA, NA, NA, NA,

NA, NA, NA, NA, NA, NA, NA, NA, NA, NA, NA, NA, NA, NA, NA, NA, NA, NA, NA, NA,

NA, NA, NA, NA, NA, NA, NA, NA, NA, NA, NA, NA, NA, NA, NA, NA, NA, NA, NA, NA,

NA, NA, NA, NA, NA, NA, NA, NA, NA, NA, NA, NA, NA, NA, NA, NA, NA, NA, NA, NA,

NA, NA, NA, NA, NA, NA, NA, NA, NA, NA, NA, NA, NA, NA, NA, NA, NA, NA, NA, NA,

NA, NA, NA, NA, NA, NA, NA, NA, NA, NA, NA, NA, NA, NA, NA, NA, NA, NA, NA, NA,

NA, NA, NA, NA, NA, NA, NA, NA, NA, NA, NA, NA, NA, NA, NA, NA, NA, NA, NA, NA,

NA, NA, NA, NA, NA, NA, NA, NA, NA, NA, NA, NA, NA, NA, NA, NA, NA, NA, NA, NA,

NA, NA, NA, NA, NA, NA, NA, NA, NA, NA, NA, NA, NA, NA, NA, NA, NA, NA, NA, NA,

NA, NA, NA, NA, NA, NA, NA, NA, NA, NA, NA, NA, NA, NA, NA, NA, NA, NA, NA, NA,

NA, NA, NA, NA, NA, NA, NA, NA, NA, NA, NA, NA, NA, NA, NA, NA, NA, NA, NA, NA,

NA, NA, NA, NA, NA, NA, NA, NA, NA, NA, NA, NA, NA, NA, NA, NA, NA, NA, NA, NA,

NA, NA, NA, NA, NA, NA, NA, NA, NA, NA, NA, NA, NA, NA, NA, NA, NA, NA, NA, NA,

NA, NA, NA, NA, NA, NA, NA, NA, NA, NA, NA, NA, NA, NA, NA, NA, NA, NA, NA, NA,

NA, NA, NA, NA, NA, NA, NA, NA, NA, NA, NA, NA, NA, NA, NA, NA, NA, NA, NA, NA,

NA, NA, NA, NA, NA, NA, NA, NA, NA, NA, NA, NA, NA, NA, NA, NA, NA, NA, NA, NA,

NA, NA, NA, NA, NA, NA, NA, NA, NA, NA, NA, NA, NA, NA, NA, NA, NA, NA, NA, NA,

#F BNA85-90A

1, 1, 1, 1, 1, 1, 1, 1, 1, 1, 1, 1, 1, 1, 1, 1, 1, 1, 1, 1,

1, 1, 1, 1, 1, 1, 1, 1, 1, 1, 1, 1, 1, 1, 1, 1, 1, 1, 1, 1,

1, 1, 1, 1, 1, 1, 1, 1, 1, 1, 1, 1, 1, 1, 1, 1, 1, 1, 1, 1,

1, 1, 1, 1, 1, 1, 1, 1, 1, 1, 1, 1, 1, 1, 1, 1, 1, 1, 1, 1,

1, 1, 1, 1, 1, 1, 1, 1, 1, 1, 1, 1, 1, 1, 1, 1, 1, 1, 1, 1,

1, 1, 1, 1, 1, 1, 1, 1, 1, 1, 1, 1, 1, 1, 1, 1, 1, 1, 1, 1,

1, 1, 1, 1, 1, 1, 1, 1, 1, 1, 1, 1, 1, 1, 1, 1, 1, 1, 1, 1,

1, 1, 1, 1, 1, 1, 1, 1, 1, 1, 1, 1, 1, 1, 1, 1, 1, 1, 1, 1,

1, 1, 1, 1, 1, 1, 1, 1, 1, 1, 1, 1, 1, 1, 1, 1, 1, 1, 1, 1,

1, 1, 1, 1, 1, 1, 1, 1, 1, 1, 1, 1, 1, 1, 1, 1, 1, 1, 1, 1,

1, 1, 1, 1, 1, 1, 1, 1, 1, 1, 1, 1, 1, 1, 1, 1, 1, 1, 1, 1,

1, 1, 1, 1, 1, 1, 1, 1, 1, 1, 1, 1, 1, 1, 1, 1, 1, 1, 1, 1,

1, 1, 1, 1, 1, 1, 1, 1, 1, 1, 1, 1, 1, 1, 1, 1, 1, 1, 1, 1,

1, 1, 1, 1, 1, 1, 1, 1, 1, 1, 1, 1, 1, 1, 1, 1, 1, 1, 1, 1,

1, 1, 1, 1, 1, 1, 1, 1, 1, 1, 1, 1, 1, 1, 1, 1, 1, 1, 1, 1,

1, 1, 1, 1, 1, 1, 1, 1, 1, 1, 1, 1, 1, 1, 1, 1, 1, 1, 1, 1,

1, 1, 1, 1, 1, 1, 1, 1, 1, 1, 1, 1, 1, 1, 1, 1, 1, 1, 1, 1,

1, 1, 1, 1, 1, 1, 1, 1, 1, 1, 1, 1, 1, 1, 1, 1, 1, 1, 1, 1,

1, 1, 1, 1, 1, 1, 1, 1, 1, 1, 1, 1, 1, 1, 1, 1, 1, 1, 1, 1,

1, 1, 1, 1, 1, 1, 1, 1, 1, 1, 1, 1, 1, 1, 1, 1, 1, 1, 1, 1,

1, 1, 1, 1, 1, 1, 1, 1, 1, 1, 1, 1, 1, 1, 1, 1, 1, 1, 1, 1,

1, 1, 1, 1, 1, 1, 1, 1, 1, 1, 1, 1, 1, 1, 1, 1, 1, 1, 1, 1,

1, 1, 1, 1, 1, 1, 1, 1, 1, 1, 1, 1, 1, 1, 1, 1, 1, 1, 1, 1,

1, 1, 1, 1, 1, 1, 1, 1, 1, 1, 1, 1, 1, 1, 1, 1, 1, 1, 1, 1,

1, 1, 1, 1, 1, 1, 1, 1, 1, 1, 1, 1, 1, 1, 1, 1, 1, 1, 1, 1,

1, 1, 1, 1, 1, 1, 1, 1, 1, 1, 1, 1, 1, 1, 1, 1, 1, 1, 1, 1,

1, 1, 1, 1, 1, 1, 1, 1, 1, 1, 1, 1, 1, 1, 1, 1, 1, 1, 1, 1,

1, 1, 1, 1, 1, 1, 1, 1, 1, 1, 1, 1, 1, 1, 1, 1, 1, 1, 1, NA,

NA, NA, NA, NA, NA, NA, NA, NA, NA, NA, NA, NA, NA, NA, NA, NA, NA, NA, NA, NA,

NA, NA, NA, NA, NA, NA, NA, NA, NA, NA, NA, NA, NA, NA, NA, NA, NA, NA, NA, NA,

NA, NA, NA, NA, NA, NA, NA, NA, NA, NA, NA, NA, NA, NA, NA, NA, NA, NA, NA, NA,

NA, NA, NA, NA, NA, NA, NA, NA, NA, NA, NA, NA, NA, NA, NA, NA, NA, NA, NA, NA,

NA, NA, NA, NA, NA, NA, NA, NA, NA, NA, NA, NA, NA, NA, NA, NA, NA, NA, NA, NA,

NA, NA, NA, NA, NA, NA, NA, NA, NA, NA, NA, NA, NA, NA, NA, NA, NA, NA, NA, NA,

NA, NA, NA, NA, NA, NA, NA, NA, NA, NA, NA, NA, NA, NA, NA, NA, NA, NA, NA, NA,

NA, NA, NA, NA, NA, NA, NA, NA, NA, NA, NA, NA, NA, NA, NA, NA, NA, NA, NA, NA,

NA, NA, NA, NA, NA, NA, NA, NA, NA, NA, NA, NA, NA, NA, NA, NA, NA, NA, NA, NA,

NA, NA, NA, NA, NA, NA, NA, NA, NA, NA, NA, NA, NA, NA, NA, NA, NA, NA, NA, NA,

NA, NA, NA, NA, NA, NA, NA, NA, NA, NA, NA, NA, NA, NA, NA, NA, NA, NA, NA, NA,

NA, NA, NA, NA, NA, NA, NA, NA, NA, NA, NA, NA, NA, NA, NA, NA, NA, NA, NA, NA,

NA, NA, NA, NA, NA, NA, NA, NA, NA, NA, NA, NA, NA, NA, NA, NA, NA, NA, NA, NA,

NA, NA, NA, NA, NA, NA, NA, NA, NA, NA, NA, NA, NA, NA, NA, NA, NA, NA, NA, NA,

NA, NA, NA, NA, NA, NA, NA, NA, NA, NA, NA, NA, NA, NA, NA, NA, NA, NA, NA, NA,

NA, NA, NA, NA, NA, NA, NA, NA, NA, NA, NA, NA, NA, NA, NA, NA, NA, NA, NA, NA,

NA, NA, NA, NA, NA, NA, NA, NA, NA, NA, NA, NA, NA, NA, NA, NA, NA, NA, NA, NA,

NA, NA, NA, NA, NA, NA, NA, NA, NA, NA, NA, NA, NA, NA, NA, NA, NA, NA, NA, NA,

NA, NA, NA, NA, NA, NA, NA, NA, NA, NA, NA, NA, NA, NA, NA, NA, NA, NA, NA, NA,

NA, NA, NA, NA, NA, NA, NA, NA, NA, NA, NA, NA, NA, NA, NA, NA, NA, NA, NA, NA,

NA, NA, NA, NA, NA, NA, NA, NA, NA, NA, NA, NA, NA, NA, NA, NA, NA, NA, NA, NA,

NA, NA, NA, NA, NA, NA, NA, NA, NA, NA, NA, NA, NA, NA, NA, NA, NA, NA, NA, NA,

NA, NA, NA, NA, NA, NA, NA, NA, NA, NA, NA, NA, NA, NA, NA, NA, NA, NA, NA, NA,

NA, NA, NA, NA, NA, NA, NA, NA, NA, NA, NA, NA, NA, NA, NA, NA, NA, NA, NA, NA,

NA, NA, NA, NA, NA, NA, NA, NA, NA, NA, NA, NA, NA, NA, NA, NA, NA, NA, NA, NA,

NA, NA, NA, NA, NA, NA, NA, NA, NA, NA, NA, NA, NA, NA, NA, NA, NA, NA, NA, NA,

NA, NA, NA, NA, NA, NA, NA, NA, NA, NA, NA, NA, NA, NA, NA, NA, NA, NA, NA, NA,

NA, NA, NA, NA, NA, NA, NA, NA, NA, NA, NA, NA, NA, NA, NA, NA, NA, NA, NA, NA,

NA, NA, NA, NA, NA, NA, NA, NA, NA, NA, NA, NA, NA, NA, NA, NA, NA, NA, NA, NA,

NA, NA, NA, NA, NA, NA, NA, NA, NA, NA, NA, NA, NA, NA, NA, NA, NA, NA, NA, NA,

NA, NA, NA, NA, NA, NA, NA, NA, NA, NA, NA, NA, NA, NA, NA, NA, NA, NA, NA, NA,

NA, NA, NA, NA, NA, NA, NA, NA, NA, NA, NA, NA, NA, NA, NA, NA, NA, NA, NA, NA,

NA, NA, NA, NA, NA, NA, NA, NA, NA, NA, NA, NA, NA, NA, NA, NA, NA, NA, NA, NA,

NA, NA, NA, NA, NA, NA, NA, NA, NA, NA, NA, NA, NA, NA, NA, NA, NA, NA, NA, NA,

NA, NA, NA, NA, NA, NA, NA, NA, NA, NA, NA, NA, NA, NA, NA, NA, NA, NA, NA, NA,

NA, NA, NA, NA, NA, NA, NA, NA, NA, NA, NA, NA, NA, NA, NA, NA, NA, NA, NA, NA,

NA, NA, NA, NA, NA, NA, NA, NA, NA, NA, NA, NA, NA, NA, NA, NA, NA, NA, NA, NA,

NA, NA, NA, NA, NA, NA, NA, NA, NA, NA, NA, NA, NA, NA, NA, NA, NA, NA, NA, NA,

NA, NA, NA, NA, NA, NA, NA, NA, NA, NA, NA, NA, NA, NA, NA, NA, NA, NA, NA, NA,

NA, NA, NA, NA, NA, NA, NA, NA, NA, NA, NA, NA, NA, NA, NA, NA, NA, NA, NA, NA,

NA, NA, NA, NA, NA, NA, NA, NA, NA, NA, NA, NA, NA, NA, NA, NA, NA, NA, NA, NA,

NA, NA, NA, NA, NA, NA, NA, NA, NA, NA, NA, NA, NA, NA, NA, NA, NA, NA, NA, NA,

#F HIC 90+

1, 1, 1, 1, 1, 1, 1, 1, 1, 1, 1, 1, 1, 1, 1, 1, 1, 1, 1, 1,

1, 1, 1, 1, 1, 1, 1, 1, 1, 1, 1, 1, 1, 1, 1, 1, 1, 1, 1, 1,

1, 1, 1, 1, 1, 1, 1, 1, 1, 1, 1, 1, 1, 1, 1, 1, 1, 1, 1, 1,

1, 1, 1, 1, 1, 1, 1, 1, 1, 1, 1, 1, 1, 1, 1, 1, 1, 1, 1, 1,

1, 1, 1, 1, 1, 1, 1, 1, 1, 1, 1, 1, 1, 1, 1, 1, 1, 1, 1, 1,

1, 1, 1, 1, 1, 1, 1, 1, 1, 1, 1, 1, 1, 1, 1, 1, 1, 1, 1, 1,

1, 1, 1, 1, 1, 1, 1, 1, 1, 1, 1, 1, 1, 1, 1, 1, 1, 1, 1, 1,

1, 1, 1, 1, 1, 1, 1, 1, 1, 1, 1, 1, 1, 1, 1, 1, 1, 1, 1, 1,

1, 1, 1, 1, 1, 1, 1, 1, 1, 1, 1, 1, 1, 1, 1, 1, 1, 1, 1, 1,

1, 1, 1, 1, 1, 1, 1, 1, 1, 1, 1, 1, 1, 1, 1, 1, 1, 1, 1, 1,

1, 1, 1, 1, 1, 1, 1, 1, 1, 1, 1, 1, 1, 1, 1, 1, 1, 1, 1, 1,

1, 1, 1, 1, 1, 1, 1, 1, 1, 1, 1, 1, 1, 1, 1, 1, 1, 1, 1, 1,

1, 1, 1, 1, 1, 1, 1, 1, 1, 1, 1, 1, 1, 1, 1, 1, 1, 1, 1, 1,

1, 1, 1, 1, 1, 1, 1, 1, 1, 1, 1, 1, 1, 1, 1, 1, 1, 1, 1, 1,

1, 1, 1, 1, 1, 1, 1, 1, 1, 1, 1, 1, 1, 1, 1, 1, 1, 1, 1, 1,

1, 1, 1, 1, 1, 1, 1, 1, 1, 1, 1, 1, 1, 1, 1, 1, 1, 1, 1, 1,

1, 1, 1, 1, 1, 1, 1, 1, 1, 1, 1, 1, 1, 1, 1, 1, 1, 1, 1, 1,

1, 1, 1, 1, 1, 1, 1, 1, 1, 1, 1, 1, 1, 1, 1, 1, 1, 1, 1, 1,

1, 1, 1, 1, 1, 1, 1, 1, 1, 1, 1, 1, 1, 1, 1, 1, 1, 1, 1, 1,

1, 1, 1, 1, 1, 1, 1, 1, 1, 1, 1, 1, 1, 1, 1, 1, 1, 1, 1, 1,

1, 1, 1, 1, 1, 1, 1, 1, 1, 1, 1, 1, 1, 1, 1, 1, 1, 1, 1, 1,

1, 1, 1, 1, 1, 1, 1, 1, 1, 1, 1, 1, 1, 1, 1, 1, 1, 1, 1, 1,

1, 1, 1, 1, 1, 1, 1, 1, 1, 1, 1, 1, 1, 1, 1, 1, 1, 1, 1, 1,

1, 1, 1, 1, 1, 1, 1, 1, 1, 1, 1, 1, 1, 1, 1, 1, 1, 1, 1, 1,

1, 1, 1, 1, 1, 1, 1, 1, 1, 1, 1, 1, 1, 1, 1, 1, 1, 1, 1, 1,

1, 1, 1, 1, 1, 1, 1, 1, 1, 1, 1, 1, 1, 1, 1, 1, 1, 1, 1, 1,

1, 1, 1, 1, 1, 1, 1, 1, 1, 1, 1, 1, 1, 1, 1, 1, 1, 1, 1, 1,

1, 1, 1, 1, 1, 1, 1, 1, 1, 1, 1, 1, 1, 1, 1, 1, 1, 1, 1, 1,

1, 1, 1, 1, 1, 1, 1, 1, 1, 1, 1, 1, 1, 1, 1, 1, 1, 1, 1, 1,

1, 1, 1, 1, 1, 1, 1, 1, 1, 1, 1, 1, 1, 1, 1, 1, 1, 1, 1, 1,

1, 1, 1, 1, 1, 1, 1, 1, 1, 1, 1, 1, 1, 1, 1, 1, 1, 1, 1, 1,

1, 1, 1, 1, 1, 1, 1, 1, 1, 1, 1, 1, 1, 1, 1, 1, 1, 1, 1, 1,

1, 1, NA, NA, NA, NA, NA, NA, NA, NA, NA, NA, NA, NA, NA, NA, NA, NA, NA, NA,

NA, NA, NA, NA, NA, NA, NA, NA, NA, NA, NA, NA, NA, NA, NA, NA, NA, NA, NA, NA,

NA, NA, NA, NA, NA, NA, NA, NA, NA, NA, NA, NA, NA, NA, NA, NA, NA, NA, NA, NA,

NA, NA, NA, NA, NA, NA, NA, NA, NA, NA, NA, NA, NA, NA, NA, NA, NA, NA, NA, NA,

NA, NA, NA, NA, NA, NA, NA, NA, NA, NA, NA, NA, NA, NA, NA, NA, NA, NA, NA, NA,

NA, NA, NA, NA, NA, NA, NA, NA, NA, NA, NA, NA, NA, NA, NA, NA, NA, NA, NA, NA,

NA, NA, NA, NA, NA, NA, NA, NA, NA, NA, NA, NA, NA, NA, NA, NA, NA, NA, NA, NA,

NA, NA, NA, NA, NA, NA, NA, NA, NA, NA, NA, NA, NA, NA, NA, NA, NA, NA, NA, NA,

NA, NA, NA, NA, NA, NA, NA, NA, NA, NA, NA, NA, NA, NA, NA, NA, NA, NA, NA, NA,

NA, NA, NA, NA, NA, NA, NA, NA, NA, NA, NA, NA, NA, NA, NA, NA, NA, NA, NA, NA,

NA, NA, NA, NA, NA, NA, NA, NA, NA, NA, NA, NA, NA, NA, NA, NA, NA, NA, NA, NA,

NA, NA, NA, NA, NA, NA, NA, NA, NA, NA, NA, NA, NA, NA, NA, NA, NA, NA, NA, NA,

NA, NA, NA, NA, NA, NA, NA, NA, NA, NA, NA, NA, NA, NA, NA, NA, NA, NA, NA, NA,

NA, NA, NA, NA, NA, NA, NA, NA, NA, NA, NA, NA, NA, NA, NA, NA, NA, NA, NA, NA,

NA, NA, NA, NA, NA, NA, NA, NA, NA, NA, NA, NA, NA, NA, NA, NA, NA, NA, NA, NA,

NA, NA, NA, NA, NA, NA, NA, NA, NA, NA, NA, NA, NA, NA, NA, NA, NA, NA, NA, NA,

NA, NA, NA, NA, NA, NA, NA, NA, NA, NA, NA, NA, NA, NA, NA, NA, NA, NA, NA, NA,

NA, NA, NA, NA, NA, NA, NA, NA, NA, NA, NA, NA, NA, NA, NA, NA, NA, NA, NA, NA,

NA, NA, NA, NA, NA, NA, NA, NA, NA, NA, NA, NA, NA, NA, NA, NA, NA, NA, NA, NA,

NA, NA, NA, NA, NA, NA, NA, NA, NA, NA, NA, NA, NA, NA, NA, NA, NA, NA, NA, NA,

NA, NA, NA, NA, NA, NA, NA, NA, NA, NA, NA, NA, NA, NA, NA, NA, NA, NA, NA, NA,

NA, NA, NA, NA, NA, NA, NA, NA, NA, NA, NA, NA, NA, NA, NA, NA, NA, NA, NA, NA,

NA, NA, NA, NA, NA, NA, NA, NA, NA, NA, NA, NA, NA, NA, NA, NA, NA, NA, NA, NA,

NA, NA, NA, NA, NA, NA, NA, NA, NA, NA, NA, NA, NA, NA, NA, NA, NA, NA, NA, NA,

NA, NA, NA, NA, NA, NA, NA, NA, NA, NA, NA, NA, NA, NA, NA, NA, NA, NA, NA, NA,

NA, NA, NA, NA, NA, NA, NA, NA, NA, NA, NA, NA, NA, NA, NA, NA, NA, NA, NA, NA,

NA, NA, NA, NA, NA, NA, NA, NA, NA, NA, NA, NA, NA, NA, NA, NA, NA, NA, NA, NA,

NA, NA, NA, NA, NA, NA, NA, NA, NA, NA, NA, NA, NA, NA, NA, NA, NA, NA, NA, NA,

NA, NA, NA, NA, NA, NA, NA, NA, NA, NA, NA, NA, NA, NA, NA, NA, NA, NA, NA, NA,

NA, NA, NA, NA, NA, NA, NA, NA, NA, NA, NA, NA, NA, NA, NA, NA, NA, NA, NA, NA,

NA, NA, NA, NA, NA, NA, NA, NA, NA, NA, NA, NA, NA, NA, NA, NA, NA, NA, NA, NA,

NA, NA, NA, NA, NA, NA, NA, NA, NA, NA, NA, NA, NA, NA, NA, NA, NA, NA, NA, NA,

NA, NA, NA, NA, NA, NA, NA, NA, NA, NA, NA, NA, NA, NA, NA, NA, NA, NA, NA, NA,

NA, NA, NA, NA, NA, NA, NA, NA, NA, NA, NA, NA, NA, NA, NA, NA, NA, NA, NA, NA,

NA, NA, NA, NA, NA, NA, NA, NA, NA, NA, NA, NA, NA, NA, NA, NA, NA, NA, NA, NA,

NA, NA, NA, NA, NA, NA, NA, NA, NA, NA, NA, NA, NA, NA, NA, NA, NA, NA, NA, NA,

NA, NA, NA, NA, NA, NA, NA, NA, NA, NA, NA, NA, NA, NA, NA, NA, NA, NA, NA, NA,

NA, NA, NA, NA, NA, NA, NA, NA, NA, NA, NA, NA, NA, NA, NA, NA, NA, NA, NA, NA,

#F BNA90+

1, 1, 1, 1, 1, 1, 1, 1, 1, 1, 1, 1, 1, 1, 1, 1, 1, 1, 1, 1,

1, 1, 1, 1, 1, 1, 1, 1, 1, 1, 1, 1, 1, 1, 1, 1, 1, 1, 1, 1,

1, 1, 1, 1, 1, 1, 1, 1, 1, 1, 1, 1, 1, 1, 1, 1, 1, 1, 1, 1,

1, 1, 1, 1, 1, 1, 1, 1, 1, 1, 1, 1, 1, 1, 1, 1, 1, 1, 1, 1,

1, 1, 1, 1, 1, 1, 1, 1, 1, 1, 1, 1, 1, 1, 1, 1, 1, 1, 1, 1,

1, 1, 1, 1, 1, 1, 1, 1, 1, 1, 1, 1, 1, 1, 1, 1, 1, 1, 1, 1,

1, 1, 1, 1, 1, 1, 1, 1, 1, 1, 1, 1, 1, 1, 1, 1, 1, 1, 1, 1,

1, 1, 1, 1, 1, 1, 1, 1, 1, 1, 1, 1, 1, 1, 1, 1, 1, 1, 1, 1,

1, 1, 1, 1, 1, 1, 1, 1, 1, 1, 1, 1, 1, 1, 1, 1, 1, 1, 1, 1,

1, 1, 1, 1, 1, 1, 1, 1, 1, 1, 1, 1, 1, 1, 1, 1, 1, 1, 1, 1,

1, 1, 1, 1, 1, 1, 1, 1, 1, 1, 1, 1, 1, 1, 1, 1, 1, 1, 1, 1,

1, 1, 1, 1, 1, 1, 1, 1, 1, 1, 1, 1, 1, 1, 1, 1, 1, 1, NA, NA,

NA, NA, NA, NA, NA, NA, NA, NA, NA, NA, NA, NA, NA, NA, NA, NA, NA, NA, NA, NA,

NA, NA, NA, NA, NA, NA, NA, NA, NA, NA, NA, NA, NA, NA, NA, NA, NA, NA, NA, NA,

NA, NA, NA, NA, NA, NA, NA, NA, NA, NA, NA, NA, NA, NA, NA, NA, NA, NA, NA, NA,

NA, NA, NA, NA, NA, NA, NA, NA, NA, NA, NA, NA, NA, NA, NA, NA, NA, NA, NA, NA,

NA, NA, NA, NA, NA, NA, NA, NA, NA, NA, NA, NA, NA, NA, NA, NA, NA, NA, NA, NA,

NA, NA, NA, NA, NA, NA, NA, NA, NA, NA, NA, NA, NA, NA, NA, NA, NA, NA, NA, NA,

NA, NA, NA, NA, NA, NA, NA, NA, NA, NA, NA, NA, NA, NA, NA, NA, NA, NA, NA, NA,

NA, NA, NA, NA, NA, NA, NA, NA, NA, NA, NA, NA, NA, NA, NA, NA, NA, NA, NA, NA,

NA, NA, NA, NA, NA, NA, NA, NA, NA, NA, NA, NA, NA, NA, NA, NA, NA, NA, NA, NA,

NA, NA, NA, NA, NA, NA, NA, NA, NA, NA, NA, NA, NA, NA, NA, NA, NA, NA, NA, NA,

NA, NA, NA, NA, NA, NA, NA, NA, NA, NA, NA, NA, NA, NA, NA, NA, NA, NA, NA, NA,

NA, NA, NA, NA, NA, NA, NA, NA, NA, NA, NA, NA, NA, NA, NA, NA, NA, NA, NA, NA,

NA, NA, NA, NA, NA, NA, NA, NA, NA, NA, NA, NA, NA, NA, NA, NA, NA, NA, NA, NA,

NA, NA, NA, NA, NA, NA, NA, NA, NA, NA, NA, NA, NA, NA, NA, NA, NA, NA, NA, NA,

NA, NA, NA, NA, NA, NA, NA, NA, NA, NA, NA, NA, NA, NA, NA, NA, NA, NA, NA, NA,

NA, NA, NA, NA, NA, NA, NA, NA, NA, NA, NA, NA, NA, NA, NA, NA, NA, NA, NA, NA,

NA, NA, NA, NA, NA, NA, NA, NA, NA, NA, NA, NA, NA, NA, NA, NA, NA, NA, NA, NA,

NA, NA, NA, NA, NA, NA, NA, NA, NA, NA, NA, NA, NA, NA, NA, NA, NA, NA, NA, NA,

NA, NA, NA, NA, NA, NA, NA, NA, NA, NA, NA, NA, NA, NA, NA, NA, NA, NA, NA, NA,

NA, NA, NA, NA, NA, NA, NA, NA, NA, NA, NA, NA, NA, NA, NA, NA, NA, NA, NA, NA,

NA, NA, NA, NA, NA, NA, NA, NA, NA, NA, NA, NA, NA, NA, NA, NA, NA, NA, NA, NA,

NA, NA, NA, NA, NA, NA, NA, NA, NA, NA, NA, NA, NA, NA, NA, NA, NA, NA, NA, NA,

NA, NA, NA, NA, NA, NA, NA, NA, NA, NA, NA, NA, NA, NA, NA, NA, NA, NA, NA, NA,

NA, NA, NA, NA, NA, NA, NA, NA, NA, NA, NA, NA, NA, NA, NA, NA, NA, NA, NA, NA,

NA, NA, NA, NA, NA, NA, NA, NA, NA, NA, NA, NA, NA, NA, NA, NA, NA, NA, NA, NA,

NA, NA, NA, NA, NA, NA, NA, NA, NA, NA, NA, NA, NA, NA, NA, NA, NA, NA, NA, NA,

NA, NA, NA, NA, NA, NA, NA, NA, NA, NA, NA, NA, NA, NA, NA, NA, NA, NA, NA, NA,

NA, NA, NA, NA, NA, NA, NA, NA, NA, NA, NA, NA, NA, NA, NA, NA, NA, NA, NA, NA,

NA, NA, NA, NA, NA, NA, NA, NA, NA, NA, NA, NA, NA, NA, NA, NA, NA, NA, NA, NA,

NA, NA, NA, NA, NA, NA, NA, NA, NA, NA, NA, NA, NA, NA, NA, NA, NA, NA, NA, NA,

NA, NA, NA, NA, NA, NA, NA, NA, NA, NA, NA, NA, NA, NA, NA, NA, NA, NA, NA, NA,

NA, NA, NA, NA, NA, NA, NA, NA, NA, NA, NA, NA, NA, NA, NA, NA, NA, NA, NA, NA,

NA, NA, NA, NA, NA, NA, NA, NA, NA, NA, NA, NA, NA, NA, NA, NA, NA, NA, NA, NA,

NA, NA, NA, NA, NA, NA, NA, NA, NA, NA, NA, NA, NA, NA, NA, NA, NA, NA, NA, NA,

NA, NA, NA, NA, NA, NA, NA, NA, NA, NA, NA, NA, NA, NA, NA, NA, NA, NA, NA, NA,

NA, NA, NA, NA, NA, NA, NA, NA, NA, NA, NA, NA, NA, NA, NA, NA, NA, NA, NA, NA,

NA, NA, NA, NA, NA, NA, NA, NA, NA, NA, NA, NA, NA, NA, NA, NA, NA, NA, NA, NA,

NA, NA, NA, NA, NA, NA, NA, NA, NA, NA, NA, NA, NA, NA, NA, NA, NA, NA, NA, NA,

NA, NA, NA, NA, NA, NA, NA, NA, NA, NA, NA, NA, NA, NA, NA, NA, NA, NA, NA, NA,

NA, NA, NA, NA, NA, NA, NA, NA, NA, NA, NA, NA, NA, NA, NA, NA, NA, NA, NA, NA,

NA, NA, NA, NA, NA, NA, NA, NA, NA, NA, NA, NA, NA, NA, NA, NA, NA, NA, NA, NA,

NA, NA, NA, NA, NA, NA, NA, NA, NA, NA, NA, NA, NA, NA, NA, NA, NA, NA, NA, NA,

NA, NA, NA, NA, NA, NA, NA, NA, NA, NA, NA, NA, NA, NA, NA, NA, NA, NA, NA, NA,

NA, NA, NA, NA, NA, NA, NA, NA, NA, NA, NA, NA, NA, NA, NA, NA, NA, NA, NA, NA,

NA, NA, NA, NA, NA, NA, NA, NA, NA, NA, NA, NA, NA, NA, NA, NA, NA, NA, NA, NA,

NA, NA, NA, NA, NA, NA, NA, NA, NA, NA, NA, NA, NA, NA, NA, NA, NA, NA, NA, NA,

NA, NA, NA, NA, NA, NA, NA, NA, NA, NA, NA, NA, NA, NA, NA, NA, NA, NA, NA, NA,

NA, NA, NA, NA, NA, NA, NA, NA, NA, NA, NA, NA, NA, NA, NA, NA, NA, NA, NA, NA,

NA, NA, NA, NA, NA, NA, NA, NA, NA, NA, NA, NA, NA, NA, NA, NA, NA, NA, NA, NA,

NA, NA, NA, NA, NA, NA, NA, NA, NA, NA, NA, NA, NA, NA, NA, NA, NA, NA, NA, NA,

NA, NA, NA, NA, NA, NA, NA, NA, NA, NA, NA, NA, NA, NA, NA, NA, NA, NA, NA, NA,

NA, NA, NA, NA, NA, NA, NA, NA, NA, NA, NA, NA, NA, NA, NA, NA, NA, NA, NA, NA,

NA, NA, NA, NA, NA, NA, NA, NA, NA, NA, NA, NA, NA, NA, NA, NA, NA, NA, NA, NA,

NA, NA, NA, NA, NA, NA, NA, NA, NA, NA, NA, NA, NA, NA, NA, NA, NA, NA, NA, NA,

NA, NA, NA, NA, NA, NA, NA, NA, NA, NA, NA, NA, NA, NA, NA, NA, NA, NA, NA, NA,

NA, NA, NA, NA, NA, NA, NA, NA, NA, NA, NA, NA, NA, NA, NA, NA, NA, NA, NA, NA,

NA, NA, NA, NA, NA, NA, NA, NA, NA, NA, NA, NA, NA, NA, NA, NA, NA, NA, NA, NA,

NA, NA, NA, NA, NA, NA, NA, NA, NA, NA, NA, NA, NA, NA, NA, NA, NA, NA, NA, NA

),.Dim=c(1400,12))),

The p[i,k] variable is the capture probability for each individual *i* during event k, T is the maximum number of possible captures. The x[i,k] vector of dimension M represents the number of times that the individuals were captured, i.e. 0 for non-captured individuals.
