# Supplementary material for: Alzheimer's disease: Estimating its prevalence rate in a French geographical unit using the National Alzheimer Data Bank and national health insurance information systems
Source: PLoS One. 2019 May 6;14(5):e0216221. doi: 10.1371/journal.pone.0216221 (PMC6502320; doi:10.1371/journal.pone.0216221)
Supplement: S2 Table — (DOCX) [file pone.0216221.s002.docx]

**S2 Table. Expected cases of Alzheimer’s disease in the Alpes-Maritimes population in 2010**

| **Men** | | | | **Women** | | | **Expected cases of Alzheimer’s disease** | |
| --- | --- | --- | --- | --- | --- | --- | --- | --- |
| Age | Population* | Prevalence  (%)** | N | Population* | Prévalence(%)** | N |  |  |
| <80 | 20 483 | 4,8 | 986 | 27 614 | 3,6 | 984 | | 1969 |
| 80-84 | 15 068 | 10,3 | 1558 | 24 669 | 13,7 | 3387 | | 4944 |
| 85-89 | 9 302 | 14,3 | 1762 | 18 139 | 12,6 | 3030 | | 4792 |
| >90 | 1 953 | 22,3 | 436 | 6 063 | 25,5 | 1544 | | 1980 |
| **Total** | 46 806 |  | 4741 | 76 485 |  | 8945 | | 13686 |

* Population count by age published by INSEE[20] ** Prevalence of Alzheimer’s disease adapted from PAQUID rates of dementia[1]
